# Design, Synthesis and Antitumor Activity of 1*H*-indazole-3-amine Derivatives

**DOI:** 10.3390/ijms24108686

**Published:** 2023-05-12

**Authors:** Congyu Wang, Mei Zhu, Xuesha Long, Qin Wang, Zhenchao Wang, Guiping Ouyang

**Affiliations:** 1 College of Pharmacy, Guizhou University, Guiyang 550025, China; 2 Guizhou Engineering Laboratory for Synthetic Drugs, Guizhou University, Guiyang 550025, China; 3 Center for Research and Development of Fine Chemicals, Guizhou University, Guiyang 550025, China

**Keywords:** indazole derivatives, antitumor activity, cell apoptosis, p53/MDM2 pathway

## Abstract

A series of indazole derivatives were designed and synthesized by molecular hybridization strategy, and these compounds were evaluated the inhibitory activities against human cancer cell lines of lung (A549), chronic myeloid leukemia (K562), prostate (PC-3), and hepatoma (Hep-G2) by methyl thiazolyl tetrazolium (MTT) colorimetric assay. Among these, compound **6o** exhibited a promising inhibitory effect against the K562 cell line with the IC_50_ (50% inhibition concentration) value of 5.15 µM, and this compound showed great selectivity for normal cell (HEK-293, IC_50_ = 33.2 µM). Moreover, compound **6o** was confirmed to affect apoptosis and cell cycle possibly by inhibiting Bcl2 family members and the p53/MDM2 pathway in a concentration-dependent manner. Overall, this study indicates that compound **6o** could be a promising scaffold to develop an effective and low-toxic anticancer agent.

## 1. Introduction

Cancer is a prevalent cause of mortality, and its treatment has been the subject of global scrutiny [1]. Compared with radiotherapy and biological therapy, chemotherapy remains the backbone of current treatment. However, the clinical application of chemotherapy drugs is limited owing to poor selectivity, drug resistance, and serious side-effect [2]. Consequently, developing novel anti-cancer drugs with high efficiency and low toxicity is still sorely needed.

**Figure 1:**
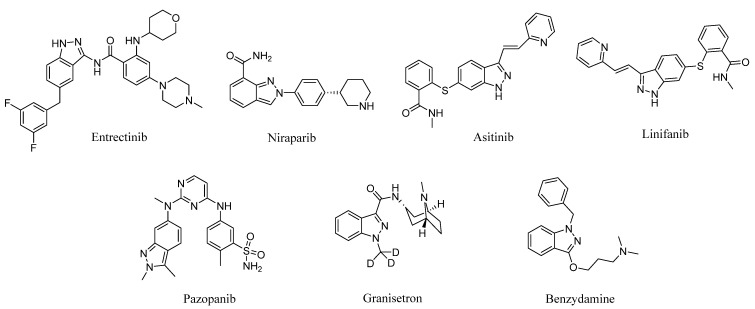
Chemical structure of marketing indazole drugs.

Nitrogenous heterocycles are an important part of many natural products and marketed drugs. Among this family, indazole compounds are one of the principal components [3]. Various substituted indazole derivatives have attracted great attention due to their diverse biological activities, such as anti-tumor, anti-inflammatory, anti-bacterial, anti-diabetes, and anti-osteoporosis [4,5,6]. At present, at least 43 kinds of indazole-based therapeutics have been used in clinical application or clinical trials [7,8]. For example, the representative compound shown in Figure 1, where Entrectinib is an anaplastic lymphoma kinase (ALK) inhibitor [9], Pazopanib is a multi-kinase inhibitor [10,11], Niraparib is a PARP1/PARP2 inhibitor [12], Linifanib is a multi-targeted ATP-competitive tyrosine kinase inhibitor [13], and Asitinib is an intravascular epidermal growth factor receptor (VEGFR) inhibitor [14]. Serotonin receptor antagonist Granisetron and non-steroidal anti-inflammatory drug Benzydamine are also used to treat vomiting caused by cancer chemotherapy [15,16]. The effective anti-cancer activity of these small molecules prompted us to further design indazole derivatives and hope to find some potential therapeutic agents.

**Figure 2:**
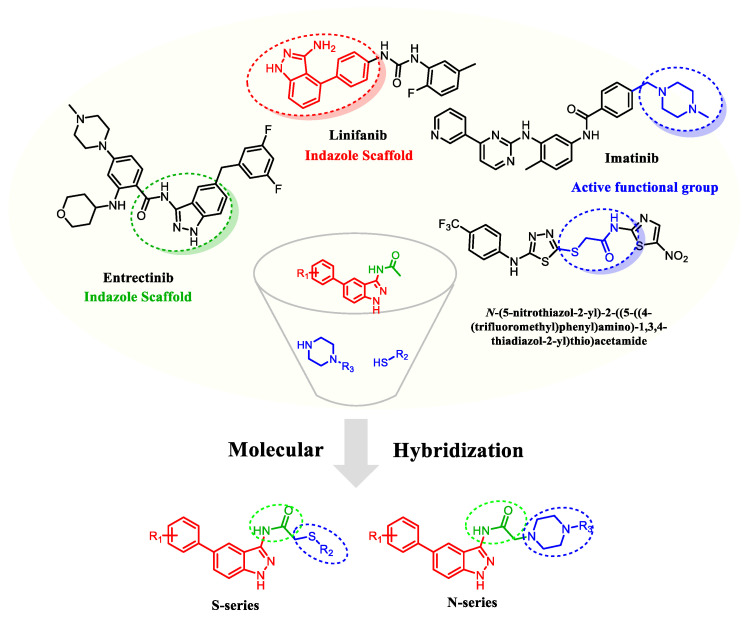
Design of the target compounds.

It has been demonstrated that the 1*H*-indazole-3-amine structure is an effective hinge-binding fragment [17], and in Linifanib, it binds effectively with the hinge region of tyrosine kinase [18]. In addition, the structure of 1*H*-indazole-3-amide in Entrectinib plays a critical role in enhancing the antitumor activity of the compound. Motivated by the aforementioned factors, our study focuses on further structural modifications of the 1*H*-indazole-3-amide framework. The mercapto acetamide moiety is commonly found in many cytotoxic drugs, such as in the active binding site of histone deacetylase inhibitors with significant interactions [19,20,21]. Furthermore, the introduction of piperazine can efficiently alter the pharmacokinetic parameters of drugs, modulate the acid-base equilibrium constants and lipid-water partition coefficients, and form intramolecular hydrogen and ionic bonds, thus improving the water solubility, alkalinity, and biological activity of compounds [22]. For example, Imatinib, a drug used for the treatment of leukemia, has a piperazine moiety as the fundamental functional group, which enhances solubility and oral bioavailability [23]. Therefore, we chose to introduce the active group mercapto acetamide or piperazine acetamide at the C-3 position of indazole. Moreover, in combination with the substitution characteristics of indazole derivatives, aromatic ring substitution at the C-5 position has received increasing attention to discover highly active and selective inhibitors [24]. The introduction of different substituted aromatic groups at the C-5 position through Suzuki coupling allows the simultaneous exploration of more targets of action with kinases, increasing the activity possibilities and exploring the effect of different substituents on the biological activity of the compounds [25,26]. Therefore, we constructed a series of 3,5-disubstituted indazole derivatives by molecular hybridization strategy (Figure 2), and evaluated their antitumor activity.

## 2. Results and Discussion

### 2.1. Chemistry

**Scheme 1:**
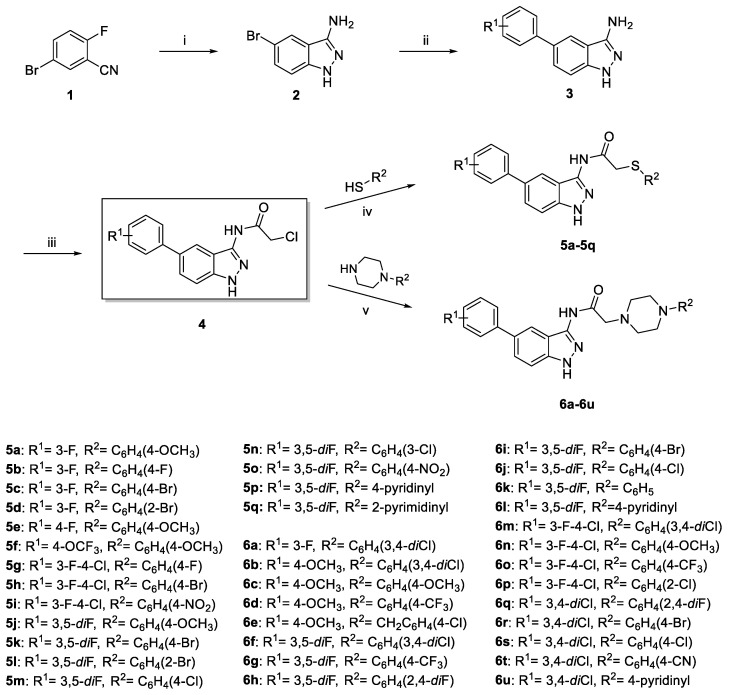
Reagents and conditions: (i) NH_2_·NH_2_·H_2_O (80%), reflux, 20 min, Yield: 88%; (ii) PdCl_2_(dppf)_2_, Cs_2_CO_3_, N_2_, 1,4-dioxane/H_2_O (1:1), 90 °C, 6 h, Yield: 75–80%; (iii) C_4_H_4_Cl_2_O_3_, Na_2_CO_3_, rt, 10 h, Yield: 62–68%; (iv) EtOH, KOH, rt, 12 h; (v) CH_3_CN, K_2_CO_3_, reflux, 5 h.

The general synthetic procedure for the target compounds was outlined in Scheme 1. The synthetic strategy begins with the 5-bromo-2-fluorobenzonitrile (**1**). Compound **1** was refluxed with hydrazine hydrate (80%) to afford 5-bromo-1*H*-indazol-3-amine **2** [27] with a high yield in only 20 min. Then, compound **2** was coupled with various substituted boronic acid esters to yield the Suzuki-coupling product **3** [28], The Suzuki reaction was carried out by employing C_S2_CO_3_ as base, 1,4-dioxane/H2O (1:1) as solvent and heating the reaction mixture at 90 °C and protecting by nitrogen. In the Suzuki reaction, PdCl_2_(dppf)_2_ is the catalyst, which could shorten the reaction time and increase the yield [29]. Subsequently, chloroacetic anhydride undergoes an acylation reaction with compound **3** under alkaline conditions to afford **4**. Finally, compound **4** with different thiophenols or piperazines was coupled in the presence of alkali to afford designed ethyl amide-linked indazole hybrids **5a**–**5q** and **6a**–**6u** (Scheme 1). The structure of target compounds was confirmed by ^1^H NMR spectroscopy, ^13^C NMR spectroscopy, and HR-MS spectroscopy (Supporting Information, Figures S1–S115).

Briefly analysis of the ^1^H NMR spectrum of the target compound: the single peaks appearing at chemical shifts *δ*: 10–13 ppm are the active hydrogens on the C-1 and C-3 amides of indazole; while the sets of peaks appearing at *δ*: 6–8 ppm are the aromatic hydrogens on indazole and benzene rings; while the sharp single peaks appearing at *δ*: 3–4 ppm are the two hydrogens on acetamide. Besides, in the spectroscopy of compounds **6a**–**6u**, the eight hydrogens on the piperazine moiety appear at *δ*: 2–4 ppm.

### 2.2. Anti-Proliferative Activity Analysis

The antiproliferative activity of the target indazole derivatives was evaluated against a panel of human cancer cell lines (human chronic myeloid leukemia cell lines K562; human lung cancer cell lines A549; human prostate cancer cell lines PC-3; human hepatoma cell lines HepG-2) in vitro, and human embryonic kidney cell lines HEK-293 were used to evaluate the cytotoxicity of a normal cell with high activity derivatives by MTT assay. Moreover, 5-fluorouracil (**5-Fu**), a broad-spectrum drug antitumor drug with therapeutic effects on various cancers, was used as a positive control.

To investigate the structure-activity relationships (SARS) of the synthesized compounds, all target compounds were used to evaluate the concentrations that cause 50% inhibition of cancer cell growth (IC_50_) on the four tumor cell lines. IC_50_ (µM) values of the compounds **5a**–**q** were displayed in Table 1. As a whole, the antitumor activity of the most mercapto-derived target compounds against Hep-G2 cells was superior to the other three tumor cells, among which, some compounds showed similar or superior to **5-Fu**. As shown in Table 1, the R^1^ substituent on the benzene ring at the C-5 position of indazole had a significant effect on the anti-proliferative activity of Hep-G2, which had a general trend in the order of 3,5-difluoro substituent (**5j**) > 4-fluoro substituent (**5e**) > 3-fluoro substituent (**5b**) > 4-trifluoromethoxy substituent (**5f**), indicating the importance of the interposition fluorine substituent for antitumor activity. Among the compounds, **5k** showed the best inhibitory effect against Hep-G2 (IC_50_ = 3.32 µM), however, this compound showed high toxicity to normal cells (HEK-293, IC_50_ = 12.17 µM), and its poor selectivity made it less attractive for further investigation. The high toxicity may have been caused by the mercapto acetamide group in this molecule.

Thus, we replaced the mercapto group with the piperazine group, hoping to improve the physicochemical properties of these molecules. Then, piperazine-indazole derivatives **6a**–**u** were synthesized, and the results of antitumor activity showed that most of these compounds exhibited significant antiproliferative activity against K562 cells (Table 2). Concluded from the preliminary results of cytotoxic potential inhibition assays in vitro, some meaningful SARs were obtained: (1) for the substituent (R^1^) at the C-5 position of indazole, when 3-fluorophenyl (**6a**) was replaced by 4-methoxyphenyl (**6b**–**e**) or 3,4-dichlorophenyl (**6q**–**u**), the inhibitory activity of the compound against K562 decreased to 2–10-fold, which is consistent with the **5a**–**q** series, where the presence of para-fluorine is crucial for antitumor activity. Additionally, keeping the interposition fluorine substitution (**6a**) and introducing a chlorine atom (R^1^ = 3-F-4-Cl, **6m**), which also showed significantly lower inhibitory activity against K562; (2) when the R^2^ substituent contained a fluorine atom, the compound was significantly more active than other compounds with the same R^1^, for example, **6o** (R^2^ = 4-CF_3_; IC_50_ = 5.15 µM) > **6m** (R^2^ = 3,4-diCl; IC_50_ = 17.91 µM), **6n** (R^2^ = 4-OCH_3_, IC_50_ = 13.33 µM), and **6p** (R^2^ = 2-Cl; IC_50_ > 50 µM); and **6q** (R^2^ = 2,4-diF; IC_50_ = 5.61 µM) > **6r** (R^2^ = 4-Cl, IC_50_ = 10.03 µM), **6s** (R^2^ = 4-Br, IC_50_ = 10.78 µM), **6t** (R^2^ = 4-CN, IC_50_ = 11.08 µM), and **6u** (R^2^ = 4-pyridinyl; IC_50_ > 50 µM). This may be explained by the fact that the fluorine atom improved the permeability of the molecule by modulating its lipophilicity or directly interacting with the target protein [30,31,32,33]. Subsequently, the safety and selectivity of the most active compound **6o** were evaluated (Table 3), and the result showed that compound **6o** had a low cytotoxicity against HEK-293 cells with an IC_50_ value of 33.20 µM, and its selectivity was much higher (SI = 6.45) than **5-Fu** (SI = 0.14). Given its superior antitumor activity and safety profile, compound **6o** was selected for the preliminary mechanistic investigation.

### 2.3. Cell Apoptosis Analysis 

**Figure 3:**
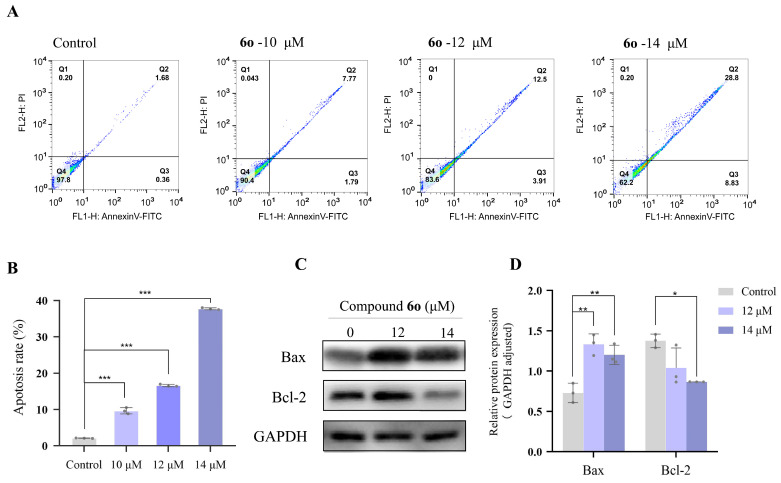
(**A**) Apoptosis detection assay (PI and AnnexinV-FITC staining); the test concentrations were 10, 12 and 14 μM. Q1 means necrosis cell, Q2 means late apoptosis, Q3 means early apoptosis, and Q4 means live cell. (**B**) Statistical chart of Apoptosis detection assay, statistic showed that **6o** caused apoptosis rate of K562 cells. (**C**) Detection of related apoptosis proteins Bax and Bcl-2 expression by Western blotting assay. GAPDH was used as a loading control. (**D**) Statistical chart of relative protein expression of Western blotting assay. Data represent the mean ± S.D of three independent experiments. * for *p* < 0.05, ** for *p* < 0.01, *** for *p* < 0.001.

To quantify the effects of **6o** on K562 cell apoptosis, K562 cells were treated with different concentrations of the compound **6o** and followed by Annexin V-FITC/PI detection assay. As shown in Figure 3A, when K562 cells were treated with 10, 12, and 14 μM of compound **6o** for 48 h, the total apoptosis rates (including early and late apoptosis) were 9.64%, 16.59%, and 37.72%, respectively, both showed an upward trend compared with the negative control. In conclusion, **6o** induced apoptosis of K562 cells in a dose-dependent manner, and the effect of late apoptosis was obvious. Meanwhile, the results of the Western blotting assay showed that **6o** could significantly affect the expression of apoptosis-related proteins, which decreased the expression of Bcl-2 (apoptosis-inhibiting protein) and increased the expression of Bax (pro-apoptotic protein) (Figure 3C,D). 

### 2.4. Cell Cycle Analysis

**Figure 4:**
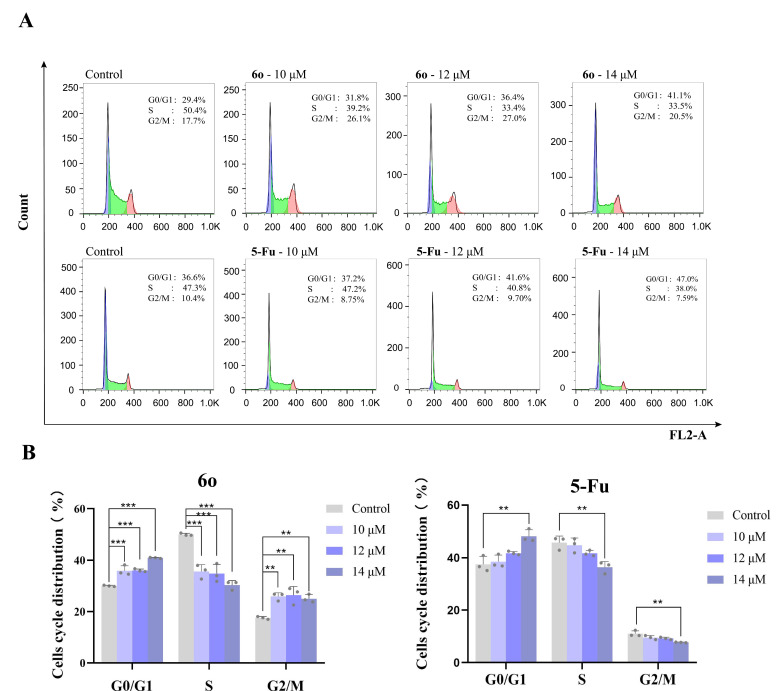
(**A**) Cell cycle detection assay (PI staining), the test concentrations were 10, 12 and 14 μM, blue represents G0/G1 phase, green represents S phase, and red represents G2/M phase. (**B**) Statistical chart of Cell cycle detection assay. Data represent the mean ± S.D of three independent experiments. ** for *p* < 0.01, *** for *p* < 0.001.

To further explore the antitumor properties of **6o**, the effect of **6o** on cell cycle distribution was evaluated. PI staining was used to perform cell cycle analysis. As shown in Figure 4, after treatment of K562 cells with **6o** (10, 12 and 14 μM) for 24 h, the G0/G1 population increased to 31.8%, 36.4%, and 41.1% from 29.4% (negative control), and the proportion of S phase cells decreased significantly. For the positive control- **5-Fu**, the proportion of the G0/G1 phase also increased by 10% after drug treatment. These results revealed that **6o** exerts antitumor properties by affecting the cell cycle distribution.

### 2.5. The Effect of Compound **6o** on the Expression of P53 and MDM2 Protein

**Figure 5:**
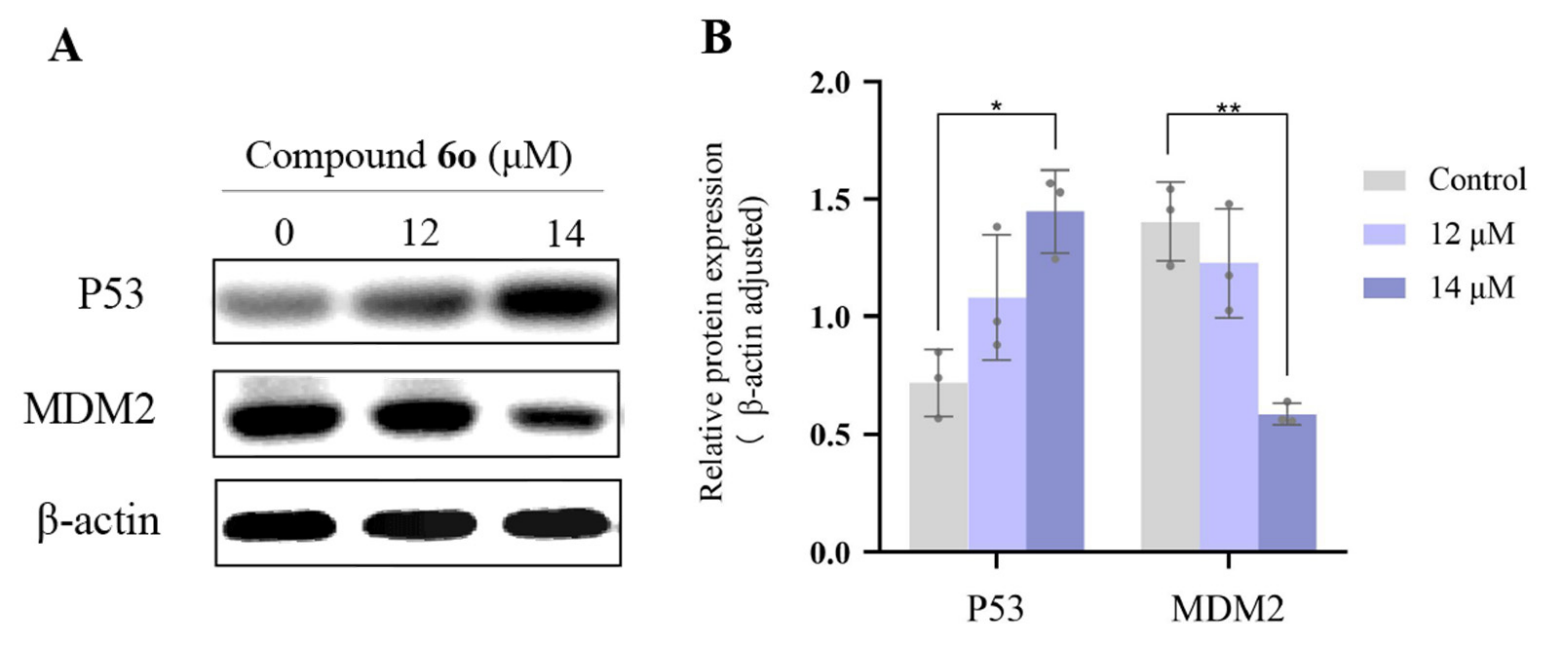
(**A**) Total cell lysate was analyzed by Western blotting assay for the expression of p53 and MDM2. (**B**) Statistical chart of relative protein expression of Western blotting assay. Protein expression levels were measured in arbitrary densitometry units, and data shows the mean value ± SEM calculated from relative protein expression levels determined in three separate experiments. * for *p* < 0.05, ** for *p* < 0.01.

Numerous studies have revealed that the p53 protein plays an essential role in a series of life activities such as DNA damage repair, cell cycle arrest, metabolism, senescence and apoptosis [34,35]. p53 protein accumulated in normal cells activates the expression of MDM2 protein in the downstream signaling pathway, and MDM2 binds to the transcriptional activation domain of p53, forming a p53-MDM2 complex, which inhibits the transcriptional activity of p53, and the whole process forms negative feedback regulates the pathway, thereby, achieving a stable balance of intracellular p53 levels. Loss of p53 activity by deletion, mutation, or MDM2 over-expression, is the most common event in the development and progression of cancer [36,37,38,39,40,41]. To confirm the ability of **6o** to inhibit the p53 and MDM2 interaction in K562, the expression levels of these two proteins were evaluated by Western blotting assay after 48 h of **6o** treatment. In these conditions, we found that **6o** could up-regulate the expression level of p53 protein and reduce the expression of MDM2 protein (Figure 5). In summary, **6o** could change the expression of p53 and MDM2, disrupting the balance of intracellular p53 protein levels, and eventually inducing apoptosis of K562.

## 3. Materials and Methods

### 3.1. Chemistry and Instruments

The reagents were purchased from commercial sources. All solvents and reagents were reagent-grade without further purification. The NMR data were obtained by Bruker Ascend 400 and 500 MHz (Bruker Optics, Billerica, MA, USA). High-resolution mass spectrometry (HRMS) spectra were conducted using a Thermo Scientific Q Exactive (Thermo Scientific, St. Louis, MO, USA). Melting points (mp.) for all target indazole derivatives were detected using a (X-4D, Cewei Optoelectronic Technology Co., Ltd., Shanghai, China) digital micro melting point apparatus.

### 3.2. Pharmacology

#### 3.2.1. Cell Culture and Treatment

Lung (A549), chronic myeloid leukemia (K562), prostate (PC3), and live (Hep-G2) cells were purchased from the Cell Bank of the Chinese Academy of Sciences (Kunming, China). A549, K562, and PC-3 cells were cultured in RPMI-1640 medium and the liver cells Hep-G2 were cultured in MEM medium. All mediums were supplemented with 10% FBS, 100 μg/mL penicillin and 100 μg/mL streptomycin. All cells were cultivated in a 5% CO_2_ incubator at 37 °C.

#### 3.2.2. MTT Assay

To investigate the anticancer activity of indazole derivatives, A549, K562, PC3, and Hep-G2 cells were treated with targets compounds respectively for 48 h in a range of concentrations (from 0.625 to 10 μM) and subjected to a proliferation assay using the MTT assay. Exponentially growing cells were seeded at a density of 5000 cells per well in 96-well plates containing 100 μL of fresh growth medium. After allowing the cells to attach for 24 h, target compounds were added. After incubation for 48 h, viable cells were stained with MTT (0.5 mg/mL) for 4 h, and the produced formazan crystals were dissolved with 150 µL of dimethyl sulfoxide. Absorbance was measured at 490 nm using an automated microplate reader (Tecan, Infinite M200 Pro, Männedorf, Switzerland). The relative cell viability was calculated according to the following equation:

$$\mathrm{Cell}\;\mathrm{viability}\left(\%\right)=\frac{\mathrm{experimental}\;\mathrm{OD}\;\mathrm{value}}{\mathrm{control}\;\mathrm{OD}\;\mathrm{value}}\times 100\%\mathrm{Cell}\;\mathrm{inhibition}\left(\%\right)=\frac{\mathrm{control}\;\mathrm{OD}\;\mathrm{value}-\mathrm{experimental}\;\mathrm{OD}\;\mathrm{value}}{\mathrm{control}\;\mathrm{OD}\;\mathrm{value}}\times 100\%$$

$$\mathrm{Cell}\;\mathrm{inhibition}\left(\%\right)=\frac{\mathrm{control}\;\mathrm{OD}\;\mathrm{value}-\mathrm{experimental}\;\mathrm{OD}\;\mathrm{value}}{\mathrm{control}\;\mathrm{OD}\;\mathrm{value}}\times 100\%$$

#### 3.2.3. Cell Apoptosis Detection Assay

Activation of tumor cell apoptosis plays an important role in treatment cancer. To investigate whether apoptosis could underlie the anticancer activity of **6o**, K562 cells were treated with **6o** (0, 10, 12 or 14 μM) for 48 h and the percentage of apoptosis was determined by flow cytometry, Annexin V-FITC/PI Apoptosis Detection Kit (BD, Franklin Lakes, NJ, USA) was used. Cells (1 × 10^6^/mL per well) were plated in 6-well culture plates and grown for 24 h. After treatment with compound **6o** for 48 h, the collected cells were washed two times with ice-cold PBS, counted, and 100 μL of cell suspension with a density of 1 × 10^6^ cells/mL was prepared, then incubated with 5 μL Annexin V-FITC and 5 μL Propidium iodide (PI) in the dark for 15 min. After 15 min of incubation and added 400 μL of 1 × binding buffer, and use Cell Quest software (BD, SN: E34297502014) to perform detection on Flow Cytometry (BD, Franklin Lakes, NJ, USA). Data were analyzed using FlowJo V10.

#### 3.2.4. Cell Cycle Analysis

To analyze the cell cycle, a PI apoptosis detection kit (Solarbio, Wuhan, China) was utilized. After K562 cells were seeded in 6-well plates for 24 h, compound **6o** (10, 12 and 14 μM) or positive control was added to the plates for another 24 h. Then, cells were washed three times with PBS, and fixed with 70% ethanol at 4°C for overnight. After fixed, cells were again washed with PBS and incubated at 37 °C with 100 μL RNase A for 30 min. After this, cells were incubated with 400 μL PI for 30 min at 4 °C. After staining, cells were subjected to Flow Cytometry using Cell Quest software (BD, SN: E34297502014) and the PI fluorescence was recorded in the FL2 channel. Data were analyzed using FlowJo V10.

#### 3.2.5. Western Blotting Assay

K562 cells were treated with compound **6o** for 48 h, then, lysed in 1×RIPA lysis buffer (Solarbio) with proteinase (Solarbio) and phosphatase inhibitors (Solarbio) for 15 min on ice (proteinase: phosphatase inhibitors: 1 × RIPA lysis buffer = 1:1:100), scraped off from the plate and prepared cell lysates. These lysates were centrifuged at 1.2 × 10^4^ rpm at 4 °C for 15 min to collect the supernatants and the protein concentration of each sample was quantified by the BCA protein assay kit (Beyotime, Shanghai, China). Protein was separated by SDS-PAGE and then transferred onto the PVDF membrane. After blocking with 5% skim milk for 2 h, befitting primary antibodies include Bax (Proteintech; Type: Rabbit; Cat No.: 50599-2-lg); Bcl-2 (Proteintech; Type: Mouse; Cat No.: 60178-1-lg); GAPDH (Proteintech; Type: Mouse; Cat No.: 60004-1-lg); β-actin (Proteintech; Type: Rabbit; Cat No.: 81115-1-RR); P53 (Cell signaling; Type: Rabbit; Cat No.: #2527) and MDM2 (Cell signaling; Type: Rabbit; Cat No.: #86934) were sealed in Antibody Dilution Buffer, and cocultivated with the PDF membrane at 4 °C for overnight. After this, the second antibody (Cell signaling, Type: Mouse and Ribbit; Cat No.: #7076 and #7074) was replenished and cofostered with the above membrane for an additional 1 h at room temperature. Finally, bands were visualized containing an enhanced chemiluminescence system (Millipore, Burlington, MA, USA).

#### 3.2.6. Statistical Analysis

Statistical processing of all the results was performed using GraphPad Prism 8.0 software. All data were expressed as mean ± SD and all the data provided have been verified by at least three independent experiments. All data statistical differences were performed with Student’s *t*-test or one-way ANOVA using GraphPad Prism 8. Differences among groups were considered significant at *p* ≤ 0.05.

## 4. Conclusions

In conclusion, thirty-eight 1*H*-indazole-3-amine derivatives were designed, synthesized, and evaluated for their cytotoxic potential against K562, A549, PC-3, and Hep-G2 cell lines in vitro. The preliminary results suggested that most of the target compounds showed great antitumor activity. Among these, mercapto acetamide-derived compound **5k** showed the best inhibitory effect against Hep-G2 (IC_50_ = 3.32 µM), however, this compound showed high toxicity to normal cells. Then, mercapto was replaced with piperazine, in piperazine derivatives, the most potent compound **6o** exhibited a broad spectrum of activity against all the test cancer cell lines and specifically for K562 cells with an IC_50_ value of 5.15 ± 0.55 μM. Also, it showed 6.45-fold selectivity towards the K562 cancer cell line compared to the HEK-293. Compound **6o** induced the apoptosis of K562 cells in a dose-dependent manner via and arrested the cell cycle in G0/G1 phase. Under normal circumstances, the expression level of p53 protein in cells is low and exhibits dynamic balance. After drug stimulation, the phosphorylated p53 dissociates from the MDM2 complex, resulting in an increase in p53 protein levels. And the sustained high level of p53 leads to the transcription of apoptosis-related genes such as Bax, which then leads to cell apoptosis and inhibits the occurrence of tumors. In this study, Western blotting results indicated that this compound could reduce the expression of MDM2 protein and up-regulate the expression level of p53 protein. Besides, it also could increase the level of the pro-apoptotic protein Bax and reduce the anti-apoptotic protein Bcl-2. The above research suggests that **6o** may be a potential target molecule for p53-MDM2. Further research is still needed to elucidate the precise target or mechanism of anti-cancer activity of **6o**, which enables extensive optimization of this compound.

## Figures and Tables

**Table 1:** IC_50_ values of compound **5a**–**5q** against four tumor cell lines in vitro.

Compounds	IC_50_ ± SD ^a^ (μM)			
	K562	A549	PC-3	Hep-G2
**5a**	9.32 ± 0.59	4.66 ± 0.45	15.48 ± 1.33	12.67 ± 1.31
**5b**	6.97 ± 0.99	5.25 ± 0.57	38.43 ± 24.12	>50
**5c**	9.33 ± 0.64	5.63 ± 0.59	60.74 ± 2.91	4.76 ± 0.63
**5d**	27.23 ± 0.66	16.64 ± 3.22	19.67 ± 2.38	45.90 ± 2.24
**5e**	>50	7.23 ± 0.40	12.83 ± 3.09	8.20 ± 0.91
**5f**	>50	4.48 ± 0.24	12.46 ± 4.55	>50
**5g**	10.31 ± 1.14	6.40 ± 0.26	18.62 ± 5.73	11.87 ± 0.72
**5h**	14.35 ± 2.13	9.26 ± 0.37	13.02 ± 7.53	11.28 ± 0.75
**5i**	6.27 ± 1.22	4.43 ± 0.15	6.57 ± 1.10	5.50 ± 0.17
**5j**	8.49 ± 1.87	3.99 ± 0.16	14.72 ± 4.55	5.36 ± 0.25
**5k**	8.86 ± 0.66	7.78 ± 0.18	12.62 ± 2.17	3.32 ± 0.43
**5l**	8.19 ± 1.43	14.06 ± 1.71	>50	14.92 ± 0.77
**5m**	13.13 ± 0.46	20.21 ± 3.14	>50	35.64 ± 4.21
**5n**	7.25 ± 1.07	20.00 ± 1.19	>50	6.57 ± 0.13
**5o**	8.09 ± 1.27	6.64 ± 0.47	12.51 ± 2.10	8.30 ± 0.56
**5p**	15.42 ± 0.15	15.95 ± 0.37	22.51 ± 9.13	7.14 ± 0.33
**5q**	14.35 ± 2.13	16.25 ± 1.46	7.81 ± 0.64	6.30 ± 0.03
5-Fu ^b^	8.53 ± 2.27	8.37 ± 0.79	9.17 ± 3.93	9.78 ± 1.58

^a^ IC_50_ values are the concentrations that cause 50% inhibition of cancer cell growth. Data represent the mean values ± standard deviation of three independent experiments performed in triplicate. ^b^ 5-Fluorouracil (5-Fu) as the reference standard.

**Table 2:** IC_50_ values of compound **6a**–**6u** against four tumor cell lines in vitro.

Compounds	IC_50_ ± SD (μM)			
	K562	A549	PC-3	Hep-G2
**6a**	5.19 ± 0.29	8.21 ± 0.56	6.12 ± 0.10	5.62 ± 1.76
**6b**	18.62 ± 2.14	19.90 ± 1.15	9.69 ± 0.26	1.69 ± 0.68
**6c**	>50	24.03 ± 2.92	>50	1.23 ± 0.39
**6d**	18.23 ± 2.91	17.55 ± 4.11	17.58 ± 1.40	>50
**6e**	8.85 ± 0.26	12.78 ± 0.78	7.15 ± 0.14	>50
**6f**	11.91 ± 0.85	>50	41.74 ± 27.89	9.56 ± 0.84
**6g**	6.61 ± 0.38	15.85 ± 2.98	6.81 ± 1.09	2.35 ± 0.96
**6h**	13.23 ± 1.75	15.13 ± 1.36	8.63 ± 0.57	2.75 ± 0.78
**6i**	13.23 ± 1.44	28.58 ± 2.52	12.81 ± 2.95	2.58 ± 0.42
**6j**	7.78 ± 0.10	20.29 ± 1.24	7.58 ± 1.01	3.87 ± 0.47
**6k**	11.18 ± 0.56	7.83 ± 1.06	4.25 ± 0.13	1.61 ± 0.28
**6l**	14.01 ± 0.49	12.90 ± 0.21	2.89 ± 0.16	7.65 ± 3.34
**6m**	17.91 ± 3.14	22.62 ± 6.53	19.94 ± 2.71	1.51 ± 0.25
**6n**	13.33 ± 1.40	18.66 ± 3.10	34.84 ± 4.37	4.76 ± 0.63
**6o**	5.15 ± 0.55	6.73 ± 1.03	8.34 ± 1.55	7.37 ± 0.24
**6p**	>50	13.55 ± 0.53	32.10 ± 2.26	2.38 ± 0.37
**6q**	5.61 ± 1.14	12.85 ± 2.66	10.74 ± 1.66	2.70 ± 0.47
**6r**	10.03 ± 0.94	15.85 ± 1.38	27.89 ± 26.14	>50
**6s**	10.78 ± 0.68	20.40 ± 8.08	20.26 ± 4.71	2.92 ± 1.41
**6t**	11.08 ± 0.69	40.88 ± 6.54	18.97 ± 0.58	1.98 ± 0.35
**6u**	>50	17.55 ± 4.11	27.66 ± 16.92	20.04 ± 3.04
**5-Fu**	8.53 ± 2.27	8.37 ± 0.79	3.47 ± 0.21	2.25 ± 0.46

**Table 3:** Selectivity index (SI) of compound **5k** and **6o** for Hep-G2 and K562 cells.

Compounds	IC_50_ ± SD (μM)			SI ^a^
	Hep-G2	K562	HEK-293	
**5k**	3.32 ± 0.43		12.17 ± 2.85	3.67
**6o**		5.15 ± 0.55	33.20 ± 3.83	6.45
**5-Fu**	9.78 ± 1.58	8.53 ± 2.27	1.16 ± 0.11	0.12 ^b^; 0.14 ^c^

^a^ The selectivity indexes (SI) were calculated by IC_50_ values in HEK-293 divided by IC_50_ value in cancer cell lines. ^b^ Selectivity index (SI) of compound **5-Fu** for Hep-G2 cells. ^c^ Selectivity index (SI) of compound **5-Fu** for K562 cells.

## Data Availability

Not applicable.

## Supplementary Materials

The following supporting information can be downloaded at: https://www.mdpi.com/article/10.3390/ijms24108686/s1.
