# Supplementary material for: Design, Synthesis and Antitumor Activity of 1H-indazole-3-amine Derivatives
Source: Int J Mol Sci. 2023 May 12;24(10):8686. doi: 10.3390/ijms24108686 (PMC10218234; doi:10.3390/ijms24108686)
Supplement: Supplementary file 1 [file ijms-24-08686-s001.zip › ijms-2360215-supplementary.pdf]

Design, Synthesis and Antitumor Activity of 1*H*-indazole-3-amineDerivatives

CongyuWang [1,3,†], Mei Zhu [2,†], Xuesha Long [1,3], Qin Wang[1,3], Zhenchao Wang and Guiping Ouyang [1,2,3,*]

1 College of Pharmacy, Guizhou University, Guiyang 550025, China;
2 Center for Research and Development of Fine Chemicals, Guizhou University, Guiyang 550025, China;
3 Guizhou Engineering Laboratory for Synthetic Drugs, Guizhou University, Guiyang, 550025, China.
* Correspondence: gpouyang@gzu.edu.cn (GP.OY.), Tel: +86-851-8830-8270, Fax: +86-851-8830-8270
† These authors contributed equally to this manuscript.

Content

1. General preparation for preparing intermediates 2......................2

2. General preparation for preparing intermediates 3......................2

3. General preparation for preparing intermediates 4......................2

4. Synthesis of the target compounds 5a-5q 3

5. Synthesis of the target compounds 6a-6u 3

6. The data of title compounds. 3

7. ^{1}H NMR, ^{13}C NMR, and HRMS spectra for target compounds. 19

8. The graphs for IC_{50} of compound 6o in K562. 76

1. General preparation for preparing intermediates 2.

Compound **1** (1 mol) was added sequentially in a 50 mL round bottom flask, followed by 80% hydrazine hydrate (3 mol), dissolved in an appropriate amount of anhydrous ethanol, heated to reflux for 2 h. The reaction solution was cooled to room temperature and poured into ice water in batches, at the same time, yellow precipitates appeared. Continue stirring at room temperature for 30 min and filter.

2. General preparation for preparing intermediates 3.

In a round-bottom flask, compound **2** (1 mol), anhydrous cesium carbonate (2.5 mol), and each substituted phenylboronic acid (1.5 mol) was added sequentially. Subsequently, an appropriate amount of a mixture of 1,4-dioxane and ultrapure water (1:1) was added and stirred at room temperature until the compound was completely dissolved, and then $PdCl_2(dppf)_2$ (0.1 mol) was added. The mixture was placed in an oil bath and heated to 90℃ for 5 h under protecting by nitrogen. After the reaction solution was cooled to room temperature, The solvent was evaporated under reduced pressure and the residue was purified by flash column chromatography on silica gel using (EA: PE = 1: 2 v/v) to afford the compound as pure yellow solid.

3. General preparation for preparing intermediates 4

In a round bottom flask, add compound **3** (1 mol), Na_2CO_3 (1.6 mol), and appropriate amount of 1,4-dioxane; subsequently, dilute chloroacetic

anhydride (1 mol) with an appropriate amount of 1,4-dioxane, add it drop by drop to the above mixture, stir at room temperature. When the reaction of raw materials is complete, add an appropriate amount of ultrapure water, NaCl, and crushed ice in a beaker, stir well, then pour the reaction solution into the beaker and filtration, the crude mixture was chromatography over silica gel (EA: PE = 1: 2 v/v).

4. Synthesis of the target compounds 5a-5q

Compound **4** (1 mol) and the differently substituted thiophenols (1 moL) were dissolved in an appropriate amount of anhydrous ethanol, and then KOH (1 mol) was added, and the reaction solution was stirred at room temperature with overnight. The reaction was measured by TLC and waited until the reaction of the raw materials was complete. Subsequently, the reaction solution was slowly poured in ice water at 0°C, at which time the precipitate precipitated, stirred at room temperature until the ice was completely melted, extracted and filtered, and post-treated by recrystallization.

5. Synthesis of the target compounds 6a-6u

Compound 4 (1 moL) and differently substituted piperazine (1.1 moL) were added to an appropriate amount of acetonitrile, then K_2CO_3 (1.1 moL) was added to the mixture, then, reflux for 5-10 h. The TLC plate was tracked until the reaction was complete, The solvent was evaporated under reduced pressure and the residue was purified anhydrous ethanol.

6. The data of title compounds.

***N-(5-(3-fluorophenyl)-1H-indazol-3-yl)-2-((4-methoxyphenyl)thio)acetamide* *(5a)*.** mp: 181-182℃; Yield: 75%; ^{1}H NMR (400 MHz, DMSO-d_6) *δ:* 12.82 (s, 1H), 10.64 (s, 1H), 7.86 (s, 1H), 7.68 (d, *J* = 8.2 Hz, 1H), 7.59 – 7.29 (m, 6H), 7.19 (td, *J* = 8.7, 8.3, 2.5 Hz, 1H), 6.91 (d, *J* = 8.7 Hz, 2H), 3.83 (s, 2H), 3.68 (d, *J* = 2.4 Hz, 3H). ^{13}C NMR (101 MHz, DMSO-d_6) *δ* 167.83, 159.19, 141.14, 141.00, 133.01, 131.35, 131.26, 131.04, 126.42, 125.76, 123.20, 123.18, 120.76, 117.08, 115.15, 114.03, 113.82, 113.61, 111.26, 55.58, 39.04. HRMS (AP-ESI) m/z clad for [$C_{22}H_{18}FN_3O_2S$] [M + H]$^+$ 408.1177; found, 408.1176.

***N-(5-(3-fluorophenyl)-1H-indazol-3-yl)-2-((4-fluorophenyl)thio)acetamide* *(5b)*.** mp: 170-172℃; Yield: 68%; ^{1}H NMR (400 MHz, DMSO-*d6*) *δ:* 12.80 (s, 1H), 10.67 (s, 1H), 7.85 (s, 1H), 7.68 (dd, *J* = 8.7, 1.8 Hz, 1H), 7.63 – 7.30 (m, 6H), 7.19 (td, *J* = 8.3, 4.8 Hz, 3H), 3.95 (s, 2H). ^{13}C NMR (101 MHz, DMSO-d_6) *δ:* 167.56, 141.13, 140.88, 132.00, 131.92, 131.34, 131.26, 126.44, 123.16, 120.62, 116.65, 116.44, 114.05, 113.81, 113.59, 111.30, 37.85. HRMS (AP-ESI) m/z clad for [$C_{21}H_{15}F_2N_3OS$] [M + H]$^+$ 396.0977; found, 396.0977.

***2-((4-Bromophenyl)thio)-N-(5-(3-fluorophenyl)-1H-indazol-3-yl)acetamide* *(5c)*.** mp: 170-171℃; Yield: 62%; ^{1}H NMR (400 MHz, DMSO-d_6) *δ:* 12.83 (s, 1H), 10.75 (s, 1H), 7.85 (s, 1H), 7.68 (dd, *J* = 8.8, 1.8 Hz, 1H), 7.53 (dt, *J* = 6.6, 3.3 Hz, 4H), 7.46 – 7.35 (m, 4H), 7.18 (td, *J* = 8.7, 2.5 Hz, 1H), 4.01 (s, 2H). ^{13}C NMR (101 MHz, DMSO-d_6) *δ:* 167.35, 164.38, 141.14, 140.85, 135.91, 132.26, 131.35, 131.27, 131.12, 130.62, 126.45, 123.15, 123.13, 120.60, 119.50, 117.06, 114.04, 113.83, 113.61, 111.31, 36.78. HRMS (AP-ESI) m/z clad for [$C_{21}H_{15}BrFN_3OS$] [M + H]$^+$

456.0176; found, 456.0176.

2-((2-Bromophenyl)thio)-N-(5-(3-fluorophenyl)-1H-indazol-3-yl)acetamide (5d). mp: 169-171℃; Yield: 67%; ^{1}H NMR (400 MHz, DMSO-*d*$_6$) *δ:* 12.84 (s, 1H), 10.83 (s, 1H), 7.89 (s, 1H), 7.72 – 7.60 (m, 2H), 7.52 (ddd, *J* = 20.6, 8.0, 5.5 Hz, 3H), 7.46 – 7.32 (m, 3H), 7.20 – 7.09 (m, 2H), 4.08 (s, 2H). ^{13}C NMR (101 MHz, DMSO-*d*$_6$) *δ* 167.03, 164.36, 161.95, 141.15, 140.83, 137.80, 133.21, 131.26, 128.76, 127.83, 127.35, 126.45, 123.19, 121.66, 120.59, 117.08, 113.59, 111.33, 36.19. HRMS (AP-ESI) m/z clad for [$C_{21}H_{15}BrFN_3OS$] [M + H]$^+$ 456.0176; found, 456.0176.

N-(5-(4-fluorophenyl)-1H-indazol-3-yl)-2-((4-methoxyphenyl)thio)acetamide (5e). mp: 199-201℃; Yield: 77%; ^{1}H NMR (400 MHz, DMSO-*d*$_6$) *δ:* 12.76 (s, 1H), 10.59 (s, 1H), 7.78 (d, *J* = 1.6 Hz, 1H), 7.61 (ddd, *J* = 8.8, 5.2, 3.6 Hz, 3H), 7.52 (dd, *J* = 8.7, 0.8 Hz, 1H), 7.49 – 7.43 (m, 2H), 7.34 – 7.27 (m, 2H), 6.95 – 6.86 (m, 2H), 3.83 (s, 2H), 3.69 (s, 3H). ^{13}C NMR (101 MHz, DMSO-*d*$_6$) *δ:* 167.79, 159.14, 140.88, 137.81, 132.92, 131.47, 129.08, 129.00, 126.46, 125.76, 120.33, 117.12, 116.25, 116.03, 115.17, 111.18, 55.60, 38.96. HRMS (AP-ESI) m/z clad for [$C_{22}H_{18}FN_3O_2S$] [M + H]$^+$ 408.1177; found, 408.1176.

2-((4-Methoxyphenyl)thio)-N-(5-(4-(trifluoromethoxy)phenyl)-1H-indazol-3-yl)acetamide (5f). mp: 215-216℃; Yield: 74%; ^{1}H NMR (400 MHz, DMSO-*d*$_6$) *δ:* 12.78 (s, 1H), 10.59 (s, 1H), 7.99 – 7.19 (m, 9H), 6.91 (d, *J* = 8.3 Hz, 2H), 3.83 (s, 2H), 3.68 (s, 3H). ^{13}C NMR (101 MHz, DMSO-*d*$_6$) *δ* 167.81, 159.14, 147.86, 141.06, 140.97, 140.65, 132.95, 130.99, 128.92, 126.44, 125.74, 121.95, 120.75, 117.11, 115.16, 111.31, 55.55, 38.95. HRMS (AP-ESI) m/z clad for [$C_{23}H_{18}F_3N_3O_3S$] [M +

H]$^+$ 474.1094; found, 474.1093.

N-(5-(4-Chloro-3-fluorophenyl)-1H-indazol-3-yl)-2-(4-fluorophenyl)thio)acetamide (5g). mp: 178-200℃; Yield: 62%; ^{1}H NMR (400 MHz, DMSO-d_6) δ: 12.78 (s, 1H), 10.59 (s, 1H), 7.99 – 7.19 (m, 9H), 6.91 (d, J = 8.3 Hz, 2H), 3.83 (s, 2H), 3.68 (s, 3H). ^{13}C NMR (101 MHz, DMSO-d_6) δ: 167.55, 162.76, 159.26, 156.81, 141.17, 140.93, 131.99, 131.91, 131.43, 126.25, 124.15, 120.72, 118.31, 118.13, 117.02, 116.64, 116.42, 115.28, 115.06, 111.41, 37.84. HRMS (AP-ESI) m/z clad for [$C_{21}H_{14}ClF_2N_3OS$] [M + H]$^+$ 430.0587; found, 430.0587.

2-((4-Bromophenyl)thio)-N-(5-(4-chloro-3-fluorophenyl)-1H-indazol-3-yl)acetamide (5h). mp: 267-268℃; Yield: 70%; ^{1}H NMR (400 MHz, DMSO-d_6) δ: 12.81 (s, 1H), 10.75 (s, 1H), 7.93 – 7.77 (m, 1H), 7.74 – 7.39 (m, 9H), 4.01 (s, 2H). ^{13}C NMR (101 MHz, DMSO-d_6) δ: 167.37, 159.26, 142.51, 141.20, 140.91, 135.88, 132.26, 131.46, 130.62, 130.07, 126.32, 124.14, 120.70, 119.49, 117.03, 115.31, 111.42, 36.74. HRMS (AP-ESI) m/z clad for [$C_{21}H_{14}BrClFN_3OS$] [M + H]$^+$ 489.9786; found, 489.9786.

N-(5-(4-chloro-3-fluorophenyl)-1H-indazol-3-yl)-2-((4-nitrophenyl)thio)acetamide (5i). mp: 243-246℃; Yield: 75%; ^{1}H NMR (400 MHz, DMSO-d_6) δ: 12.88 (s, 1H), 10.90 (s, 1H), 8.18 (d, J = 9.0 Hz, 2H), 7.88 (s, 1H), 7.71 - 7.52 (m, 6H), 7.41 (dd, J = 8.4, 2.1 Hz, 1H), 4.22 (s, 2H). ^{13}C NMR (101 MHz, DMSO-d_6) δ: 166.75, 159.23, 147.29, 145.18, 141.21, 140.83, 131.39, 130.07, 127.03, 126.34, 124.37, 124.10, 124.07, 120.68, 116.99, 115.25, 115.03, 111.44, 35.58. HRMS (AP-ESI) m/z clad for [$C_{21}H_{14}ClFN_4O_3S$] [M + H]$^+$ 455.0375; found, 455.0389.

***N*-(5-(3,5-difluorophenyl)-1*H*-indazol-3-yl)-2-((4-methoxyphenyl)thio)acetamide (5j).** mp: 204-205℃; Yield: 73%; ^{1}H NMR (400 MHz, DMSO-d_6) *δ:* 12.92 (s, 1H), 10.72 (s, 1H), 7.95 (s, 1H), 7.70 (d, *J* = 8.8 Hz, 1H), 7.64 – 7.08 (m, 6H), 6.90 (d, *J* = 8.5 Hz, 2H), 3.86 (s, 2H), 3.69 (s, 3H). ^{13}C NMR (101 MHz, DMSO-d_6) *δ:* 167.36, 164.62, 162.04, 141.32, 140.98, 135.90, 132.24, 130.66, 129.98, 126.35, 120.95, 119.52, 116.98, 111.39, 110.19, 109.93, 102.78, 102.52, 36.82. HRMS (AP-ESI) m/z clad for [$C_{22}H_{17}F_2N_3O_2S$] [M + H]$^+$ 426.1082; found, 426.1082.

2-((4-Bromophenyl)thio)-N-(5-(3,5-difluorophenyl)-1H-indazol-3-yl)acetamide (5k). mp: 204-205℃; Yield: 70%; ^{1}H NMR (400 MHz, DMSO-d_6) *δ:* 12.92 (s, 1H), 10.72 (s, 1H), 7.95 (s, 1H), 7.70 (d, *J* = 8.8 Hz, 1H), 7.64 – 7.08 (m, 6H), 6.90 (d, *J* = 8.5 Hz, 2H), 3.86 (s, 2H), 3.69 (s, 3H). ^{13}C NMR (101 MHz, DMSO-d_6) *δ:* 167.84, 164.62, 159.16, 145.05, 141.29, 141.07, 132.96, 129.83, 126.25, 125.80, 121.05, 116.96, 115.12, 111.37, 110.18, 109.92, 102.72, 102.46, 102.21, 55.57. HRMS (AP-ESI) m/z clad for [$C_{21}H_{14}BrF_2N_3OS$] [M + H]$^+$ 474.0082; found, 474.0082.

2-((2-Bromophenyl)thio)-N-(5-(3,5-difluorophenyl)-1H-indazol-3-yl)acetamide (5l). mp: 199-200℃; Yield: 73%; ^{1}H NMR (400 MHz, DMSO-d_6) *δ:* 12.88 (s, 1H), 10.84 (s, 1H), 7.92 (s, 1H), 7.71 (dd, *J* = 8.8, 1.8 Hz, 1H), 7.64 (dd, *J* = 8.0, 1.4 Hz, 1H), 7.59 - 7.51 (m, 2H), 7.42 (td, *J* = 7.7, 1.4 Hz, 1H), 7.35 – 7.25 (m, 2H), 7.20 (tt, *J* = 9.3, 2.3 Hz, 1H), 7.12 (td, *J* = 7.7, 1.5 Hz, 1H), 4.09 (s, 2H). ^{13}C NMR (101 MHz, DMSO-d_6) δ 167.05, 164.61, 164.47, 162.17, 162.03, 144.95, 141.34,

140.96, 137.79, 133.20, 129.98, 128.72, 127.87, 127.34, 126.34, 121.70, 120.92, 117.03, 111.41, 110.19, 109.94, 102.51, 36.21. HRMS (AP-ESI) m/z clad for $[C_{21}H_{14}BrF_2N_3OS]$ $[M + H]^+$ 474.0082; found, 474.0083.

2-((4-Chlorophenyl)thio)-N-(5-(3,5-difluorophenyl)-1H-indazol-3-yl)acetamide (5m). mp: 200-202℃; Yield: 65%; ^1H NMR (400 MHz, DMSO-d_6) *δ:* 12.87 (s, 1H), 10.76 (s, 1H), 7.89 (d, *J* = 1.8 Hz, 1H), 7.70 (dd, *J* = 8.7, 1.8 Hz, 1H), 7.57 – 7.45 (m, 3H), 7.42 – 7.36 (m, 2H), 7.33 – 7.26 (m, 2H), 7.21 (tt, *J* = 9.3, 2.3 Hz, 1H), 4.01 (s, 2H). ^{13}C NMR (101 MHz, DMSO-d_6) *δ:* 167.40, 164.62, 164.48, 162.18, 141.33, 140.99, 135.30, 131.29, 130.47, 129.98, 129.37, 126.35, 120.94, 116.98, 111.39, 110.18, 109.92, 102.51, 36.97. HRMS (AP-ESI) m/z clad for $[C_{21}H_{14}ClF_2N_3OS]$ $[M + H]^+$ 430.0587; found, 430.0587.

2-((3-Chlorophenyl)thio)-N-(5-(3,5-difluorophenyl)-1H-indazol-3-yl)acetamide (5n). mp: 193-194℃; Yield: 67%; ^1H NMR (400 MHz, DMSO-d_6) *δ:* 12.88 (s, 1H), 10.79 (s, 1H), 7.90 (d, *J* = 1.8 Hz, 1H), 7.71 (dd, *J* = 8.8, 1.8 Hz, 1H), 7.59 – 7.51 (m, 2H), 7.41 (d, *J* = 7.8 Hz, 1H), 7.35 (t, *J* = 7.8 Hz, 1H), 7.32 – 7.16 (m, 4H), 4.06 (s, 2H). ^{13}C NMR (101 MHz, DMSO-d_6) *δ:* 167.38, 162.16, 141.32, 140.92, 139.02, 134.14, 130.99, 130.03, 127.36, 126.84, 126.37, 126.24, 120.81, 117.04, 111.41, 110.19, 109.94, 102.52, 36.46. HRMS (AP-ESI) m/z clad for $[C_{21}H_{14}ClF_2N_3OS]$ $[M + H]^+$ 430.0587; found, 430.0587.

N-(5-(3,5-difluorophenyl)-1H-indazol-3-yl)-2-((4-nitrophenyl)thio)acetamide (5o). mp: 222-224℃; Yield: 77%; ^1H NMR (400 MHz, DMSO-d_6) *δ:* 12.88 (s, 1H), 10.88 (s, 1H), 8.18 (d, *J* = 8.6 Hz, 2H), 7.90 (s, 1H), 7.78 – 7.45 (m, 4H), 7.41 – 6.95

(m, 3H), 4.22 (s, 2H). ^{13}C NMR (101 MHz, DMSO-d_6) *δ:* 166.79, 162.16, 147.28, 145.18, 144.88, 141.33, 140.88, 129.97, 126.97, 126.36, 124.35, 120.84, 116.98, 111.44, 110.09, 109.83, 102.51, 35.54. HRMS (AP-ESI) m/z clad for [$C_{21}H_{14}F_2N_4O_3S$] [M - H]$^+$ 439.0671; found, 439.0683.

N-(5-(3,5-difluorophenyl)-1H-indazol-3-yl)-2-(pyridin-4-ylthio)acetamide (5p). mp: 250-251℃; Yield: 78%; ^{1}H NMR (400 MHz, DMSO-d_6) *δ:* 12.88 (s, 1H), 10.86 (s, 1H), 8.51 – 8.20 (m, 2H), 7.97 (s, 1H), 7.78 – 7.05 (m, 7H), 3.32 (s, 2H). ^{13}C NMR (101 MHz, DMSO-d_6) *δ* 166.88, 164.61, 164.48, 162.04, 149.68, 148.34, 144.96, 141.34, 140.92, 130.02, 126.37, 121.07, 116.96, 111.41, 110.18, 110.00, 109.92, 102.51, 102.26, 34.55. HRMS (AP-ESI) m/z clad for [$C_{20}H_{14}F_2N_4OS$] [M + H]$^+$ 397.0929; found, 397.0929.

N-(5-(3,5-difluorophenyl)-1H-indazol-3-yl)-2-(pyrimidin-2-ylthio)acetamide (5q). mp: 234-237℃; Yield: 65%; ^{1}H NMR (400 MHz, DMSO-d_6) *δ:* 12.83 (s, 1H), 10.74 (s, 1H), 8.68 (d, *J* = 4.9 Hz, 2H), 7.99 (s, 1H), 7.84 – 7.68 (m, 1H), 7.55 (d, *J* = 8.7 Hz, 1H), 7.45 – 7.01 (m, 4H), 4.22 (s, 2H). ^{13}C NMR (101 MHz, DMSO-d_6) *δ:* 170.99, 166.99, 164.60, 162.02, 158.26, 145.03, 141.32, 141.22, 129.91, 126.29, 121.07, 117.88, 117.15, 111.36, 110.19, 109.94, 102.47, 35.16. HRMS (AP-ESI) m/z clad for [$C_{19}H_{13}F_2N_5OS$] [M + H]$^+$ 398.0882; found, 398.0882.

2-(4-(3,4-dichlorophenyl)piperazin-1-yl)-N-(5-(3-fluorophenyl)-1H-indazol-3-yl)acetamide (6a). mp: 170-172℃; Yield: 52%; ^{1}H NMR (400 MHz, Chloroform-*d*) *δ:* 10.27 (s, 1H), 9.60 (s, 1H), 8.26 (s, 1H), 7.61 (d, *J* = 8.6 Hz, 1H), 7.37 (ddd, *J* = 47.8, 16.3, 9.5 Hz, 6H), 7.12 – 6.93 (m, 2H), 6.76 (dd, *J* = 9.0, 2.8 Hz,

1H), 3.25 (t, *J* = 4.7 Hz, 4H), 2.84 (t, *J* = 4.9 Hz, 4H), 1.76 (s, 2H). ^{13}C NMR (101 MHz, DMSO-d_6) δ 168.87, 164.38, 161.97, 151.18, 143.92, 141.23, 140.83, 131.96, 131.36, 130.91, 126.42, 123.25, 123.23, 120.86, 119.95, 117.24, 116.69, 115.76, 113.89, 113.67, 111.31, 61.21, 52.86, 47.98. HRMS (AP-ESI) m/z clad for [$C_{25}H_{22}Cl_2FN_5O$] [M + H]$^+$ 498.1258; found, 498.1258.

2-(4-(3,4-dichlorophenyl)piperazin-1-yl)-N-(5-(4-methoxyphenyl)-1H-indazol-3-yl)acetamide (6b). mp: 200-201℃; Yield: 64%; ^{1}H NMR (400 MHz, DMSO-d_6) *δ:* 12.98 (s, 1H), 10.84 (s, 1H), 8.12 (s, 1H), 7.69 (d, *J* = 8.8 Hz, 1H), 7.56 (d, *J* = 8.7 Hz, 1H), 7.51 – 7.31 (m, 2H), 7.31 – 7.13 (m, 3H), 6.97 (ddd, *J* = 26.0, 8.6, 2.7 Hz, 2H), 3.83 (s, 5H), 3.35 (s, 6H). ^{13}C NMR (101 MHz, DMSO-d_6) δ 160.19, 150.22, 142.81, 141.03, 139.98, 132.53, 132.09, 131.08, 130.45, 126.68, 120.31, 119.61, 117.20, 117.02, 116.12, 113.00, 112.60, 111.22, 55.61, 52.00. HRMS (AP-ESI) m/z clad for HRMS (AP-ESI) m/z clad for [$C_{26}H_{25}Cl_2N_5O_2$] [M + H]$^+$ 510.1459; found, 510.1459.

N-(5-(4-methoxyphenyl)-1H-indazol-3-yl)-2-(4-(3-methoxyphenyl)piperazin-1-yl)acetamide (6c). mp: 188-189℃; Yield: 67%; ^{1}H NMR (400 MHz, Chloroform-*d*) *δ:* 10.16 (s, 1H), 9.65 (s, 1H), 8.22 (s, 1H), 7.64 (d, *J* = 8.5 Hz, 1H), 7.43 (d, *J* = 8.7 Hz, 1H), 7.38 (d, *J* = 7.9 Hz, 1H), 7.24 (d, *J* = 7.7 Hz, 1H), 7.18 (t, *J* = 2.1 Hz, 1H), 6.97 – 6.84 (m, 5H), 3.88 (s, 3H), 3.80 (s, 3H), 3.34 (s, 2H), 3.19 (t, *J* = 4.8 Hz, 4H), 2.87 (t, *J* = 4.9 Hz, 4H). ^{13}C NMR (101 MHz, DMSO-d_6) *δ:* 168.89, 160.20, 153.37, 145.88, 142.88, 141.12, 140.74, 132.39, 130.46, 126.62, 120.55, 119.61, 117.88, 114.71, 112.93, 112.61, 111.09, 61.40, 55.64, 55.57, 53.35, 50.08.

HRMS (AP-ESI) m/z clad for $[C_{27}H_{29}N_5O_3]$ $[M + H]^+$ 472.2343; found, 472.2343.

N-(5-(4-methoxyphenyl)-1H-indazol-3-yl)-2-(4-(4-(trifluoromethyl)phenyl)piperazin-1-yl)acetamide (6d). mp: 187-189℃; Yield: 66%; ^{1}H NMR (400 MHz, Chloroform-*d*) δ: 10.06 (s, 1H), 9.58 (s, 1H), 8.23 (s, 1H), 7.66 (dd, J = 8.7, 1.7 Hz, 1H), 7.49 (dd, J = 25.3, 8.6 Hz, 3H), 7.37 (t, J = 7.9 Hz, 1H), 7.28 – 7.16 (m, 2H), 7.01 – 6.85 (m, 3H), 3.88 (s, 3H), 3.38 (dd, J = 10.7, 5.5 Hz, 6H), 2.86 (t, J = 5.0 Hz, 4H). ^{13}C NMR (101 MHz, Chloroform-*d*) δ: 168.27, 159.96, 152.98, 142.77, 140.96, 134.25, 129.80, 127.80, 126.48, 126.44, 119.96, 114.83, 113.35, 112.14, 110.28, 61.61, 55.32, 53.28, 48.24. HRMS (AP-ESI) m/z clad for $[C_{27}H_{26}F_3N_5O_2]$ $[M + H]^+$ 510.2111; found, 510.2112.

2-(4-(4-chlorobenzyl)piperazin-1-yl)-N-(5-(4-methoxyphenyl)-1H-indazol-3-yl)acetamide (6e). mp: 140-141℃; Yield: 54%; ^{1}H NMR (400 MHz, Chloroform-*d*) δ: 10.23 (s, 1H), 9.71 (s, 1H), 8.25 (s, 1H), 7.64 (d, J = 8.6 Hz, 1H), 7.53 – 7.33 (m, 3H), 7.31 – 7.15 (m, 3H), 7.15 – 6.96 (m, 2H), 6.91 (dd, J = 8.1, 2.6 Hz, 1H), 3.88 (s, 3H), 3.38 (s, 2H), 3.17 (t, J = 4.6 Hz, 4H), 2.91 (t, J = 4.6 Hz, 4H), 2.07 (s, 1H), 1.42 – 1.17 (m, 1H). ^{13}C NMR (101 MHz, DMSO-*d*$_6$) δ: 168.88, 160.20, 149.50, 142.88, 141.12, 140.76, 132.38, 130.81, 130.45, 128.55, 128.13, 126.62, 124.36, 121.37, 120.58, 119.61, 112.93, 112.61, 111.08, 61.33, 55.57, 53.37, 51.31. HRMS (AP-ESI) m/z clad for $[C_{27}H_{28}FN_5O_2]$ $[M + H]^+$ 490.2004; found, 490.2004.

2-(4-(3,4-dichlorophenyl)piperazin-1-yl)-N-(5-(3,5-difluorophenyl)-1H-indazol-3-yl)acetamide (6f). mp: 201-203℃; Yield: 59%; ^{1}H NMR (400 MHz, DMSO-*d*$_6$) δ: 12.86 (s, 1H), 10.20 (s, 1H), 8.14 (d, J = 1.8 Hz, 1H), 7.72 (dd, J = 8.8,

1.8 Hz, 1H), 7.55 (d, *J* = 8.7 Hz, 1H), 7.39 (dd, *J* = 8.7, 2.9 Hz, 3H), 7.34 – 7.04 (m, 2H), 6.95 (dd, *J* = 9.0, 2.9 Hz, 1H), 3.28 (d, *J* = 5.8 Hz, 6H), 2.73 (t, *J* = 5.0 Hz, 4H). ^{13}C NMR (101 MHz, DMSO-d_6) *δ* 168.94, 164.49, 162.18, 151.19, 145.08, 141.41, 140.99, 131.96, 130.92, 129.95, 126.34, 121.21, 119.96, 117.21, 116.69, 115.77, 111.35, 110.27, 110.09, 110.02, 102.73, 102.48, 61.22, 52.87, 47.98. HRMS (AP-ESI) m/z clad for [$C_{25}H_{21}Cl_2F_2N_5O$] [M + H]$^+$ 516.1164; found, 516.1164.

N-(5-(3,5-difluorophenyl)-1H-indazol-3-yl)-2-(4-(4-(trifluoromethyl)phenyl)piperazin-1-yl)acetamide (6g). mp: 170-171℃; Yield: 55%; ^{1}H NMR (400 MHz, Chloroform-*d*) *δ:* 10.06 (s, 1H), 9.58 (s, 1H), 8.23 (s, 1H), 7.66 (dd, *J* = 8.7, 1.7 Hz, 1H), 7.52 (d, *J* = 8.5 Hz, 2H), 7.46 (d, *J* = 8.7 Hz, 1H), 7.37 (t, *J* = 7.9 Hz, 1H), 7.24 (d, *J* = 7.7 Hz, 1H), 6.99 – 6.88 (m, 3H), 3.38 (dd, *J* = 10.7, 5.5 Hz, 6H), 2.86 (t, *J* = 5.0 Hz, 4H). ^{13}C NMR (101 MHz, DMSO-d_6) *δ* 168.88, 164.48, 162.05, 153.71, 145.17, 141.65, 140.91, 129.74, 126.61, 126.57, 126.10, 124.13, 121.15, 118.40, 118.09, 117.20, 114.66, 110.22, 109.96, 102.39, 61.26, 52.90, 47.45. HRMS (AP-ESI) m/z clad for [$C_{26}H_{22}F_5N_5O$] [M + H]$^+$ 516.1817; found, 516.1817.

N-(5-(3,5-difluorophenyl)-1H-indazol-3-yl)-2-(4-(2,4-difluorophenyl)piperazin-1-yl)acetamide (6h). mp: 165-167℃; Yield: 47%; ^{1}H NMR (400 MHz, Chloroform-*d*) *δ:* 9.94 (s, 1H), 9.70 (s, 1H), 8.40 – 8.26 (m, 1H), 7.61 (dd, J = 8.8, 1.8 Hz, 1H), 7.48 (d, J = 8.8 Hz, 1H), 7.17 (dt, J = 7.0, 2.1 Hz, 2H), 7.06 – 6.89 (m, 1H), 6.90 – 6.73 (m, 3H), 3.38 (s, 2H), 3.17 (d, J = 5.0 Hz, 4H), 2.92 (d, J = 4.8 Hz, 4H). ^{13}C NMR (101 MHz, DMSO-d_6) *δ* 168.88, 142.02, 141.29, 140.95,

137.18, 132.09, 131.49, 129.91, 128.78, 127.38, 126.31, 121.09, 120.55, 120.49, 117.18, 111.55, 111.39, 105.33, 105.08, 61.28, 53.29, 50.87. HRMS (AP-ESI) m/z clad for [$C_{25}H_{21}F_4N_5O$] [M + H]$^+$ 484.1755; found, 484.1755.

2-(4-(4-Bromophenyl)piperazin-1-yl)-N-(5-(3,5-difluorophenyl)-1H-indazol-3-yl)acetamide (6i). mp: 184-186℃; Yield: 59%; ^{1}H NMR (400 MHz, Chloroform-*d*) *δ:* 9.97 (s, 1H), 9.66 (s, 1H), 8.38 – 8.27 (m, 1H), 7.60 (dd, *J* = 8.8, 1.7 Hz, 1H), 7.47 (d, *J* = 8.7 Hz, 1H), 7.43 – 7.32 (m, 2H), 7.17 (dt, *J* = 7.0, 2.1 Hz, 2H), 6.91 – 6.69 (m, 3H), 3.37 (s, 2H), 3.28 (t, *J* = 5.0 Hz, 4H), 2.88 (t, *J* = 5.0 Hz, 4H). ^{13}C NMR (101 MHz, DMSO-d_6) *δ* 168.91, 164.63, 164.48, 162.18, 162.05, 150.62, 141.49, 140.92, 131.93, 129.84, 126.22, 121.19, 117.79, 117.19, 111.51, 110.39, 110.25, 109.99, 102.44, 61.29, 53.03, 48.34. HRMS (AP-ESI) m/z clad for [$C_{25}H_{22}BrF_2N_5O$] [M + H]$^+$ 526.1049; found, 526.1049.

2-(4-(4-Chlorophenyl)piperazin-1-yl)-N-(5-(3,5-difluorophenyl)-1H-indazol-3-ylacetamide (6j). mp: 179-181℃; Yield: 50%; ^{1}H NMR (400 MHz, Chloroform-*d*) *δ:* 10.01 (s, 1H), 9.66 (s, 1H), 8.41 – 8.20 (m, 1H), 7.60 (dd, *J* = 8.8, 1.7 Hz, 1H), 7.47 (dd, *J* = 8.8, 0.8 Hz, 1H), 7.25 (d, *J* = 9.0 Hz, 2H), 7.17 (dt, *J* = 7.0, 2.2 Hz, 2H), 6.93 – 6.84 (m, 2H), 6.84 - 6.69 (m, 1H), 3.37 (s, 2H), 3.35 – 3.19 (m, 4H), 2.88 (t, *J* = 5.0 Hz, 4H). ^{13}C NMR (101 MHz, DMSO-d_6) *δ* 168.91, 164.62, 162.18, 150.29, 145.13, 145.03, 141.48, 140.92, 129.84, 129.07, 126.22, 122.75, 121.19, 117.33, 117.19, 110.24, 110.18, 109.99, 102.44, 61.29, 53.06, 48.48. HRMS (AP-ESI) m/z clad for [$C_{25}H_{22}ClF_5N_5O$] [M + H]$^+$ 482.1554; found, 482.1554.

N-(5-(3,5-difluorophenyl)-1H-indazol-3-yl)-2-(4-phenylpiperazin-1-yl)acetamide (6k). mp: 180-182℃; Yield: 61%; ^{1}H NMR (400 MHz, Chloroform-d) *δ:* 10.16 (s, 1H), 9.70 (s, 1H), 8.29 (d, J = 1.7 Hz, 1H), 7.58 (dd, J = 8.8, 1.7 Hz, 1H), 7.45 (d, J = 8.7 Hz, 1H), 7.34 – 7.29 (m, 2H), 7.16 (dt, J = 7.1, 2.2 Hz, 2H), 7.03 – 6.87 (m, 3H), 6.86 – 6.72 (m, 1H), 3.47 – 3.26 (m, 6H), 2.96 – 2.83 (m, 4H). ^{13}C NMR (101 MHz, Chloroform-d) *δ:* 168.54, 164.59, 164.46, 162.13, 150.96, 141.49, 132.01, 129.23, 127.17, 121.50, 120.23, 116.35, 110.37, 110.29, 110.10, 110.03, 102.11, 61.67, 53.69, 49.41.HRMS (AP-ESI) m/z clad for [$C_{25}H_{23}F_{2}N_{5}O$] [M + H]$^{+}$ 448.1943; found, 448.1944.

N-(5-(3,5-difluorophenyl)-1H-indazol-3-yl)-2-(4-(pyrimidin-2-yl)piperazin-1-yl)acetamide (6l). mp: 200-201℃; Yield: 46%; ^{1}H NMR (400 MHz, DMSO-*d*$_6$) *δ:* 8.36 (d, *J* = 4.7 Hz, 2H), 8.13 (d, *J* = 1.6 Hz, 1H), 7.72 – 7.63 (m, 1H), 7.55 (d, *J* = 8.9 Hz, 1H), 7.43 – 7.33 (m, 2H), 7.18 (tt, *J* = 9.2, 2.3 Hz, 1H), 6.63 (t, *J* = 4.7 Hz, 1H), 3.83 (t, *J* = 5.0 Hz, 6H), 2.65 (t, *J* = 5.1 Hz, 4H). ^{13}C NMR (101 MHz, DMSO-*d*$_6$) *δ* 168.98, 164.62, 164.48, 162.18, 162.04, 161.67, 158.39, 145.11, 141.45, 140.94, 129.88, 126.26, 121.21, 117.22, 111.44, 110.56, 110.26, 110.19, 110.01, 102.44, 61.39, 53.06, 43.74. HRMS (AP-ESI) m/z clad for [$C_{23}H_{21}F_{2}N_{7}O$] [M + H]$^{+}$ 450.1848; found, 450.1848.

N-(5-(4-chloro-3-fluorophenyl)-1H-indazol-3-yl)-2-(4-(3,4-dichlorophenyl)piperazin-1-yl)acetamide (6m). mp: 185-187℃; Yield: 64%; ^{1}H NMR (400 MHz, Chloroform-*d*) *δ:* 9.99 (s, 1H), 9.62 (s, 1H), 8.34 – 8.27 (m, 1H), 7.59 (dd, *J* = 8.8, 1.8 Hz, 1H), 7.51 – 7.30 (m, 5H), 7.00 (d, *J* = 2.9 Hz, 1H), 6.78 (dd,

J = 8.9, 2.9 Hz, 1H), 3.45 – 3.19 (m, 6H), 2.86 (t, *J* = 4.9 Hz, 4H). ^{13}C NMR (101 MHz, DMSO-d_6) δ 168.84, 159.27, 156.82, 151.18, 142.66, 141.40, 140.83, 131.96, 131.44, 130.91, 129.89, 126.15, 124.22, 120.94, 119.95, 118.24, 118.07, 117.23, 116.68, 115.76, 115.35, 115.14, 61.22, 52.85, 47.98. 61.63, 53.34, 48.94. HRMS (AP-ESI) m/z clad for $[C_{25}H_{21}Cl_3FN_5O]$ $[M + H]^+$ 532.0868; found, 532.0868.

N-(5-(4-chloro-3-fluorophenyl)-1H-indazol-3-yl)-2-(4-(3-methoxyphenyl)piperazin-1-yl)acetamide (6n). mp: 174-176℃; Yield: 57%; ^{1}H NMR (400 MHz, Chloroform-*d*) *δ:* 9.72 (s, 1H), 8.29 (s, 1H), 7.63 – 7.52 (m, 1H), 7.44 (t, *J* = 7.6 Hz, 2H), 7.38 (d, *J* = 2.0 Hz, 1H), 6.94 (d, *J* = 8.8 Hz, 2H), 6.88 (d, *J* = 8.8 Hz, 2H), 3.80 (s, 3H), 3.37 (s, 2H), 3.20 (t, *J* = 4.8 Hz, 4H), 2.89 (t, *J* = 4.8 Hz, 4H). ^{13}C NMR (101 MHz, DMSO-d_6) δ 168.93, 159.27, 156.83, 153.37, 145.87, 142.62, 141.31, 140.96, 131.46, 130.01, 126.30, 124.25, 124.21, 121.07, 118.30, 117.88, 117.19, 115.38, 114.71, 111.37, 61.38, 55.63, 53.37, 50.07. HRMS (AP-ESI) m/z clad for $[C_{26}H_{25}ClFN_5O_2]$ $[M + H]^+$ 494.1754; found, 494.1754.

N-(5-(4-chloro-3-fluorophenyl)-1H-indazol-3-yl)-2-(4-(4-(trifluoromethyl)phenyl)piperazin-1-yl)acetamide (6o). mp: 183-185℃; Yield: 60%; ^{1}H NMR (400 MHz, Chloroform-*d*) *δ:* 10.08 (s, 1H), 9.64 (s, 1H), 8.30 (s, 1H), 7.76 – 7.33 (m, 7H), 6.96 (d, *J* = 8.4 Hz, 2H), 3.71 – 3.18 (m, 6H), 2.88 (t, *J* = 4.9 Hz, 4H). ^{13}C NMR (101 MHz, Chloroform-*d*) *δ:* 168.41, 159.52, 152.99, 141.40, 141.08, 132.05, 130.76, 127.21, 126.51, 126.48, 123.65, 123.61, 121.31, 116.56, 115.43, 115.21, 114.86, 110.40, 61.69, 53.37, 48.25. HRMS (AP-ESI) m/z clad for $[C_{26}H_{22}ClF_4N_5O]$ $[M + H]^+$ 532.1522; found, 532.1522.

***N*-(5-(4-chloro-3-fluorophenyl)-1*H*-indazol-3-yl)-2-(4-(2-chlorophenyl)piperazin-1-yl)acetamide (6p).** mp 163-165℃; Yield: 51%; ^{1}H NMR (400 MHz, Chloroform-*d*) *δ:* 9.97 (s, 1H), 9.76 (s, 1H), 8.33 (d, *J* = 1.9 Hz, 1H), 7.59 (dd, *J* = 8.8, 1.8 Hz, 1H), 7.50 – 7.36 (m, 5H), 7.25 (dd, *J* = 7.6, 1.6 Hz, 1H), 7.13 – 6.99 (m, 2H), 3.39 (s, 2H), 3.20 (t, *J* = 4.7 Hz, 4H), 2.93 (t, *J* = 4.7 Hz, 4H). ^{13}C NMR (101 MHz, Chloroform-*d*) *δ:* 168.74, 148.87, 141.42, 141.29, 130.75, 128.91, 127.67, 127.19, 124.05, 123.66, 123.63, 121.44, 120.45, 116.59, 115.43, 115.22, 110.34, 61.72, 53.87, 51.34. HRMS (AP-ESI) m/z clad for [$C_{25}H_{22}Cl_2FN_5O$] [M + H]$^+$ 498.1258; found, 498.1258.

***N*-(5-(3,4-dichlorophenyl)-1*H*-indazol-3-yl)-2-(4-(2,4-difluorophenyl)piperazin-1-yl)acetamide (6q).** mp: 160-162℃; Yield: 42%; ^{1}H NMR (400 MHz, Chloroform-*d*) *δ:* 10.30 (s, 1H), 9.72 (s, 1H), 8.25 (s, 1H), 7.70 (d, *J* = 2.0 Hz, 1H), 7.58 – 7.51 (m, 1H), 7.50 – 7.41 (m, 3H), 6.92 (dt, *J* = 9.8, 4.3 Hz, 1H), 6.84 (ddt, *J* = 12.3, 7.2, 3.5 Hz, 2H), 3.38 (s, 2H), 3.26 – 3.09 (m, 4H), 2.90 (t, *J* = 4.8 Hz, 4H). ^{13}C NMR (101 MHz, DMSO-d_6) δ 168.88, 160.20, 149.50, 142.89, 141.12, 140.76, 132.39, 130.80, 130.45, 128.54, 128.13, 126.62, 124.35, 121.36, 120.59, 119.62, 117.24, 112.94, 112.60, 111.09, 61.34, 55.56, 53.38, 51.30. HRMS (AP-ESI) m/z clad for [$C_{25}H_{21}Cl_2F_2N_5O$] [M + H]$^+$ 516.1164; found, 516.1164.

2-(4-(4-Bromophenyl)piperazin-1-yl)-N-(5-(3,4-dichlorophenyl)-1H-indazol-3-yl)acetamide (6r). mp: 189-191℃; Yield: 45%; ^{1}H NMR (400 MHz, Chloroform-*d*) *δ:* 9.99 (s, 1H), 9.66 (s, 1H), 8.30 – 8.27 (m, 1H), 7.72 (d, *J* = 2.0 Hz, 1H), 7.59 (dd, *J* = 8.8, 1.7 Hz, 1H), 7.53 – 7.45 (m, 3H), 7.41 – 7.35 (m, 2H), 6.90 –

6.74 (m, 2H), 3.43 – 3.19 (m, 6H), 2.88 (t, *J* = 5.0 Hz, 4H). ^{13}C NMR (101 MHz, DMSO-d_6) *δ:* 168.83, 150.61, 142.10, 140.81, 132.08, 131.93, 131.47, 129.81, 129.53, 128.73, 127.33, 126.02, 120.95, 117.79, 117.19, 110.39, 61.31, 53.01, 48.34. HRMS (AP-ESI) m/z clad for [$C_{25}H_{22}BrCl_2N_5O$] [M + H]$^+$ 558.0458; found, 558.0458.

2-(4-(4-Chlorophenyl)piperazin-1-yl)-N-(5-(3,4-dichlorophenyl)-1H-indazol-3-yl)acetamide (6s). mp: 179-182℃; Yield: 55%; ^{1}H NMR (400 MHz, Chloroform-*d*) *δ:* 10.04 (s, 1H), 9.66 (s, 1H), 8.28 (d, *J* = 1.7 Hz, 1H), 7.72 (d, *J* = 2.0 Hz, 1H), 7.58 (dd, *J* = 8.7, 1.7 Hz, 1H), 7.52 – 7.44 (m, 3H), 7.27 – 7.22 (m, 2H), 6.94 – 6.82 (m, 2H), 3.44– 3.20 (m, 6H), 2.88 (t, *J* = 4.9 Hz, 4H). ^{13}C NMR (101 MHz, DMSO-d_6) *δ* 168.86, 150.28, 142.05, 141.33, 140.83, 132.09, 131.49, 129.86, 129.68, 129.07, 128.76, 127.36, 126.20, 122.75, 120.98, 117.33, 117.22, 61.29, 53.04, 48.48, 39.66.

HRMS (AP-ESI) m/z clad for [$C_{25}H_{22}Cl_3N_5O$] [M + H]$^+$ 514.0963; found, 514.0963.

2-(4-(4-Cyanophenyl)piperazin-1-yl)-N-(5-(3,4-dichlorophenyl)-1H-indazol-3-yl)acetamide (6t). mp: 194-197℃; Yield: 58%; ^{1}H NMR (400 MHz, Chloroform-*d*) *δ:* 9.76 (s, 1H), 9.60 (s, 1H), 8.31 (s, 1H), 7.74 (d, *J* = 2.0 Hz, 1H), 7.64 – 7.59 (m, 1H), 7.57 – 7.47 (m, 5H), 6.92 (d, *J* = 8.9 Hz, 2H), 3.46 (t, *J* = 4.9 Hz, 4H), 3.38 (s, 2H), 2.88 (t, *J* = 5.0 Hz, 4H). ^{13}C NMR (101 MHz, DMSO-d_6) *δ* 168.84, 153.63, 142.06, 141.41, 140.84, 133.80, 132.09, 131.48, 129.65, 128.76, 127.35, 126.15, 120.95, 120.51, 117.23, 114.54, 98.67, 61.17, 52.78, 46.84. HRMS (AP-ESI) m/z clad for [$C_{26}H_{22}Cl_2N_6O$] [M + H]$^+$ 505.1305; found, 505.1305.

***N*-(5-(3,4-dichlorophenyl)-1*H*-indazol-3-yl)-2-(4-(pyrimidin-2-yl)piperazin-1-yl)acetamide (6u)** mp: 195-197℃; Yield: 51%; ^{1}H NMR (400 MHz, Chloroform-*d*) *δ:* 10.06 (s, 1H), 9.72 (s, 1H), 8.41 – 8.25 (m, 3H), 7.72 (d, *J* = 1.9 Hz, 1H), 7.59 (dd, *J* = 8.7, 1.7 Hz, 1H), 7.55 – 7.44 (m, 3H), 6.55 (t, *J* = 4.8 Hz, 1H), 3.97 (t, *J* = 5.1 Hz, 4H), 3.35 (s, 2H), 2.78 (t, *J* = 5.1 Hz, 4H). ^{13}C NMR (101 MHz, Chloroform-*d*) *δ:* 168.54, 161.54, 157.79, 141.39, 132.76, 130.65, 129.10, 127.25, 126.69, 120.99, 110.44, 110.29, 61.87, 53.54, 43.78. HRMS (AP-ESI) m/z clad for [$C_{23}H_{21}Cl_2N_7O$] $[M + H]^+$ 482.1257; found, 482.1257.

7. ^{1}H NMR, ^{13}C NMR, and HRMS spectra for target compounds.

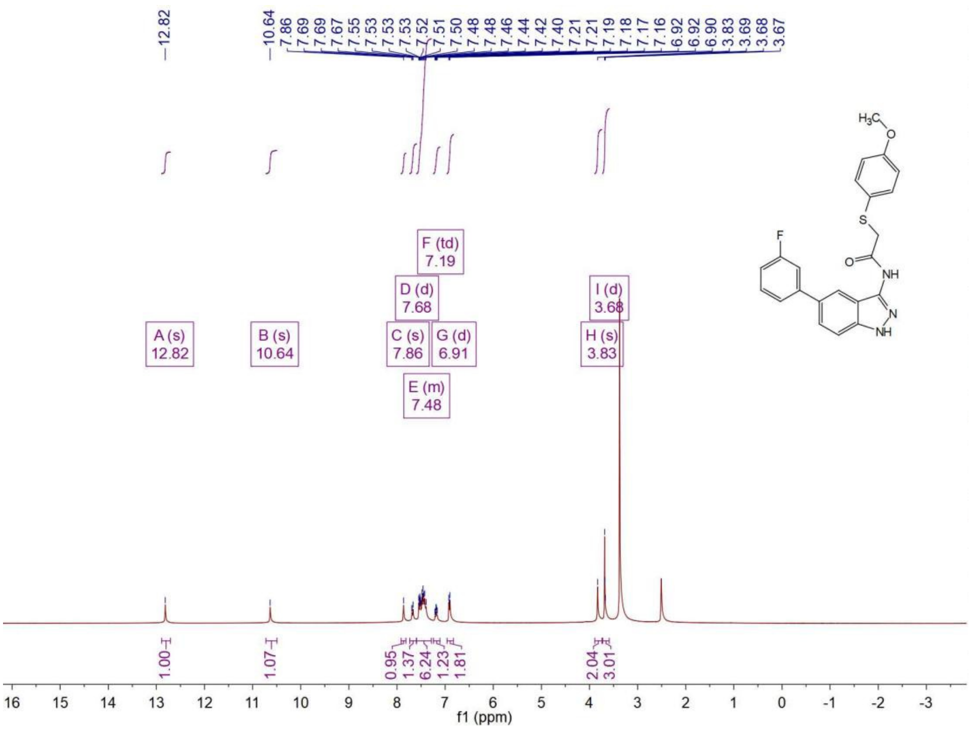

Figure S1. The ^{1}H NMR spectrum of **5a**.

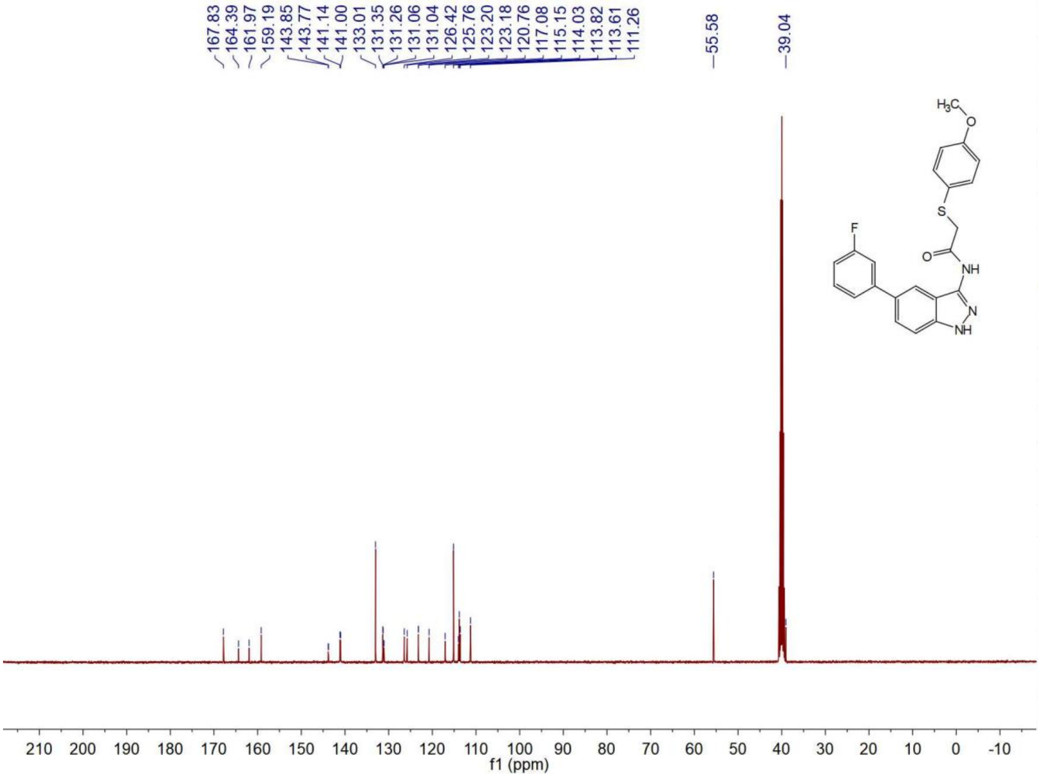

Figure S2. The ^{13}C NMR spectrum of **5a**.

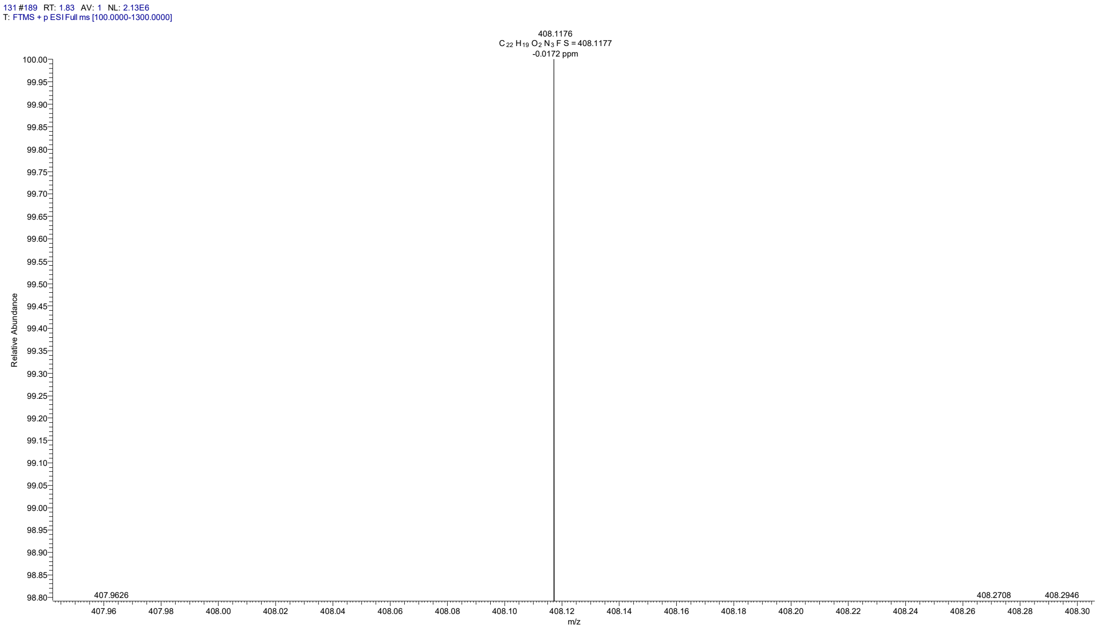

Figure S3. The HRMS spectrum of **5a**.

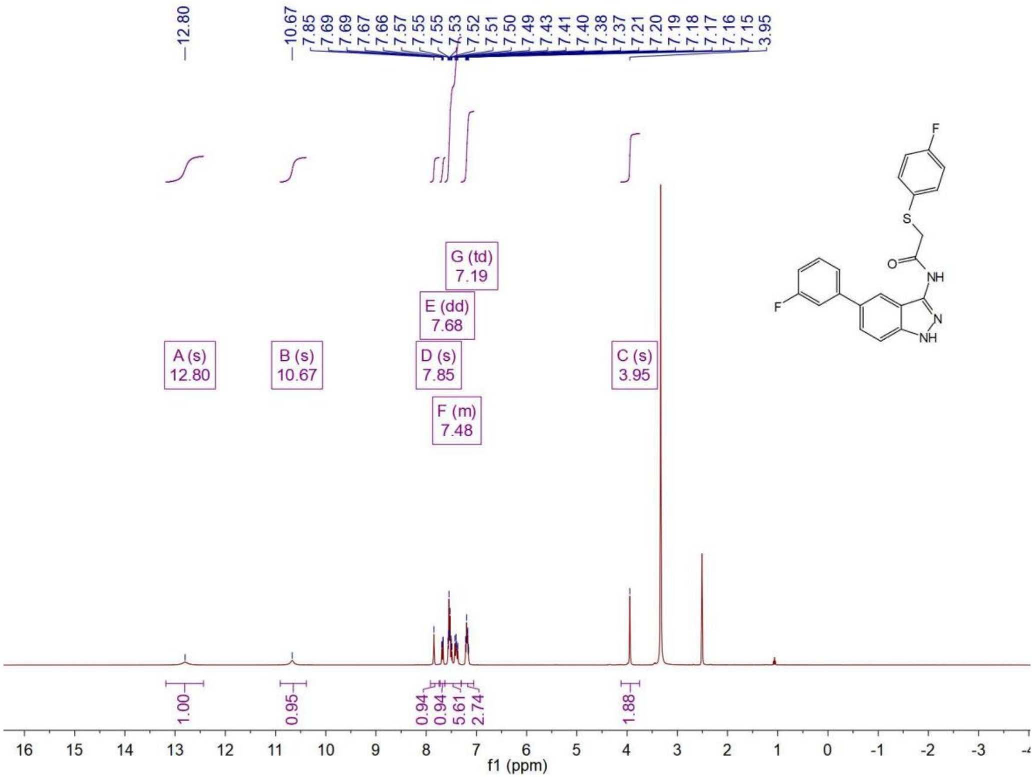

Figure S4. The ^{1}H NMR spectrum of **5b**.

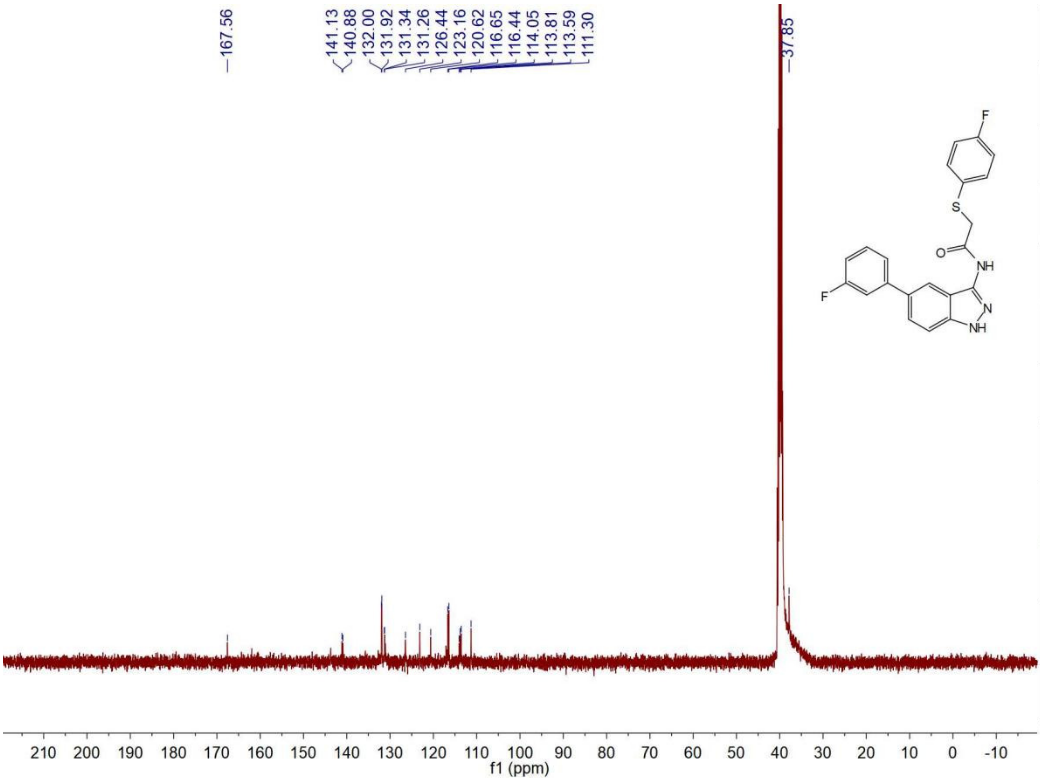

Figure S5. The ^{13}C NMR spectrum of **5b**.

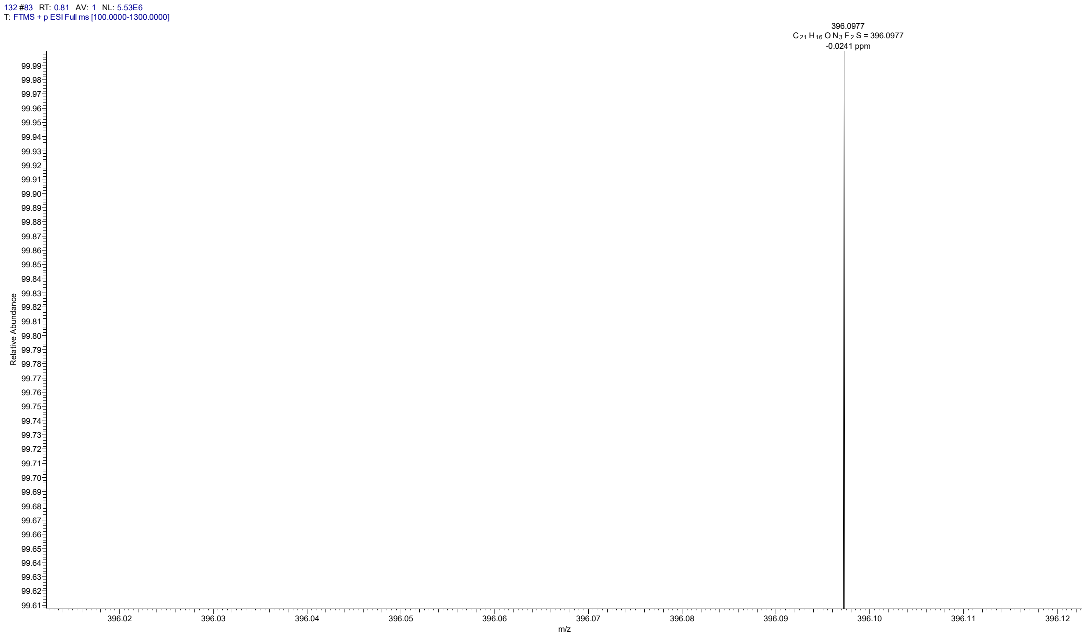

Figure S6. The HRMS spectrum of **5b**.

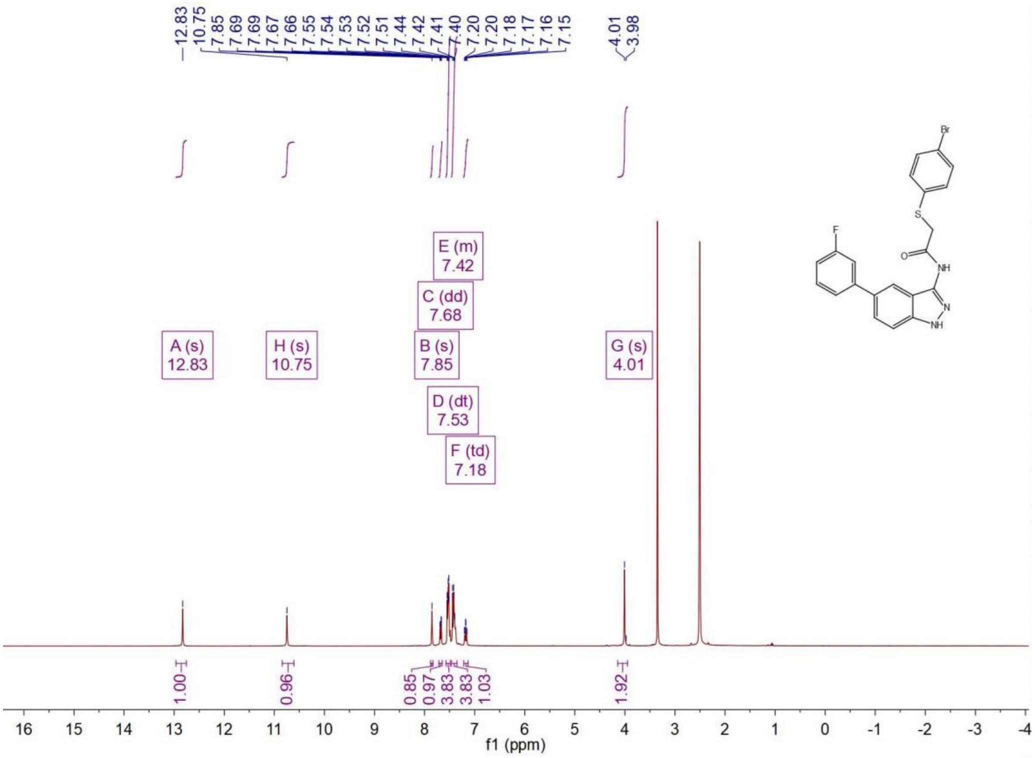

Figure S7. The ^{1}H NMR spectrum of **5c**.

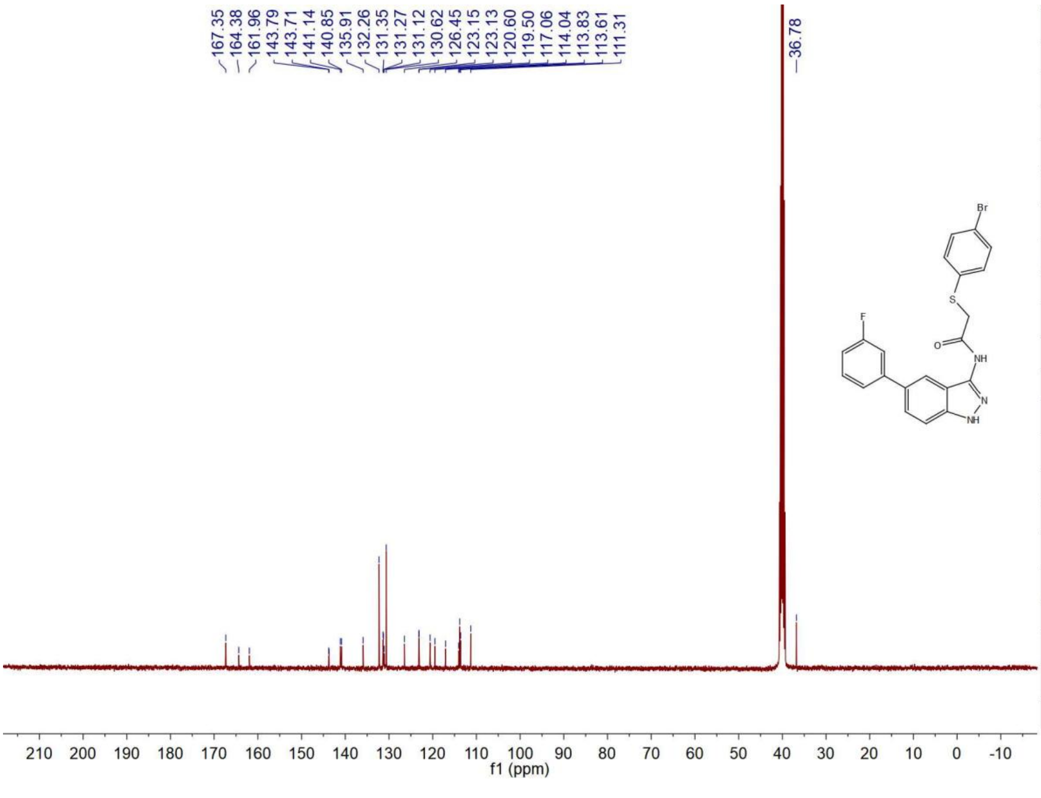

Figure S8. The ^{13}C NMR spectrum of **5c**.

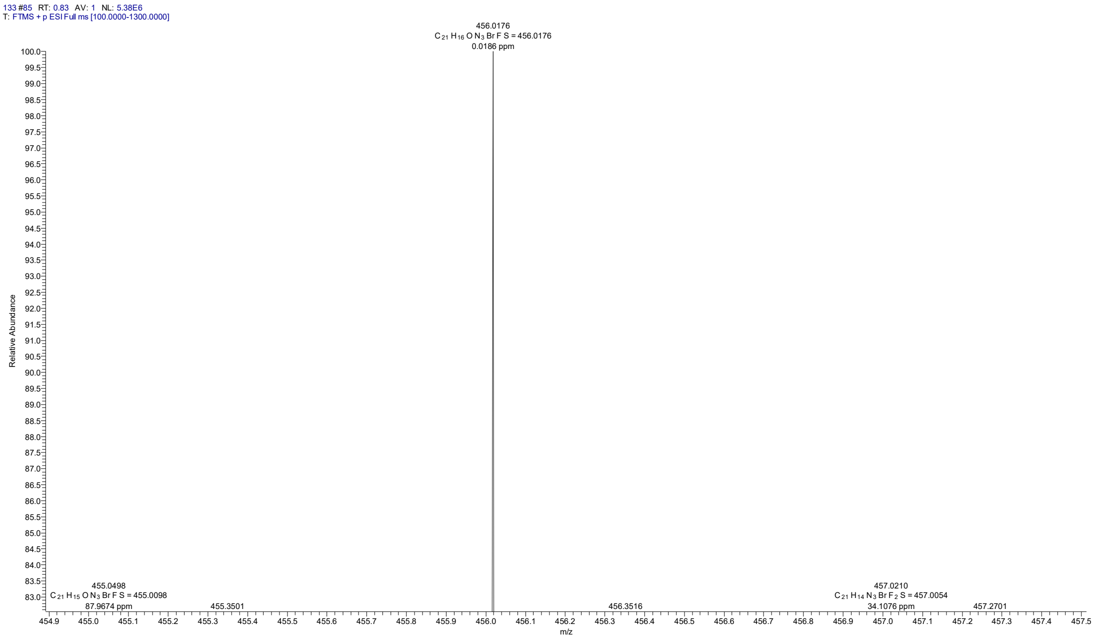

Figure S9. The HRMS spectrum of **5c**.

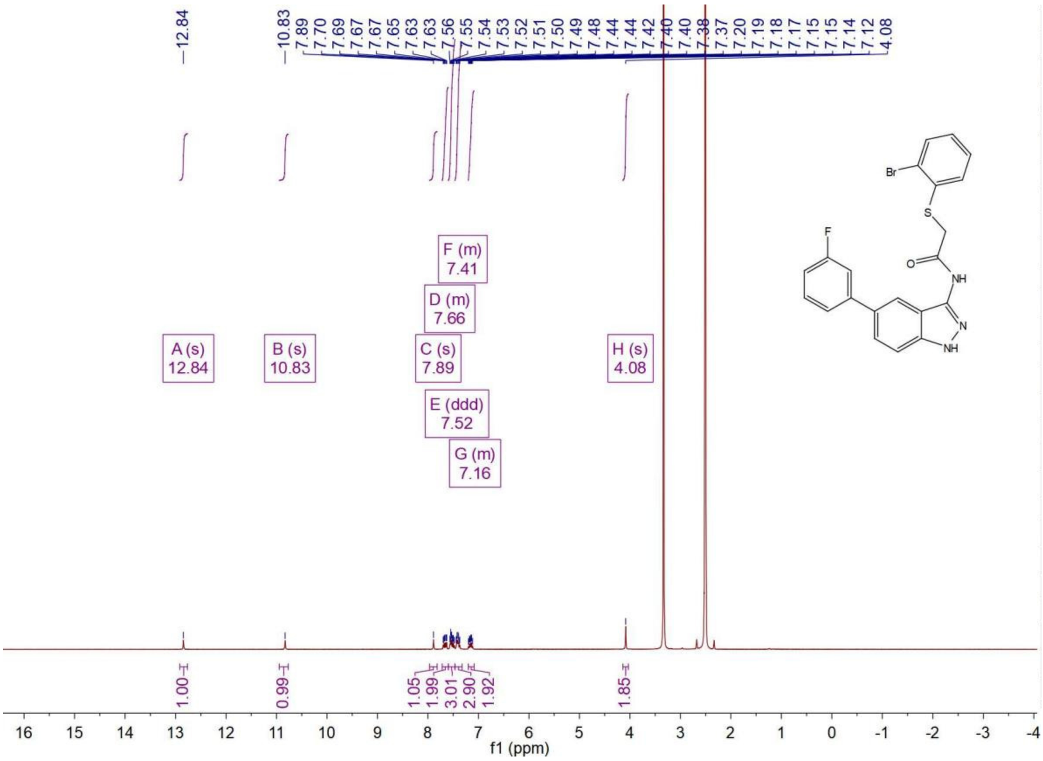

Figure S10. The ^{1}H NMR spectrum of **5d**.

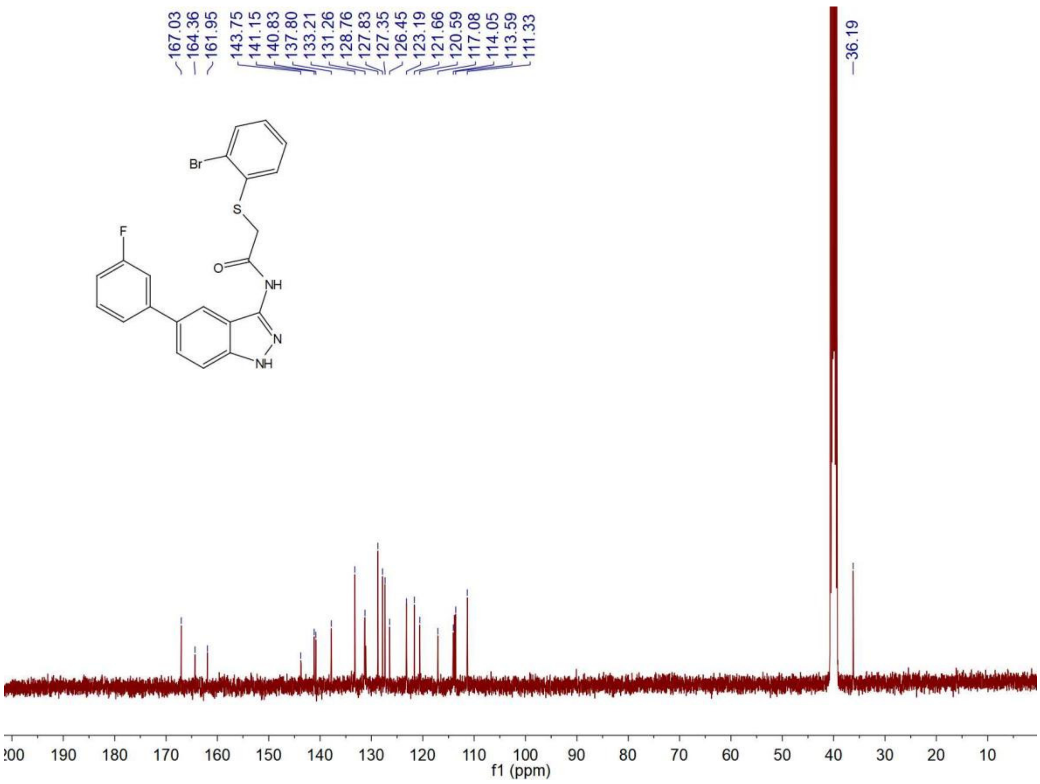

Figure S11. The ^{13}C NMR spectrum of **5d**.

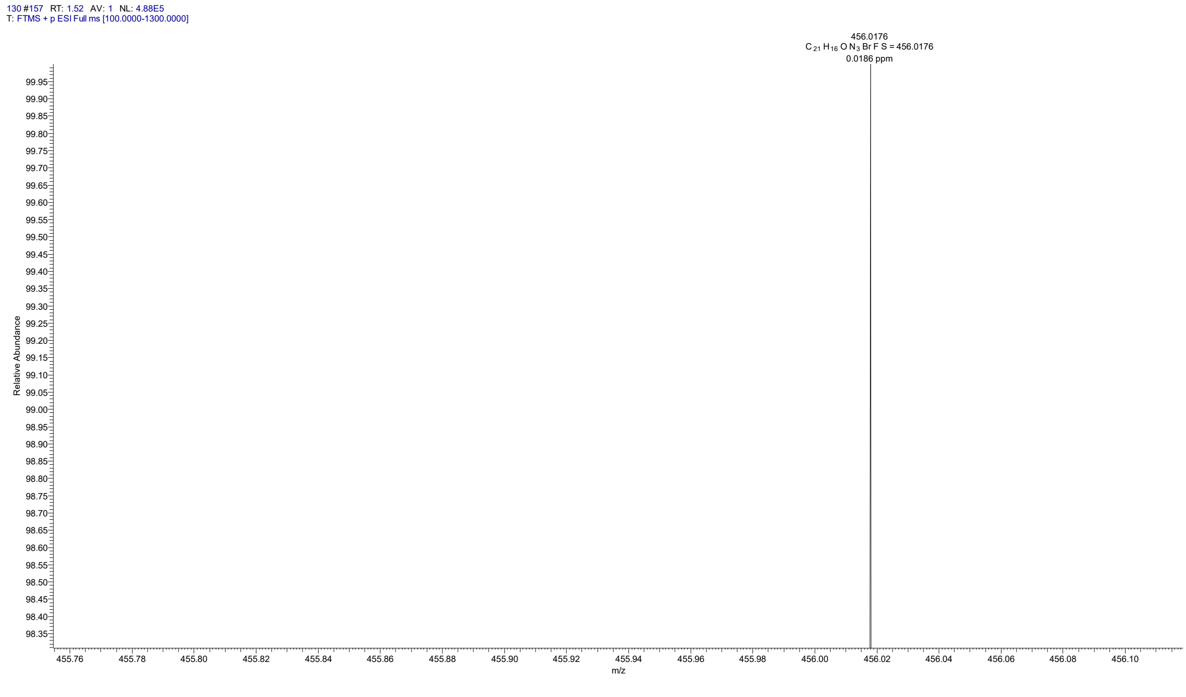

Figure S12. The HRMS spectrum of **5d**.

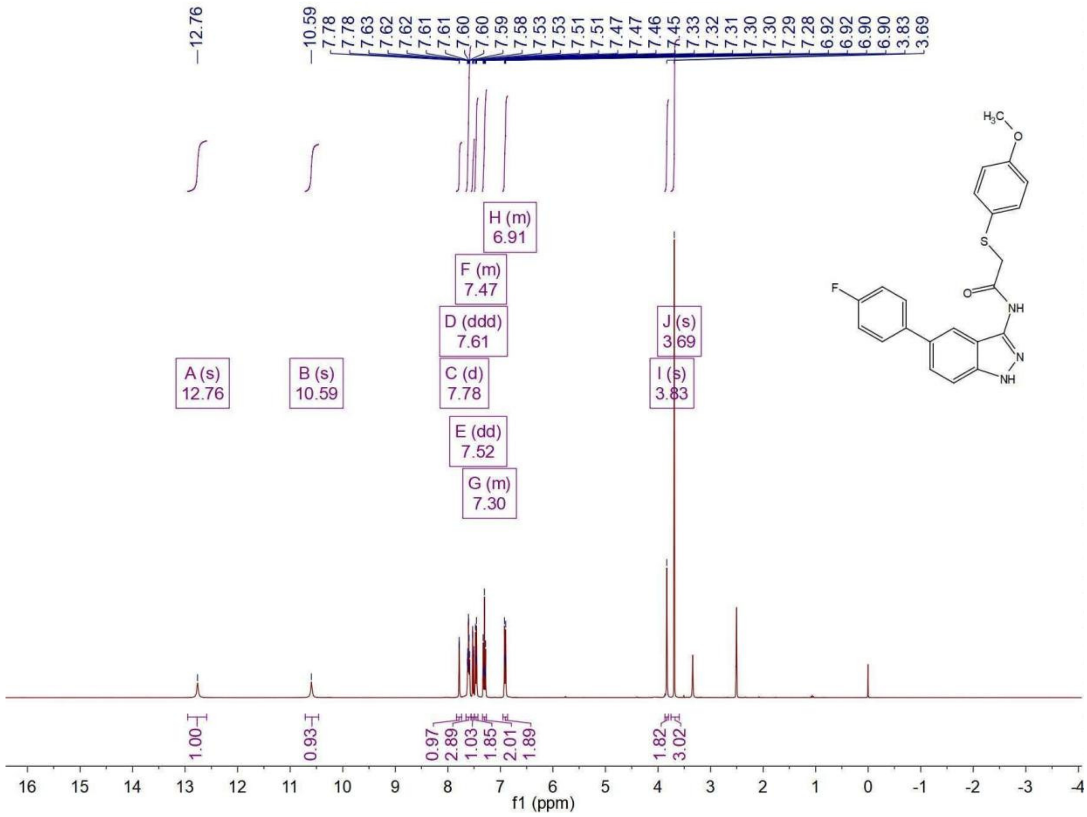

Figure S13. The ^{1}H NMR spectrum of **5e**.

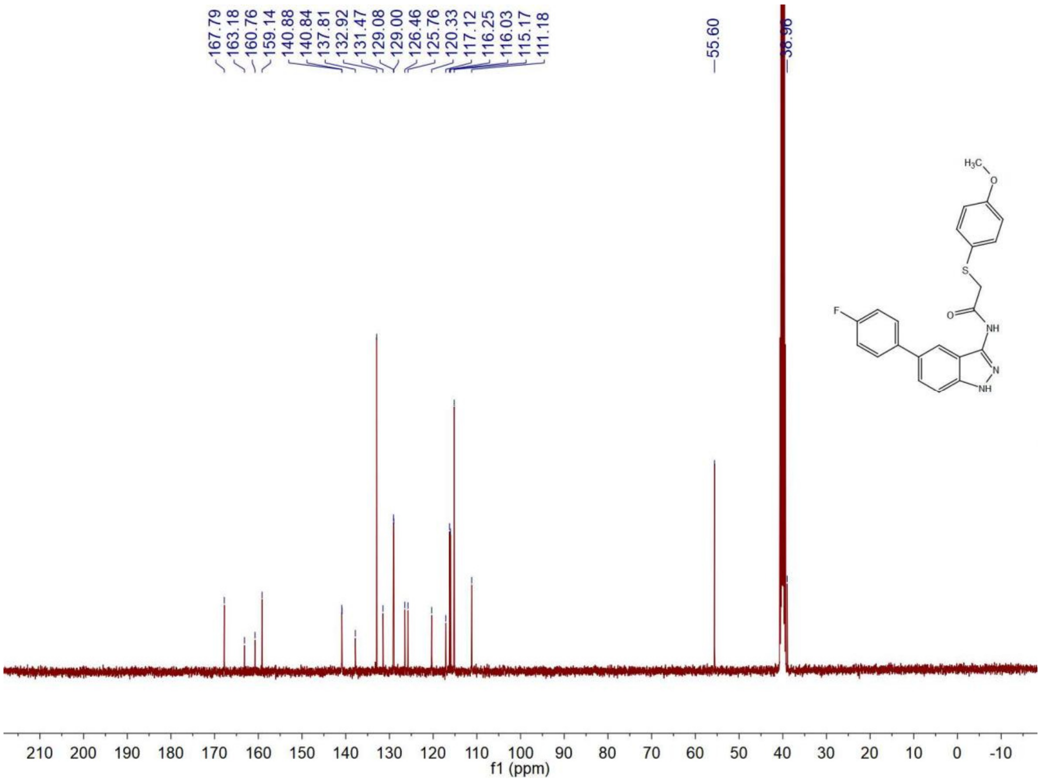

Figure S14. The ^{13}C NMR spectrum of 5e.

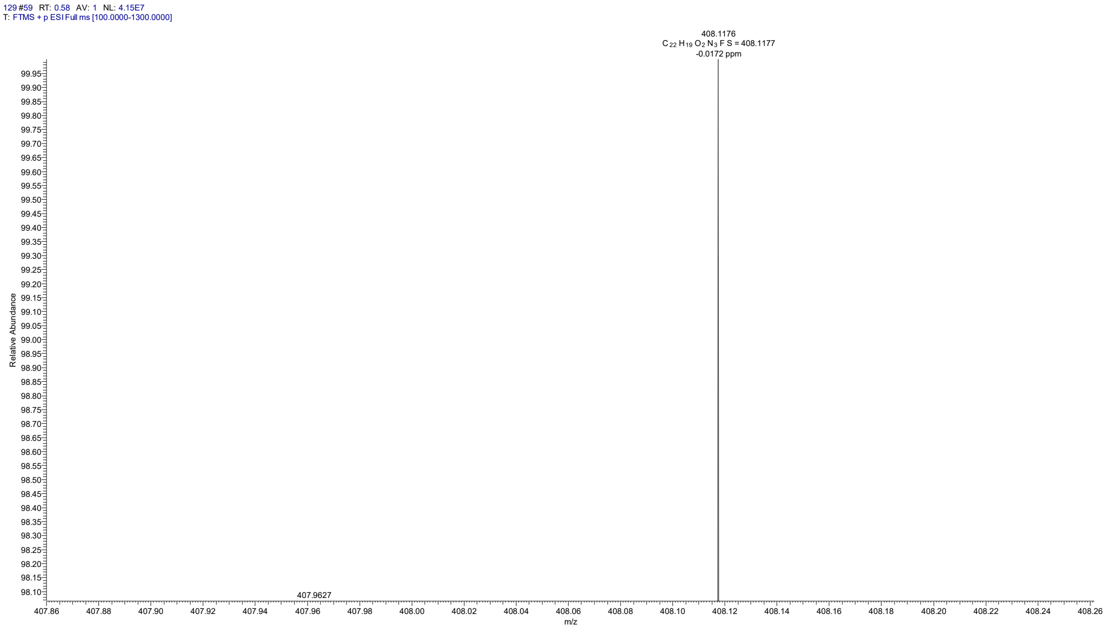

Figure S15. The HRMS spectrum of **5e**.

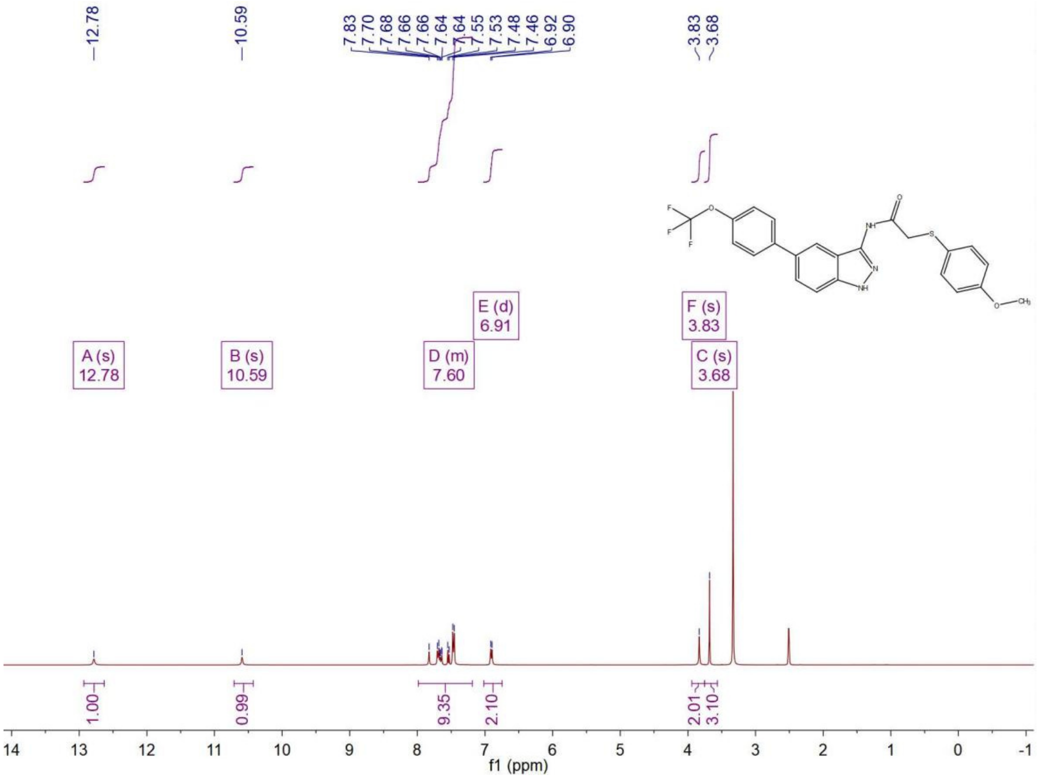

Figure S16. The ¹H NMR spectrum of **5f**.

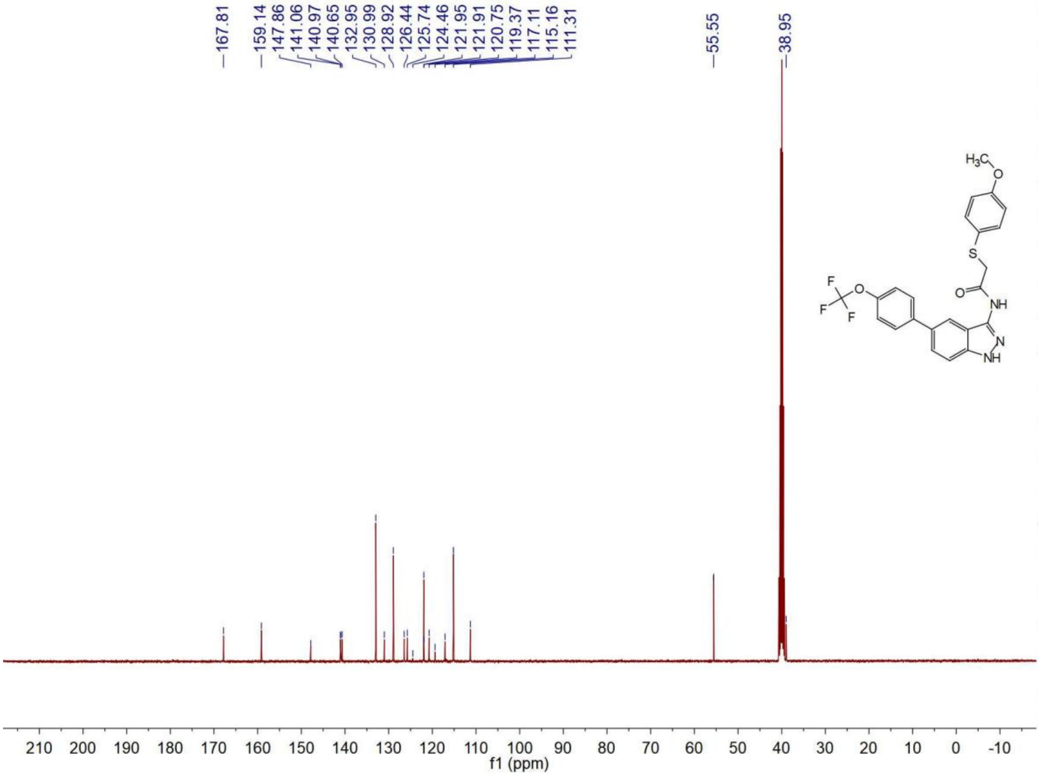

Figure S17. The ^{13}C NMR spectrum of **5f**.

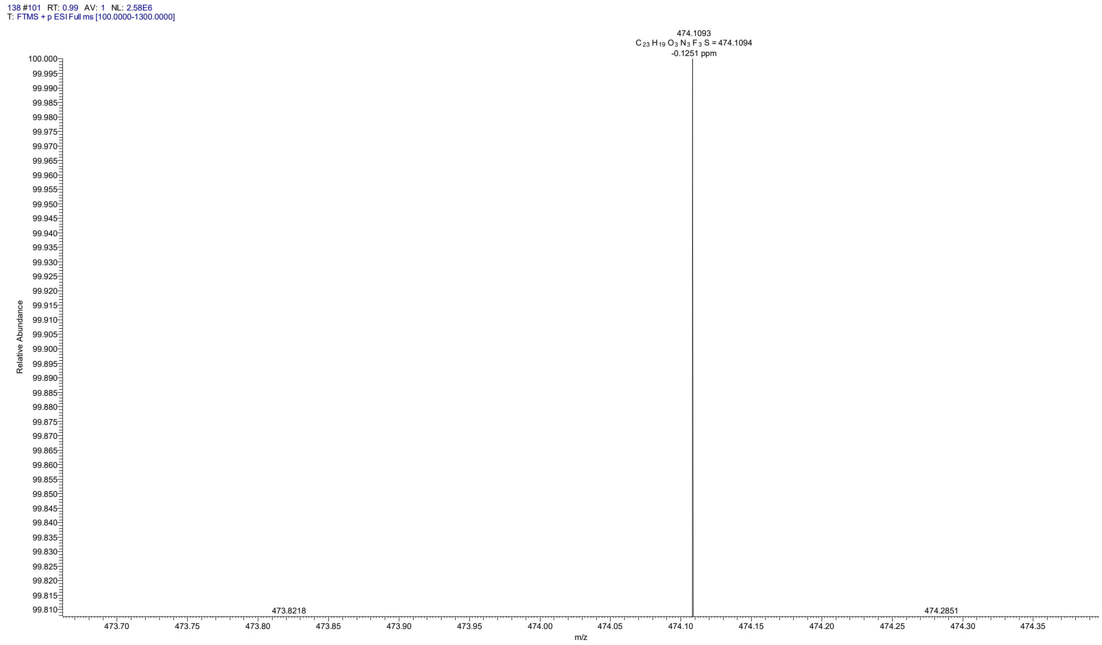

Figure S18. The HRMS spectrum of **5f**.

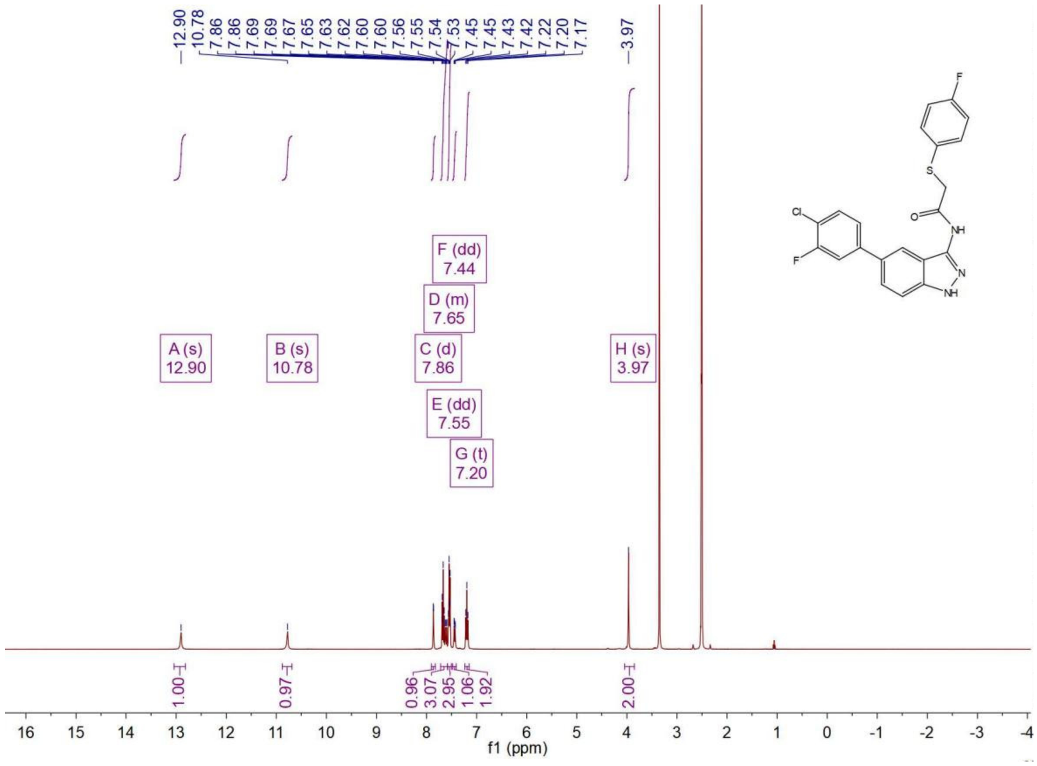

Figure S19. The ^{1}H NMR spectrum of **5g**.

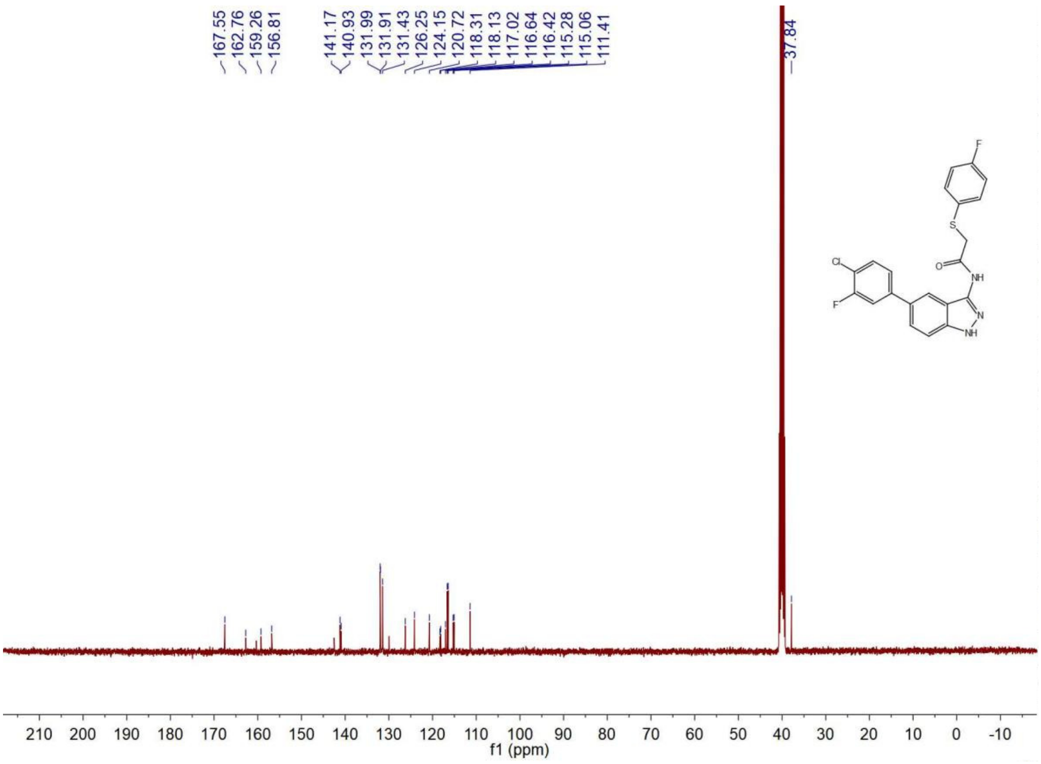

Figure S20. The ^{13}C NMR spectrum of **5g**.

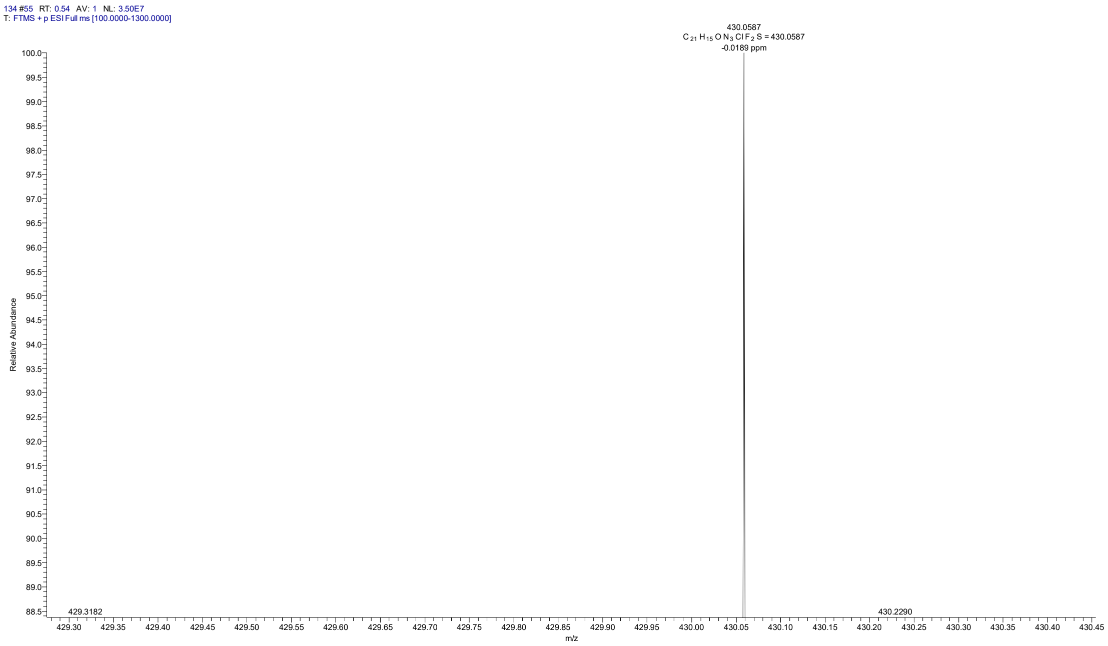

Figure S21. The HRMS spectrum of **5g**.

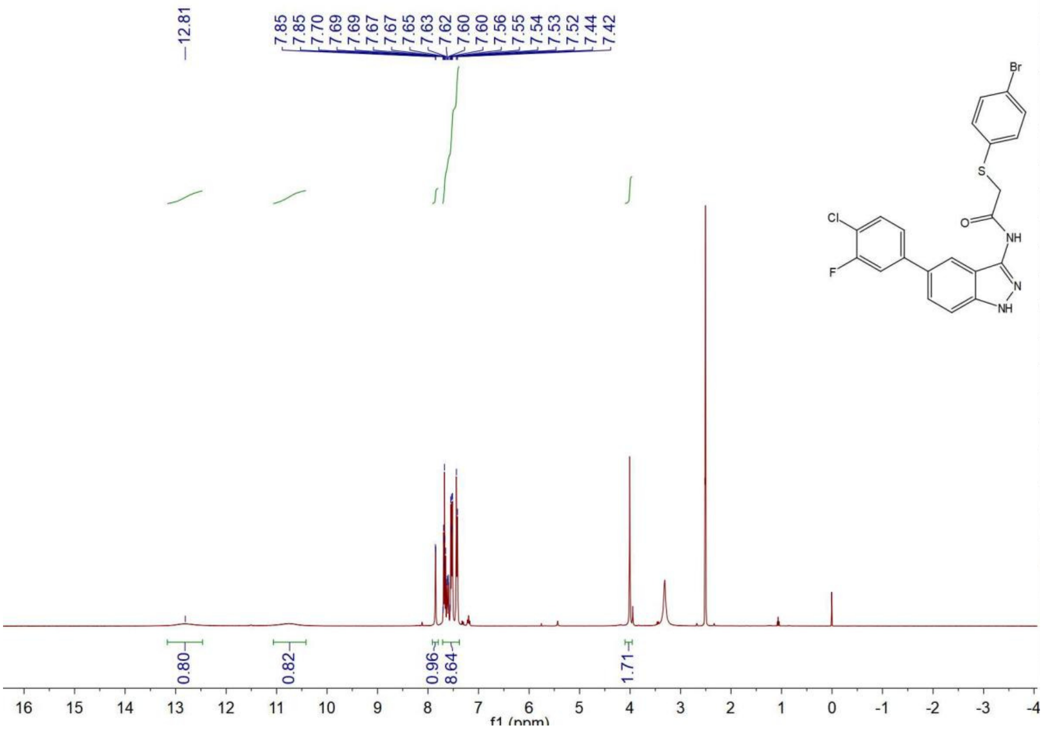

Figure S22. The ^{1}H NMR spectrum of **5h**.

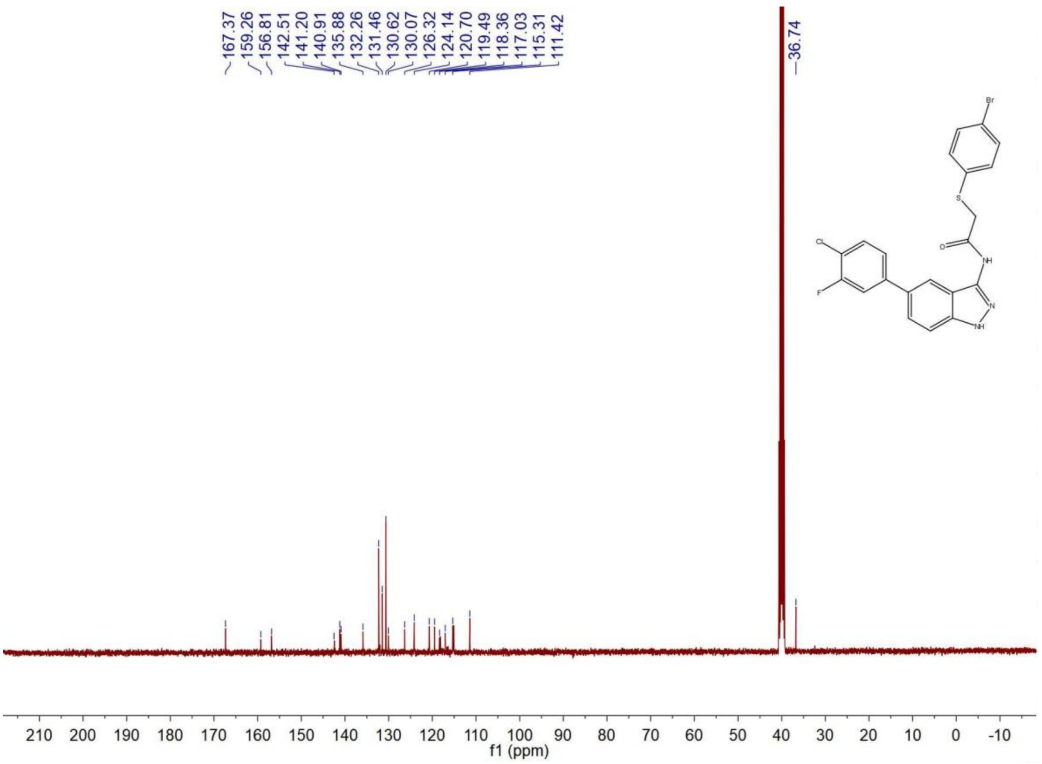

Figure S23. The ^{13}C NMR spectrum of **5h**.

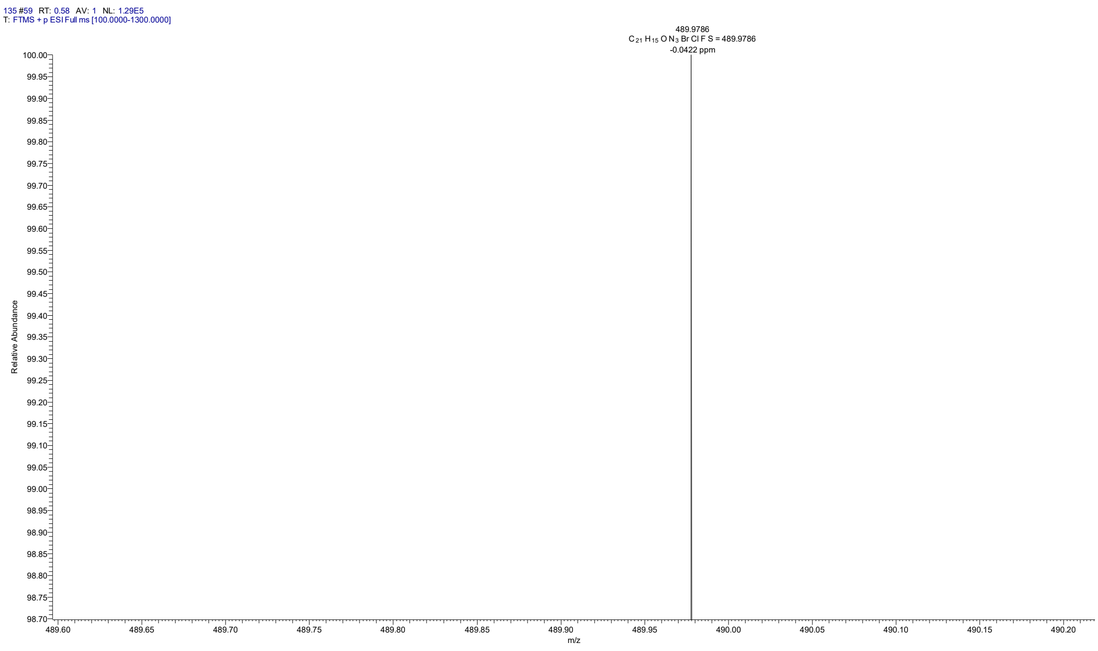

Figure S24. The HRMS spectrum of **5h**.

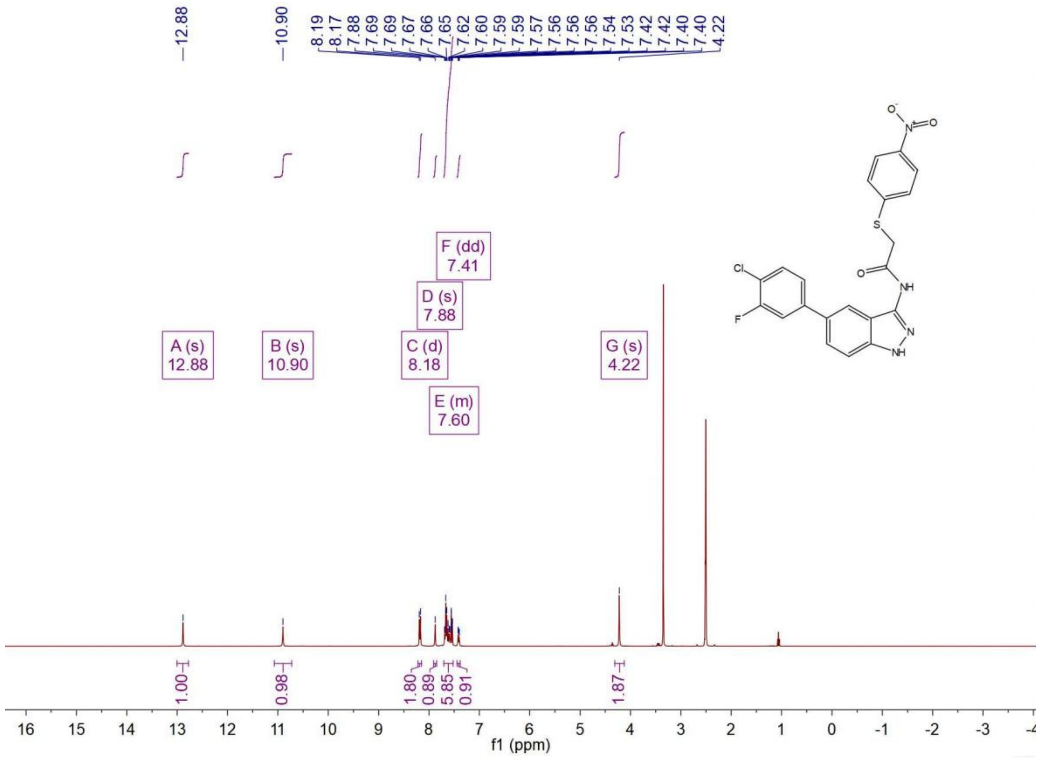

Figure S25. The ^{1}H NMR spectrum of **5i**.

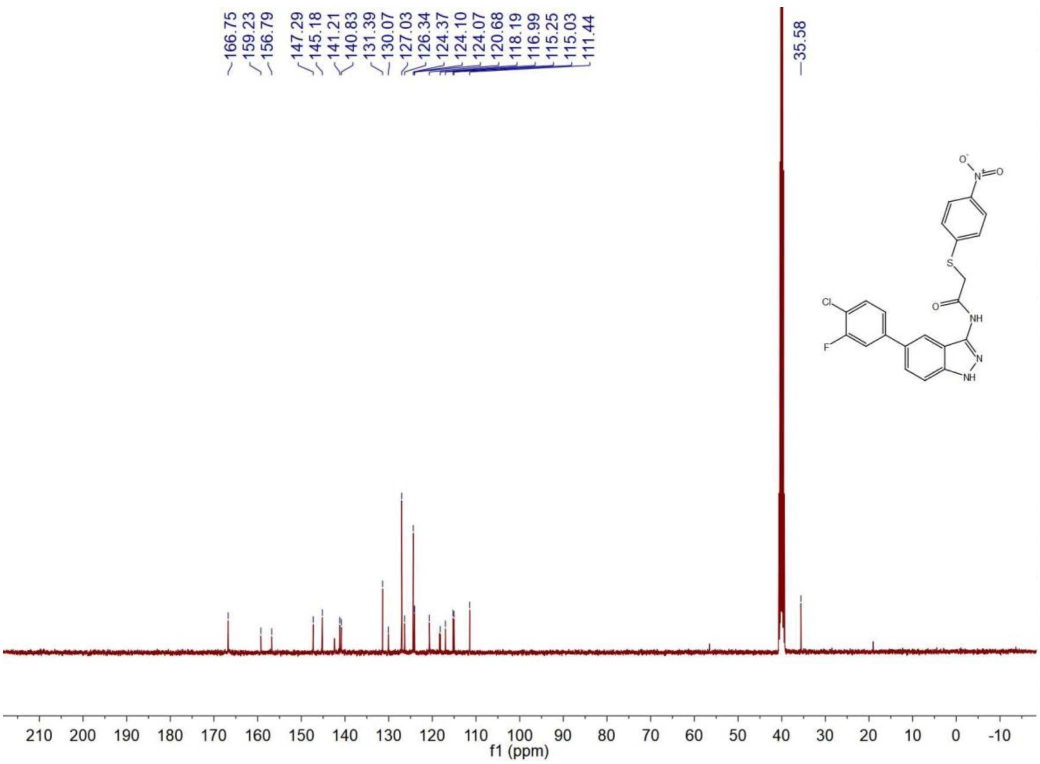

Figure S26. The ^{13}C NMR spectrum of **5i**.

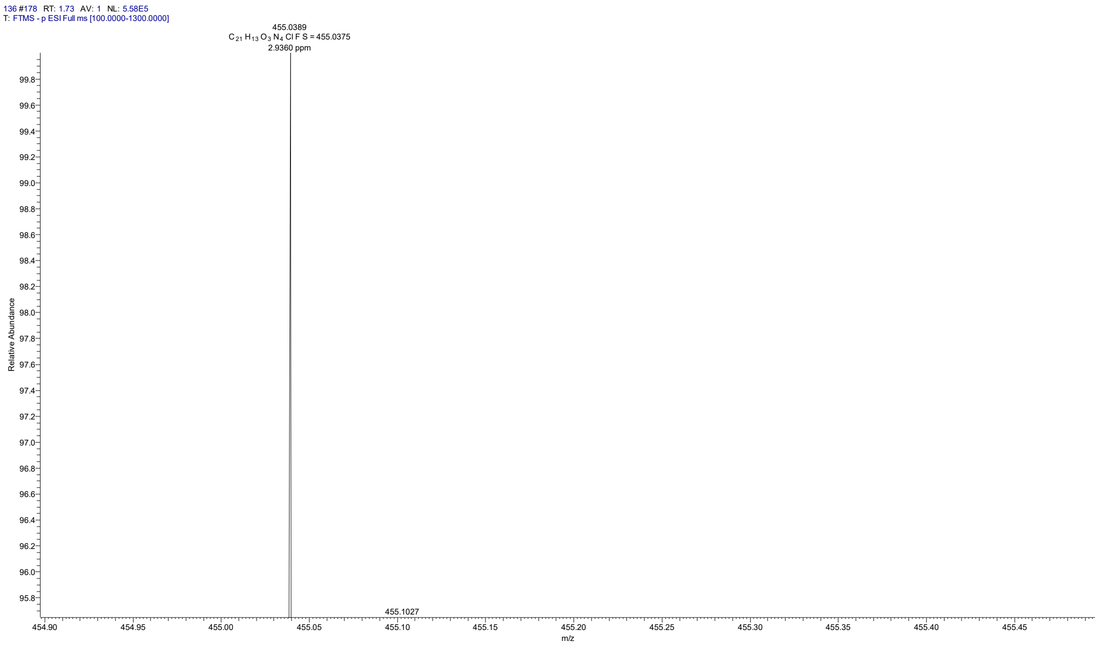

Figure S27. The HRMS spectrum of **5i**.

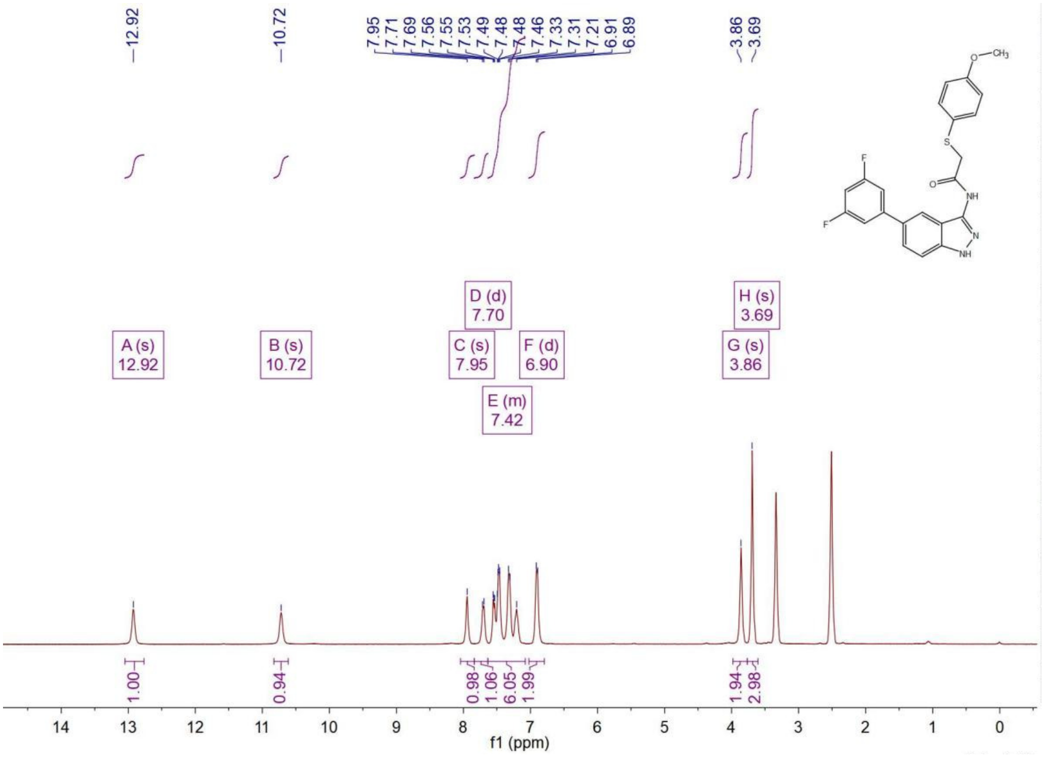

Figure S28. The ^{1}H NMR spectrum of **5j**.

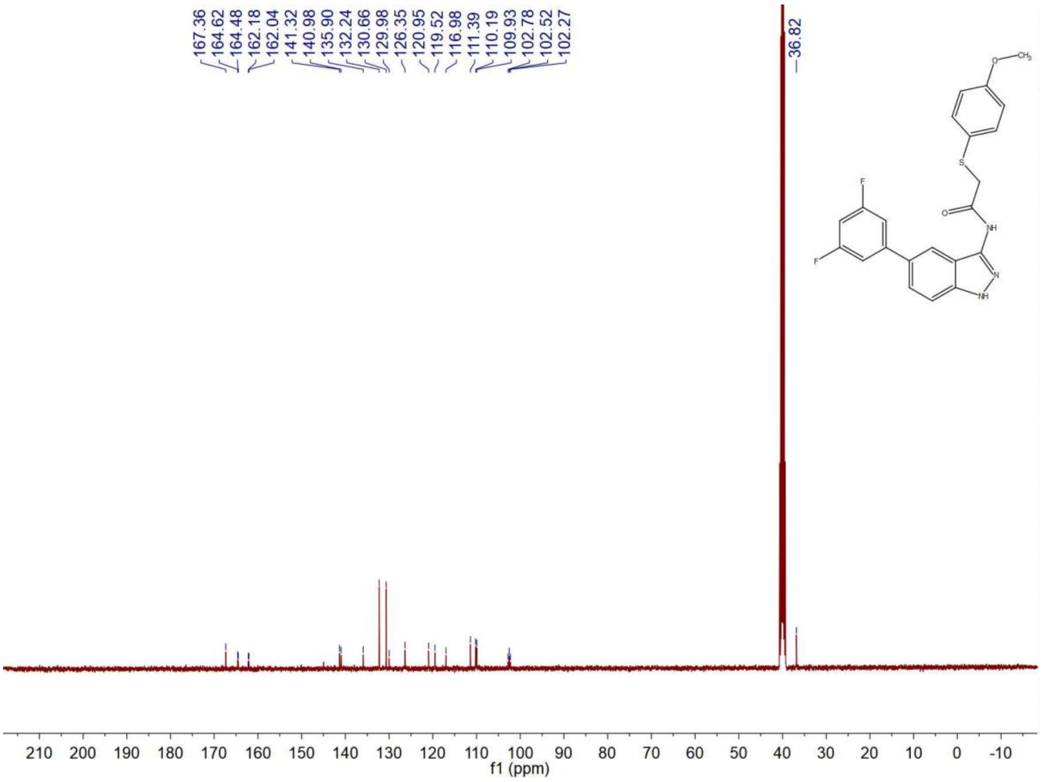

Figure S29. The ^{13}C NMR spectrum of **5j**.

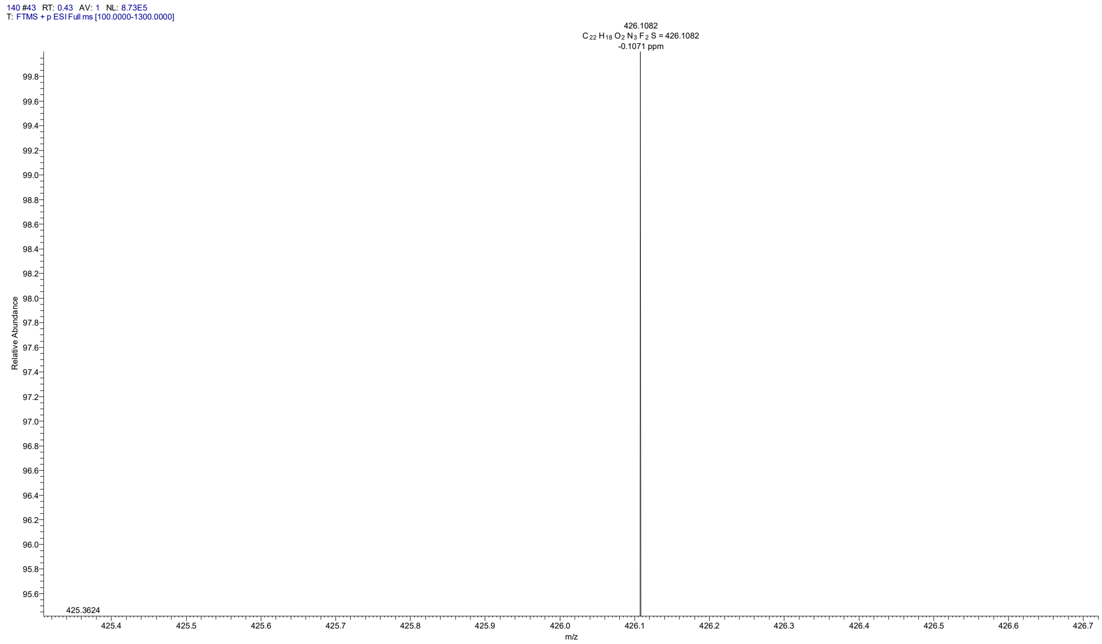

Figure S30. The HRMS spectrum of **5j**.

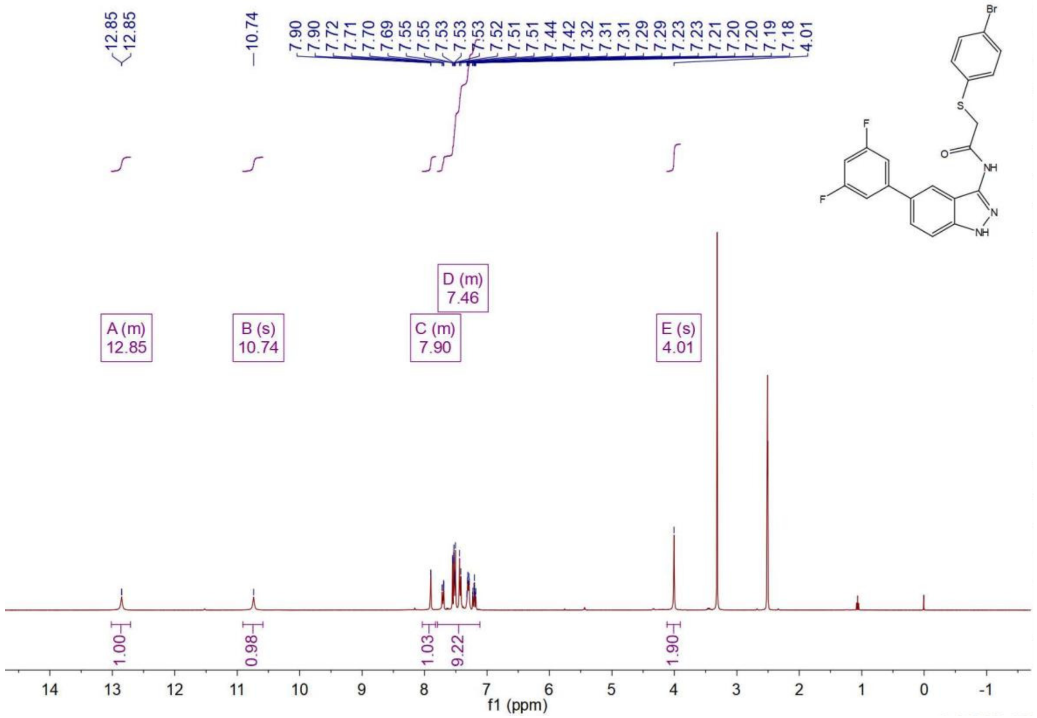

Figure S31. The ^{1}H NMR spectrum of **5k**.

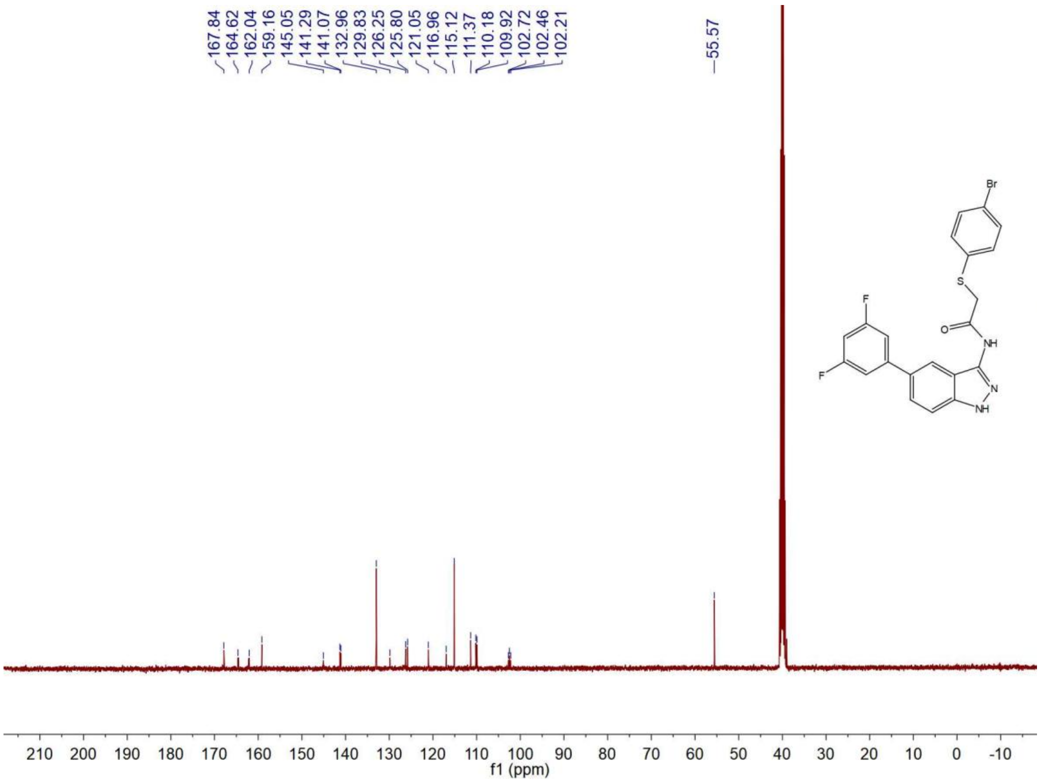

Figure S32. The ^{13}C NMR spectrum of **5k**.

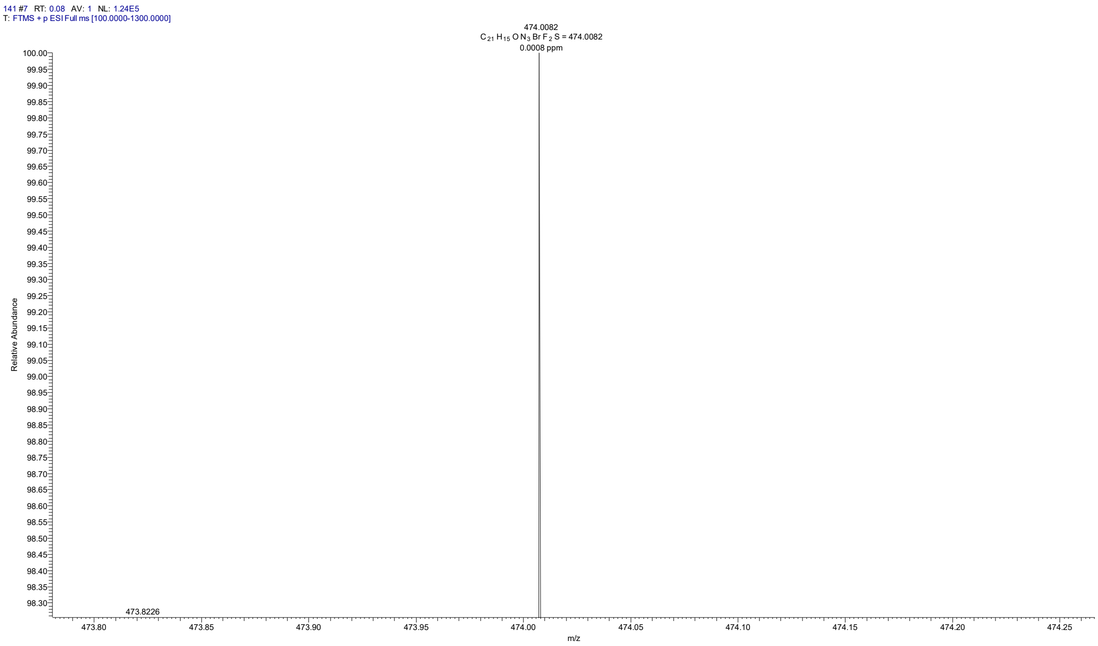

Figure S33. The HRMS spectrum of **5k**.

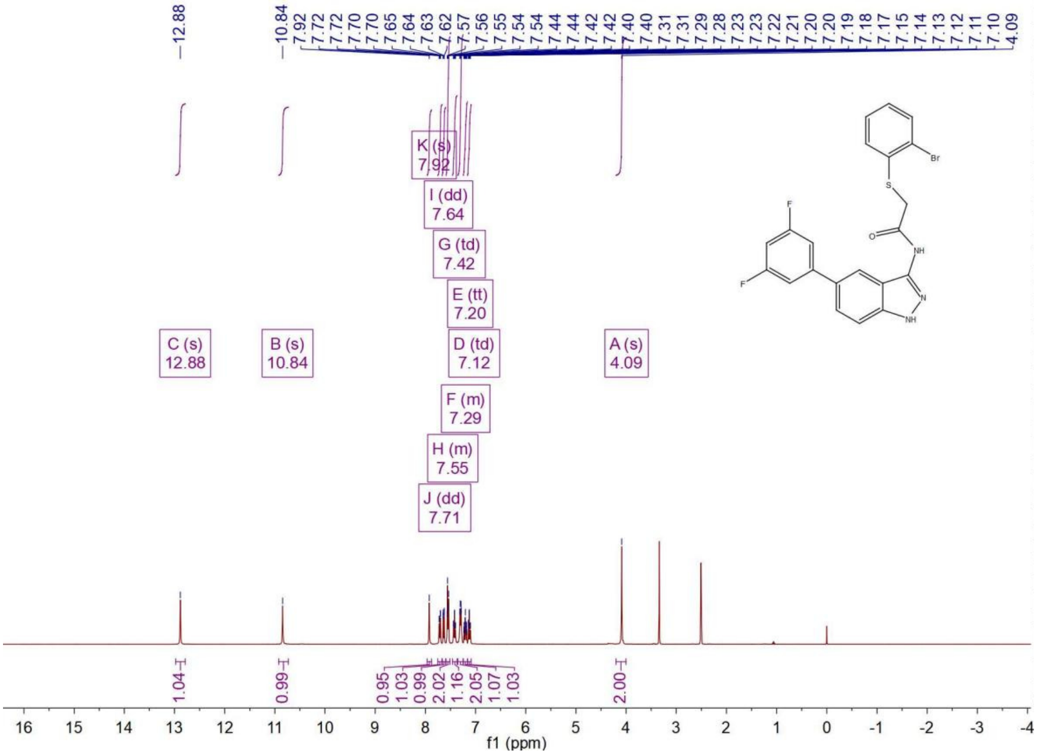

Figure S34. The ^{1}H NMR spectrum of **5l**.

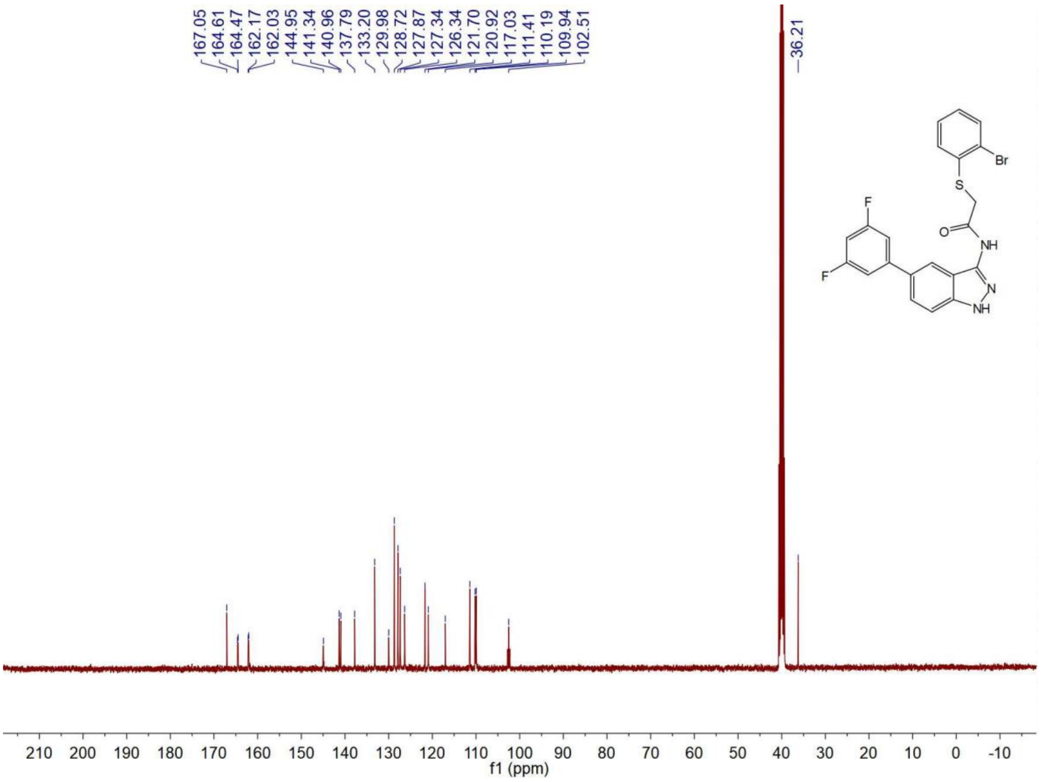

Figure S35. The ^{13}C NMR spectrum of **5l**.

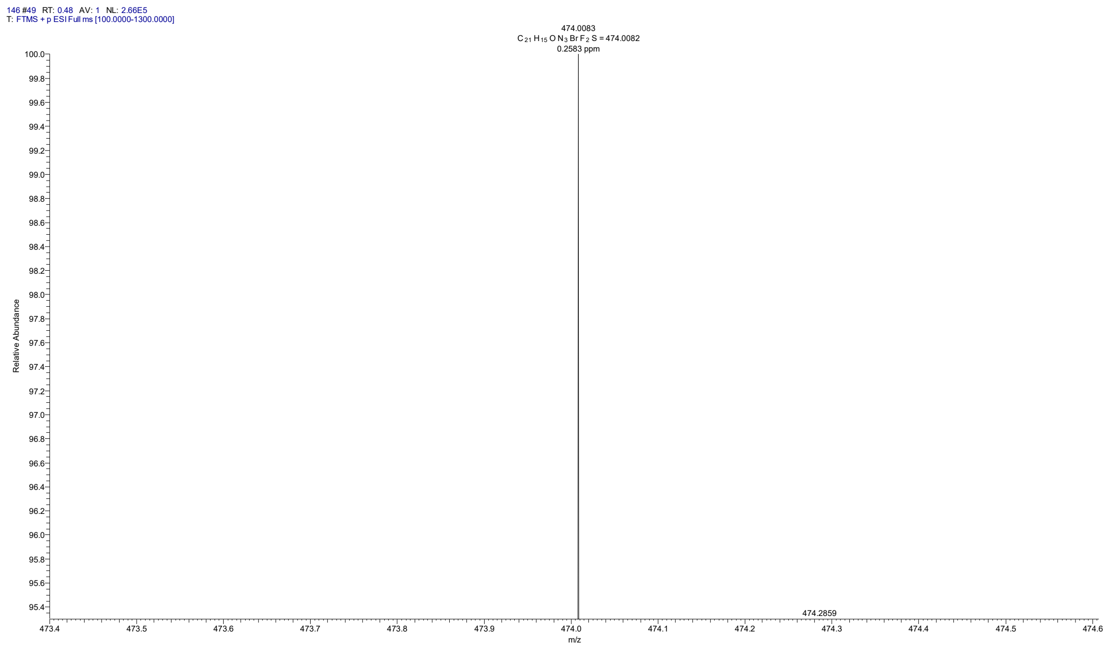

Figure S36. The HRMS spectrum of **5l**.

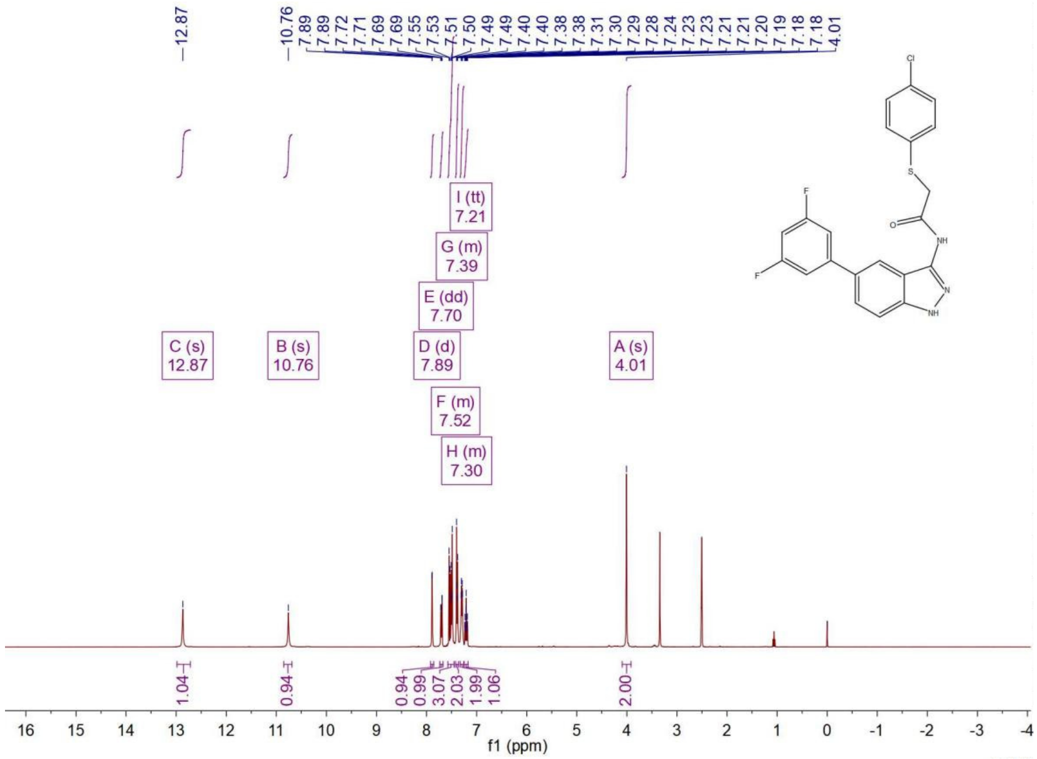

Figure S37. The ^{1}H NMR spectrum of **5m**.

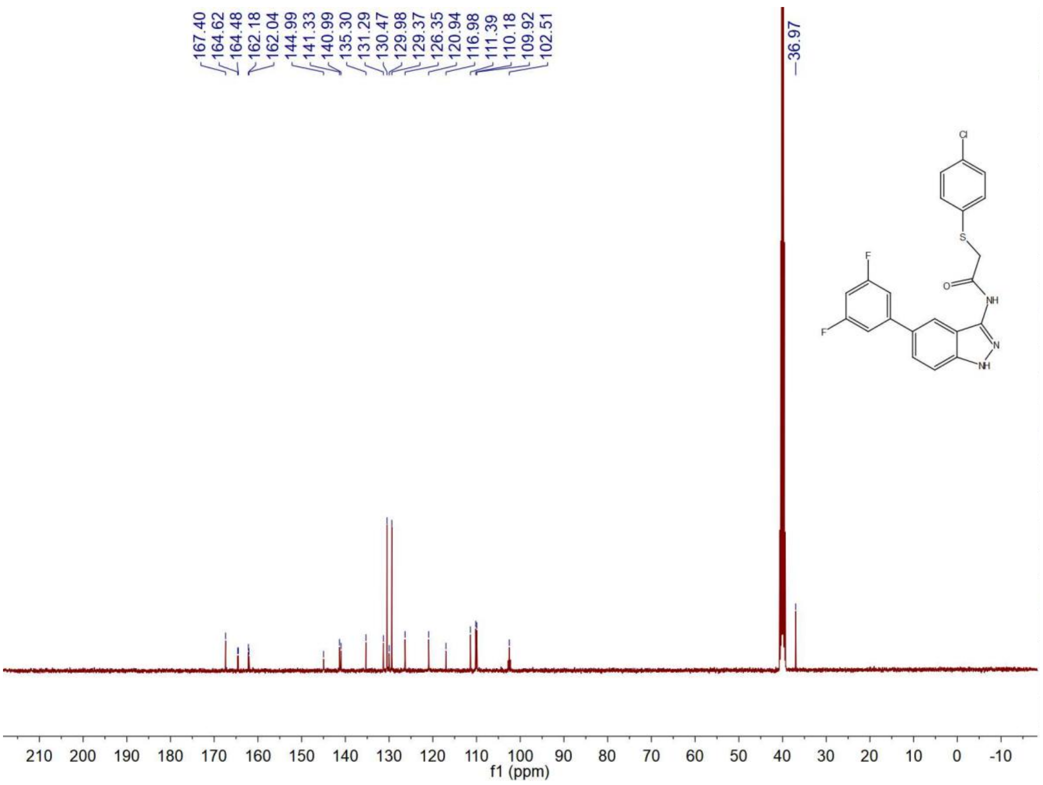

Figure S38. The ^{13}C NMR spectrum of **5m**.

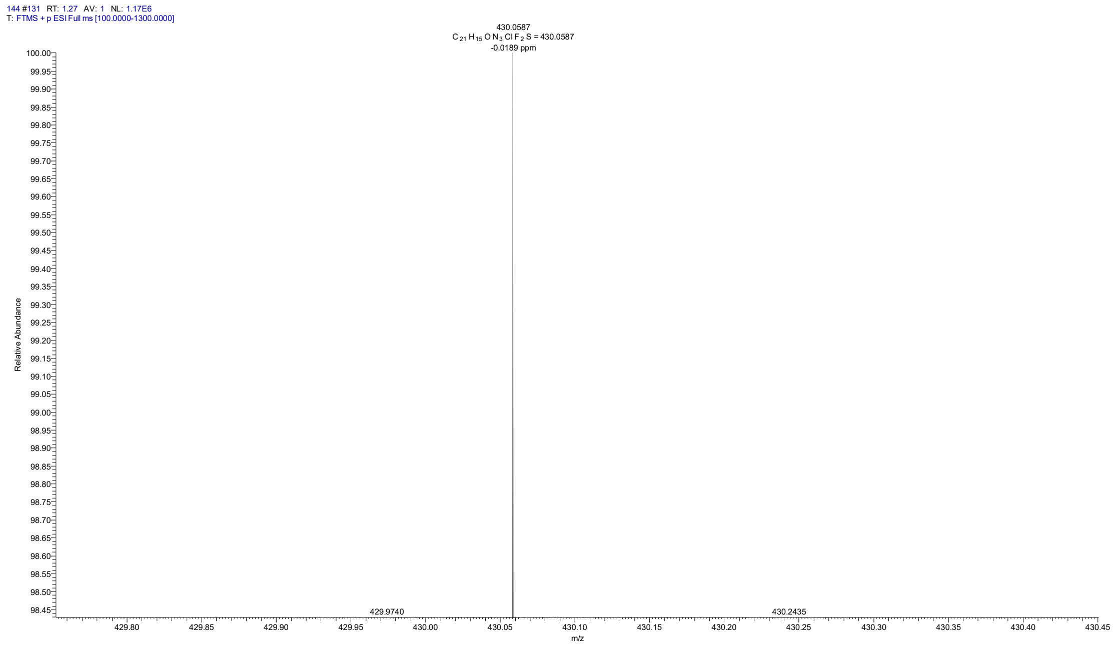

Figure S39. The HRMS spectrum of **5m**.

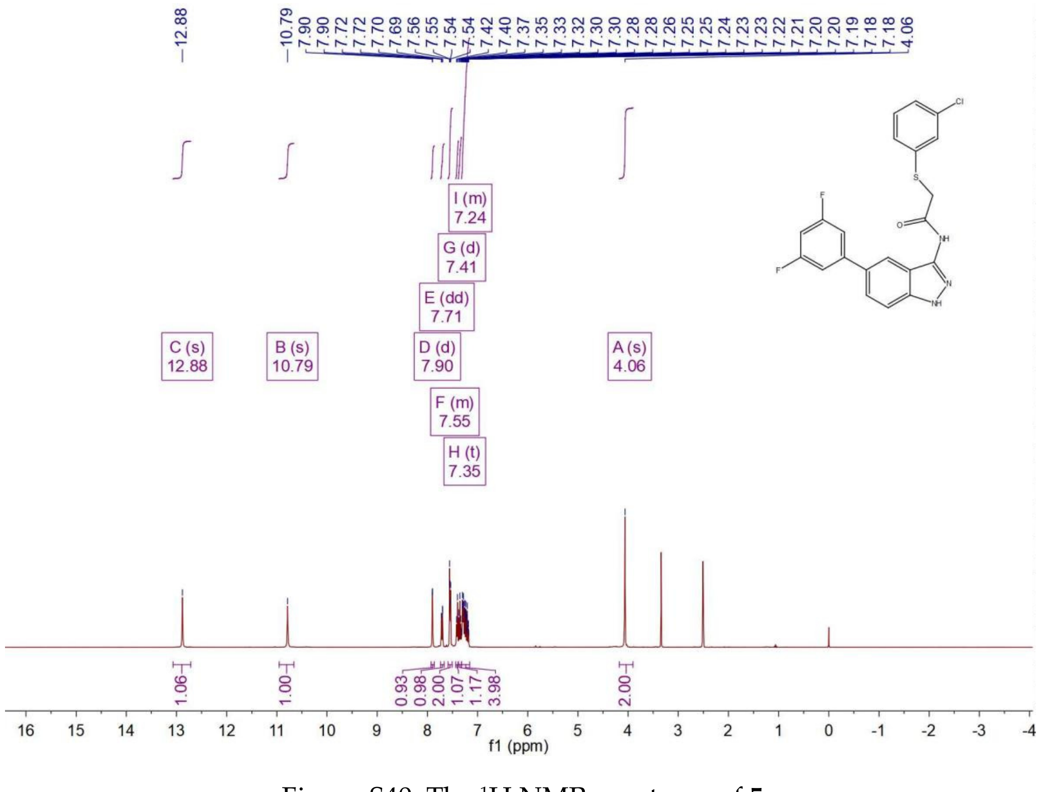

Figure S40. The ^{1}H NMR spectrum of **5n**.

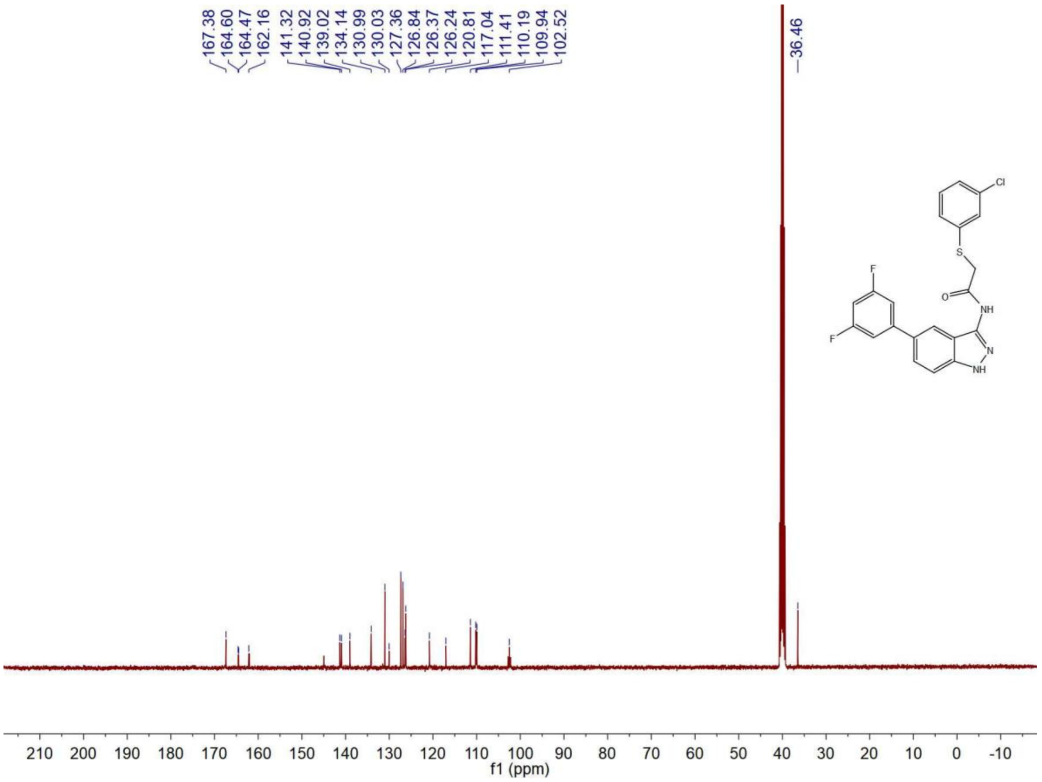

Figure S41. The ^{13}C NMR spectrum of **5n**.

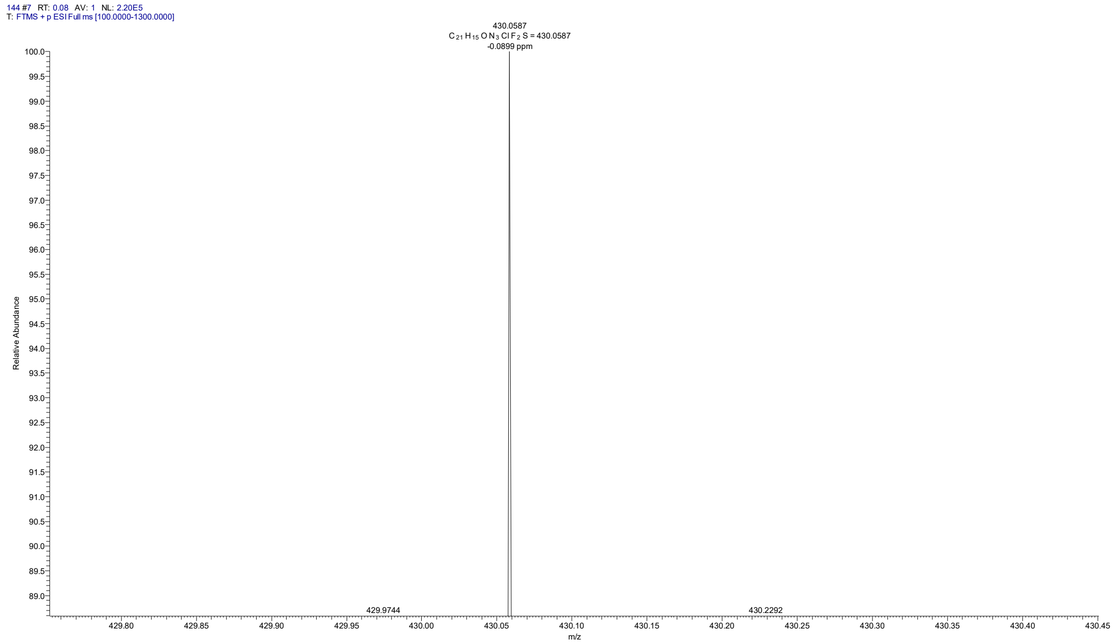

Figure S42. The HRMS spectrum of **5n**.

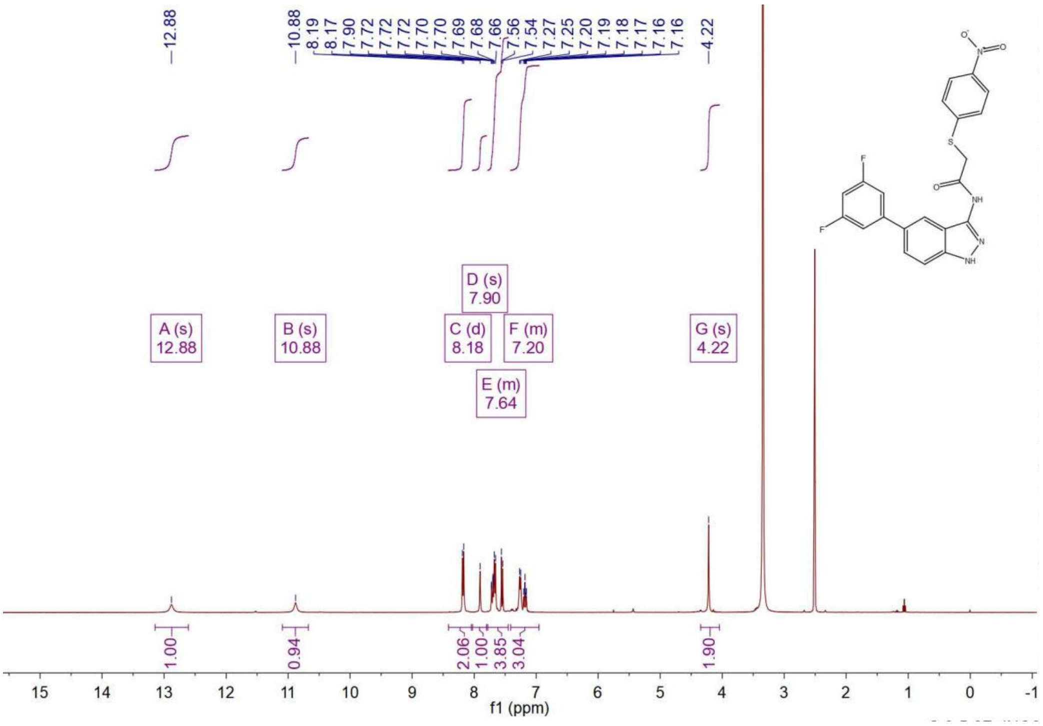

Figure S43. The ^{1}H NMR spectrum of **5o**.

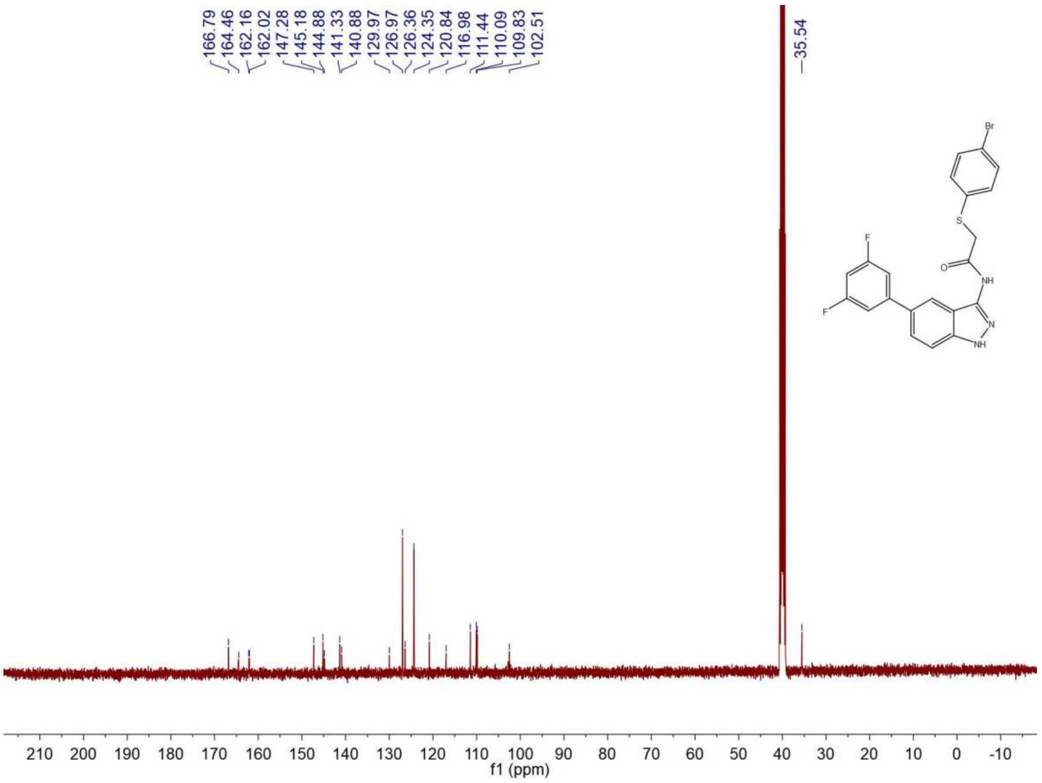

Figure S44. The ^{13}C NMR spectrum of **5o**.

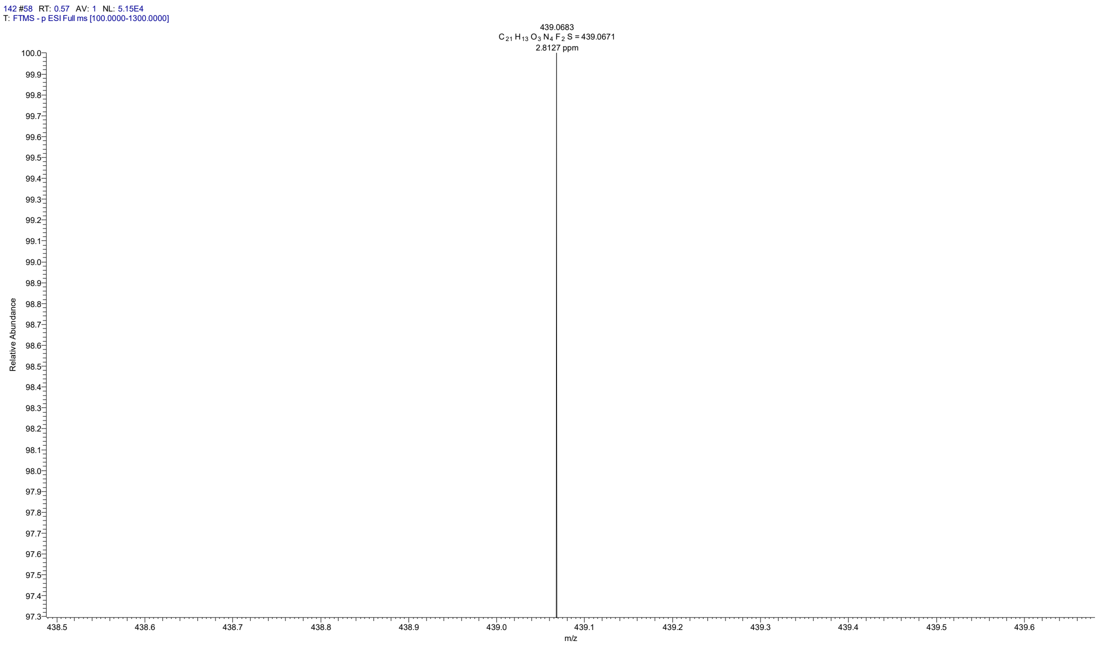

Figure S45. The HRMS spectrum of **5o**.

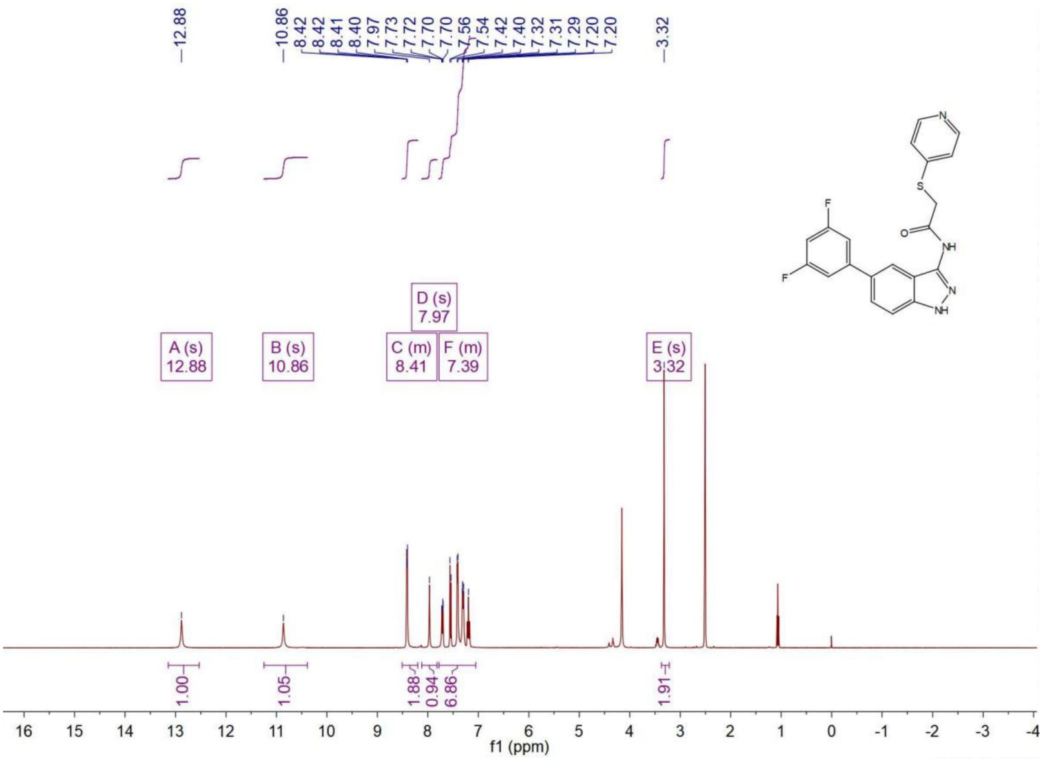

Figure S46. The ^{1}H NMR spectrum of **5p**.

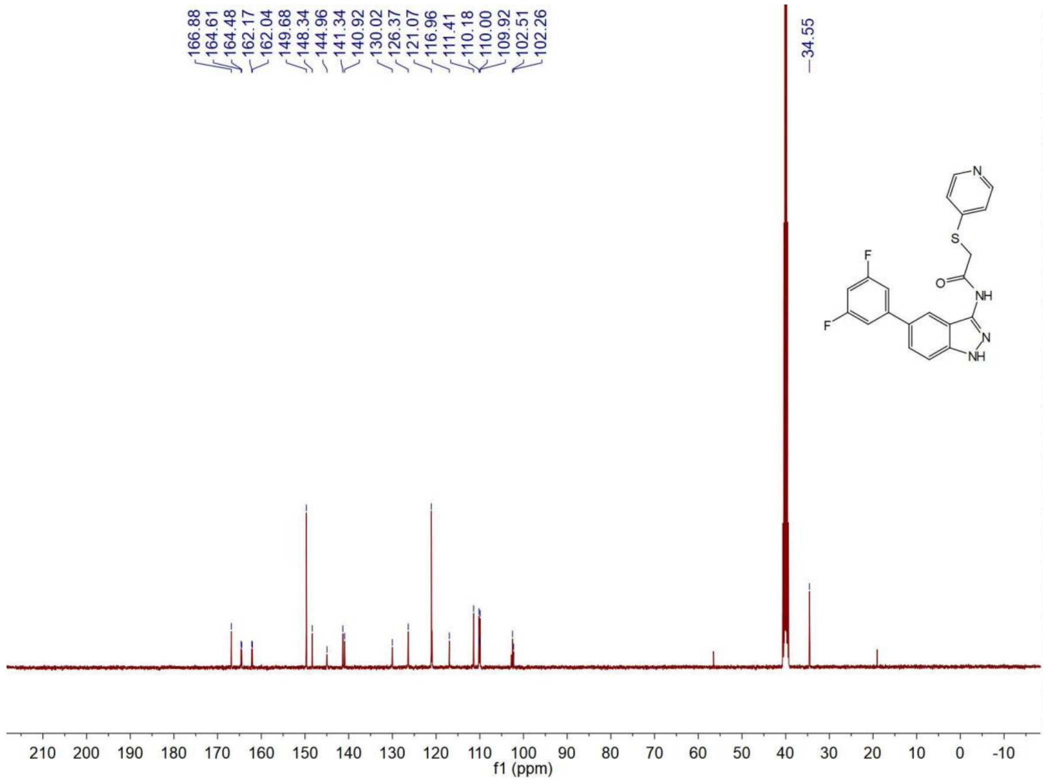

Figure S47. The ^{13}C NMR spectrum of **5p**.

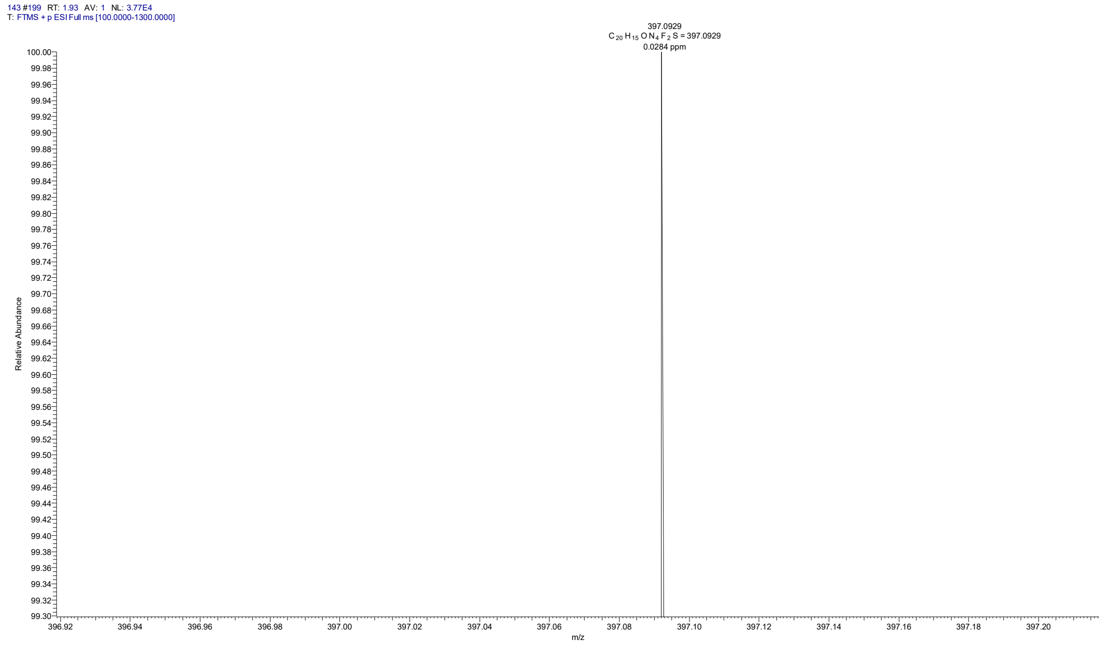

Figure S48. The HRMS spectrum of **5p**.

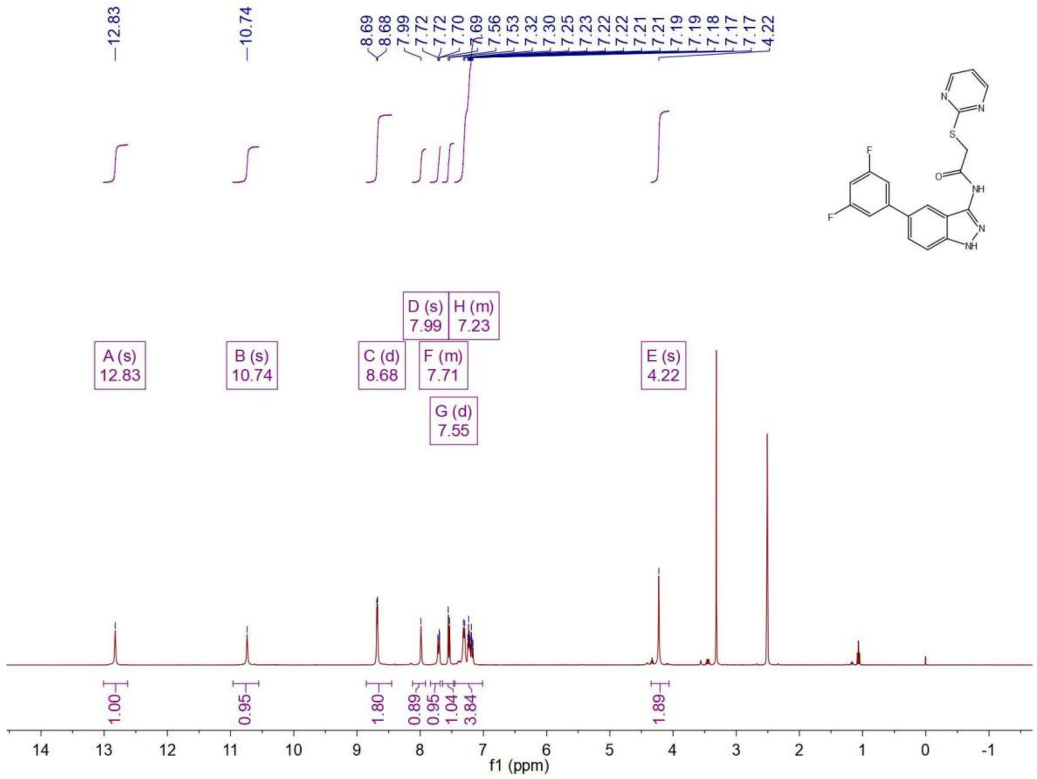

Figure S49. The ^{1}H NMR spectrum of **5q**.

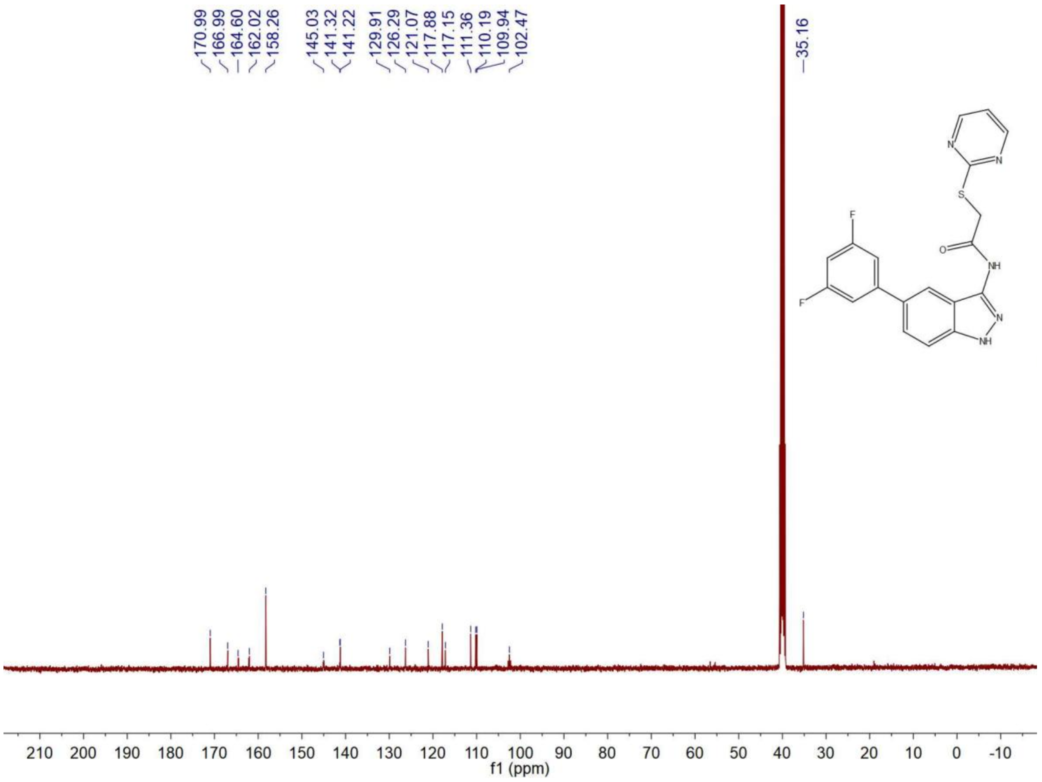

Figure S50. The ^{13}C NMR spectrum of **5q**.

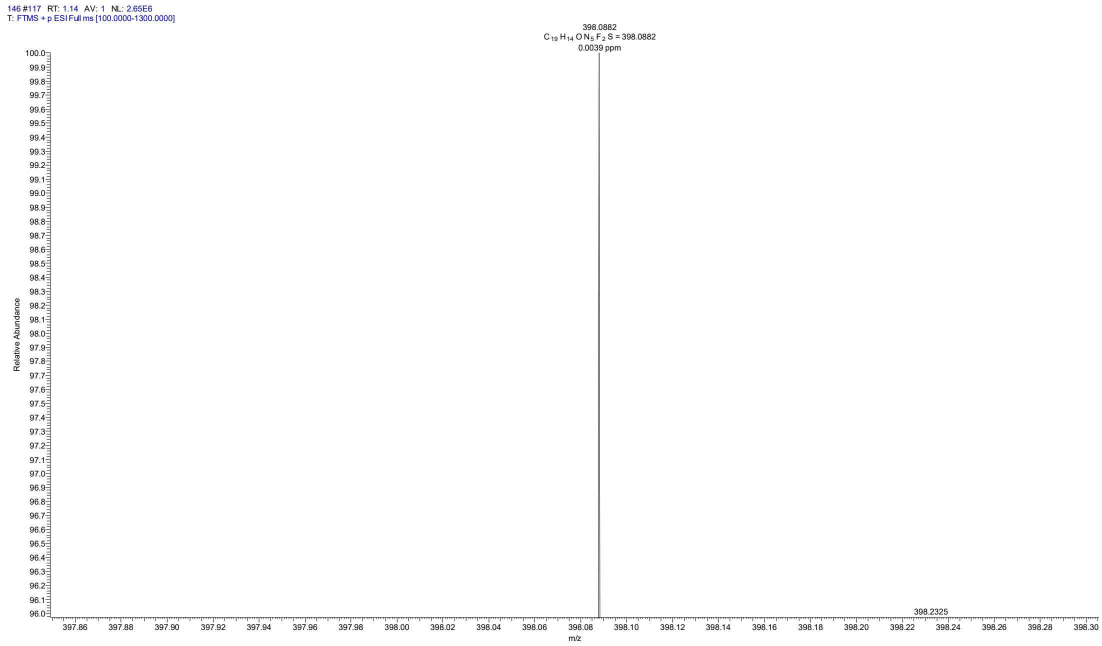

Figure S51. The HRMS spectrum of **5q**.

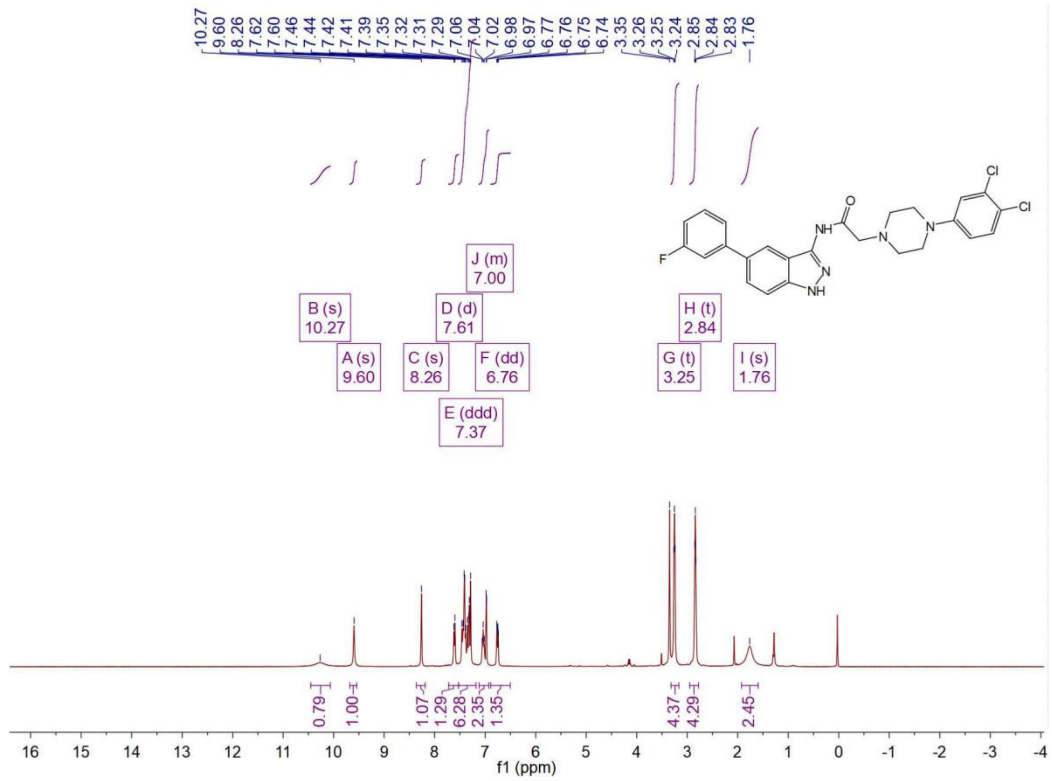

Figure S52. The ^{1}H NMR spectrum of **6a**.

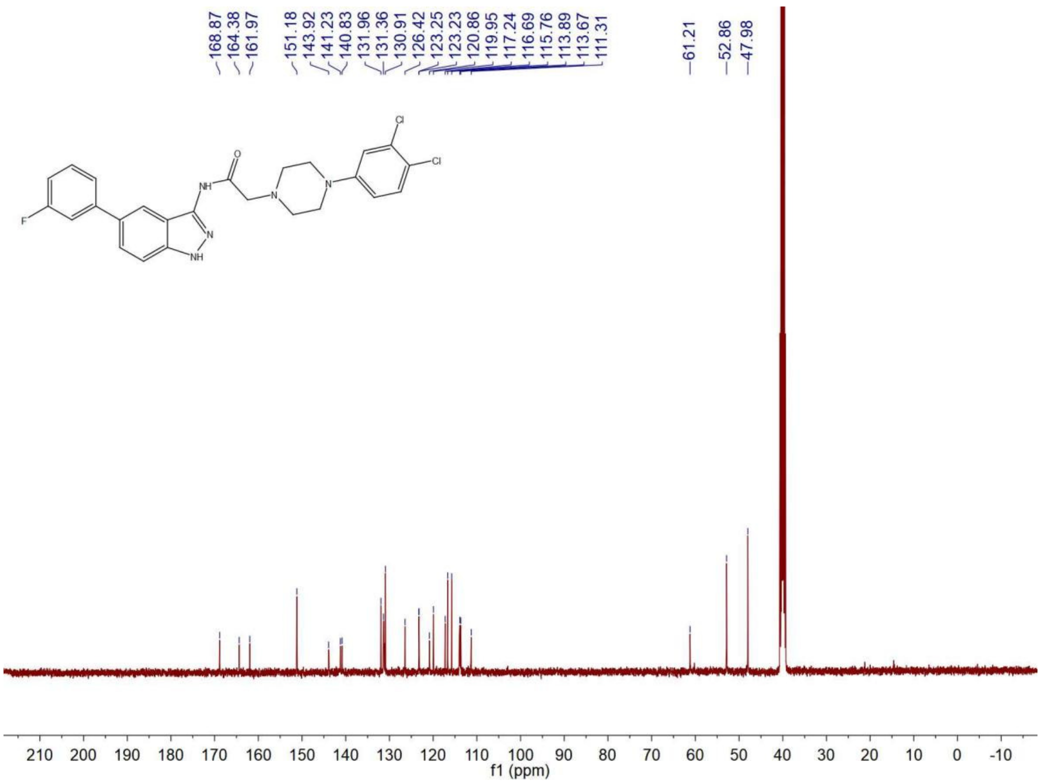

Figure S53. The ^{13}C NMR spectrum of **6a**.

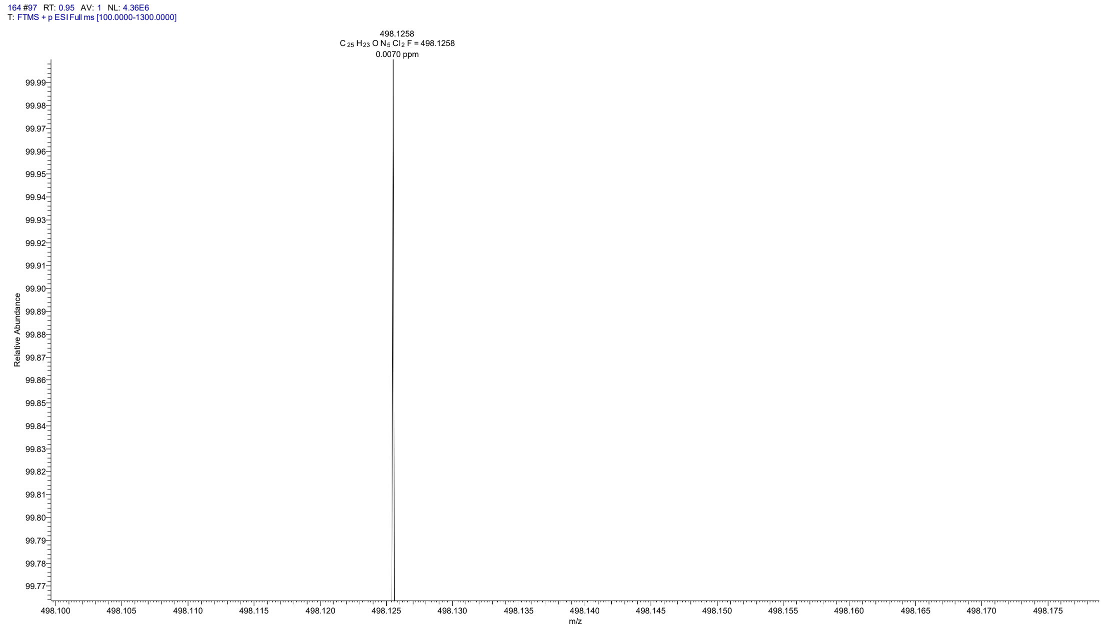

Figure S54. The HRMS spectrum of **6a**.

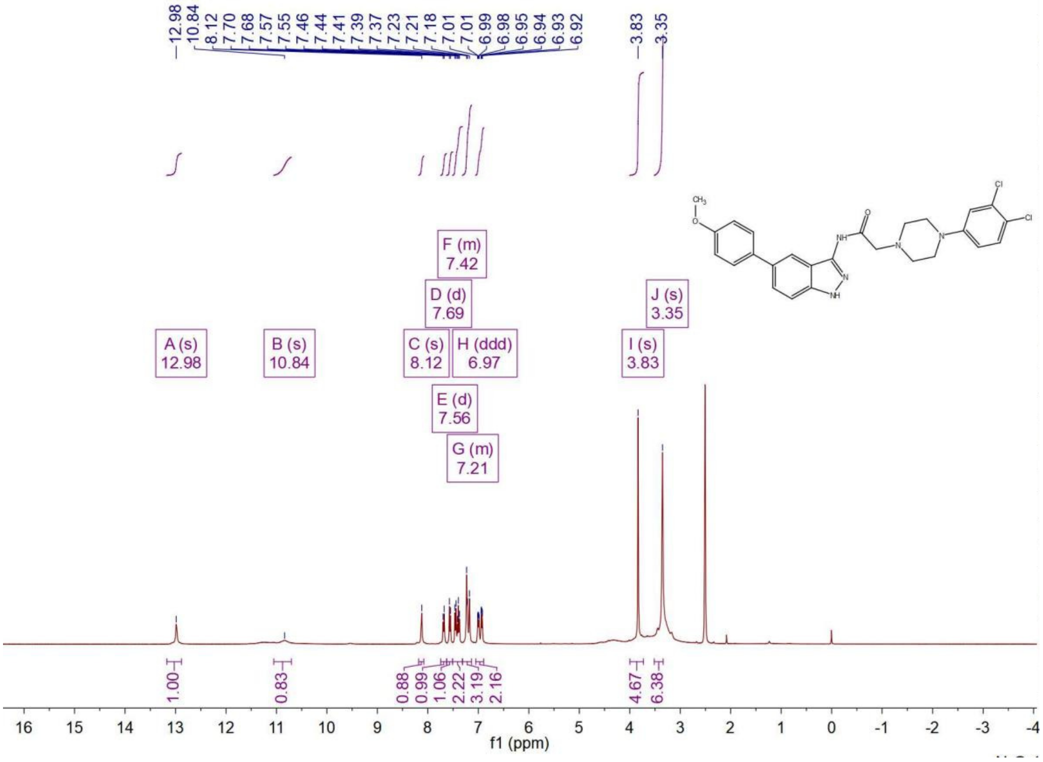

Figure S55. The ^{1}H NMR spectrum of **6b**.

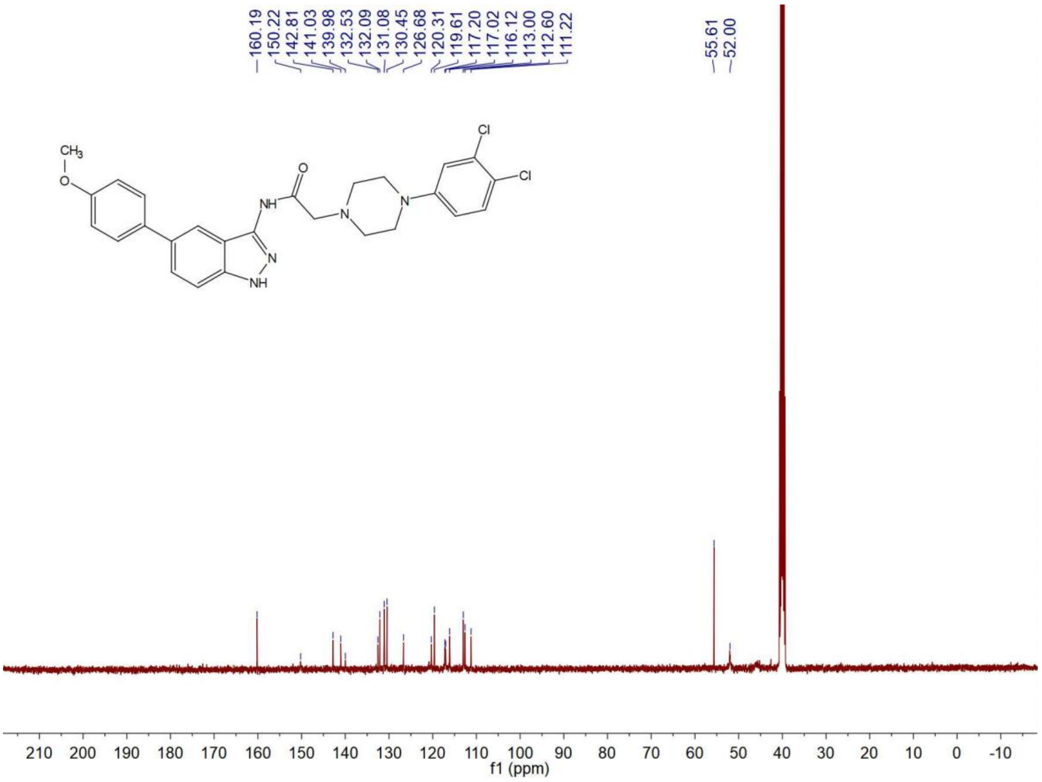

Figure S56. The ^{13}C NMR spectrum of **6b**.

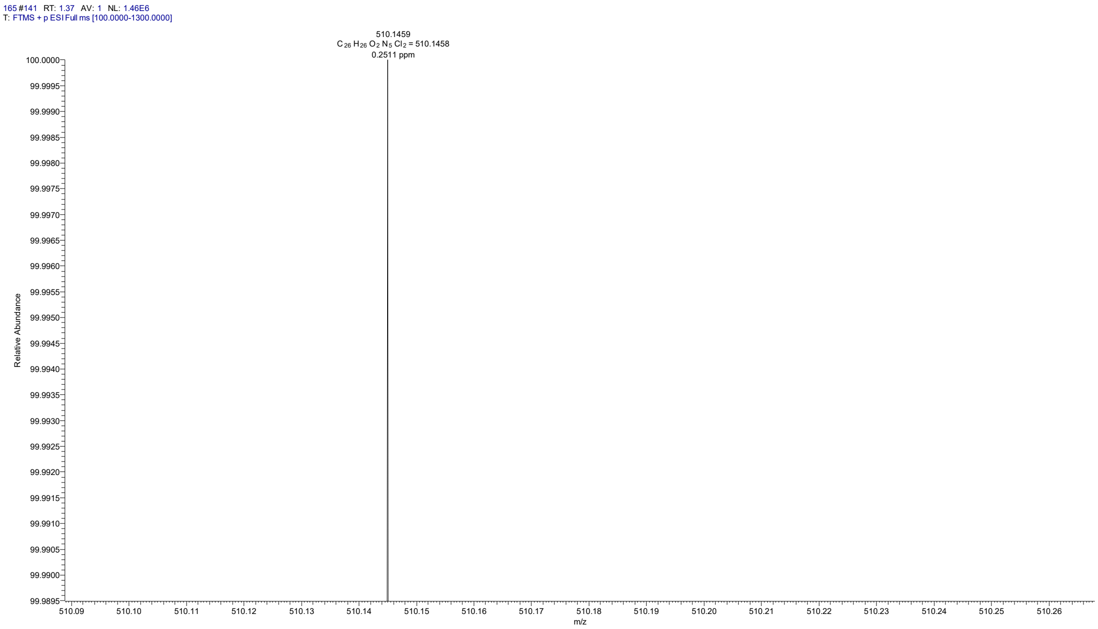

Figure S57. The HRMS spectrum of **6b**.

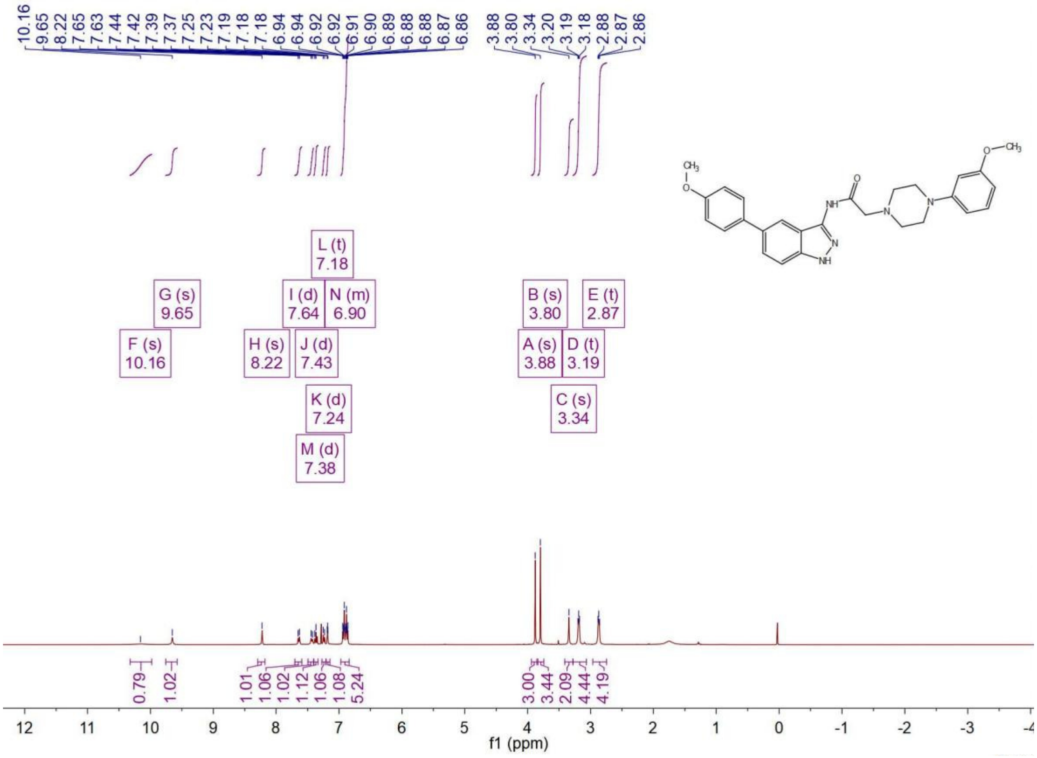

Figure S58. The ^{1}H NMR spectrum of **6c**.

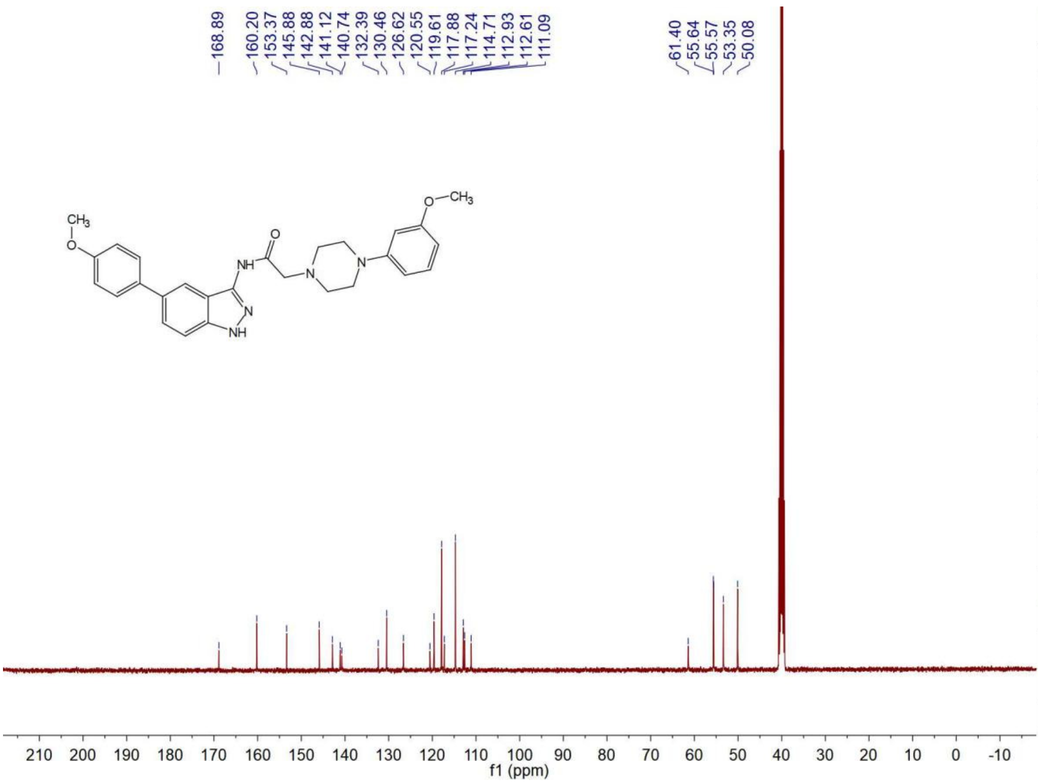

Figure S59. The ^{13}C NMR spectrum of **6c**.

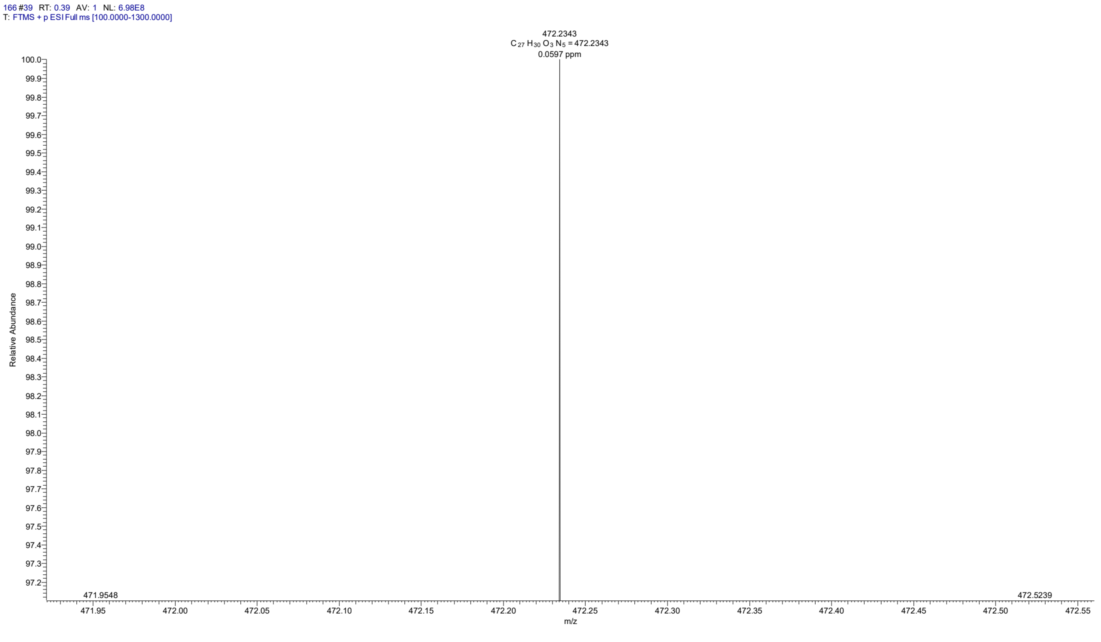

Figure S60. The HRMS spectrum of **6c**.

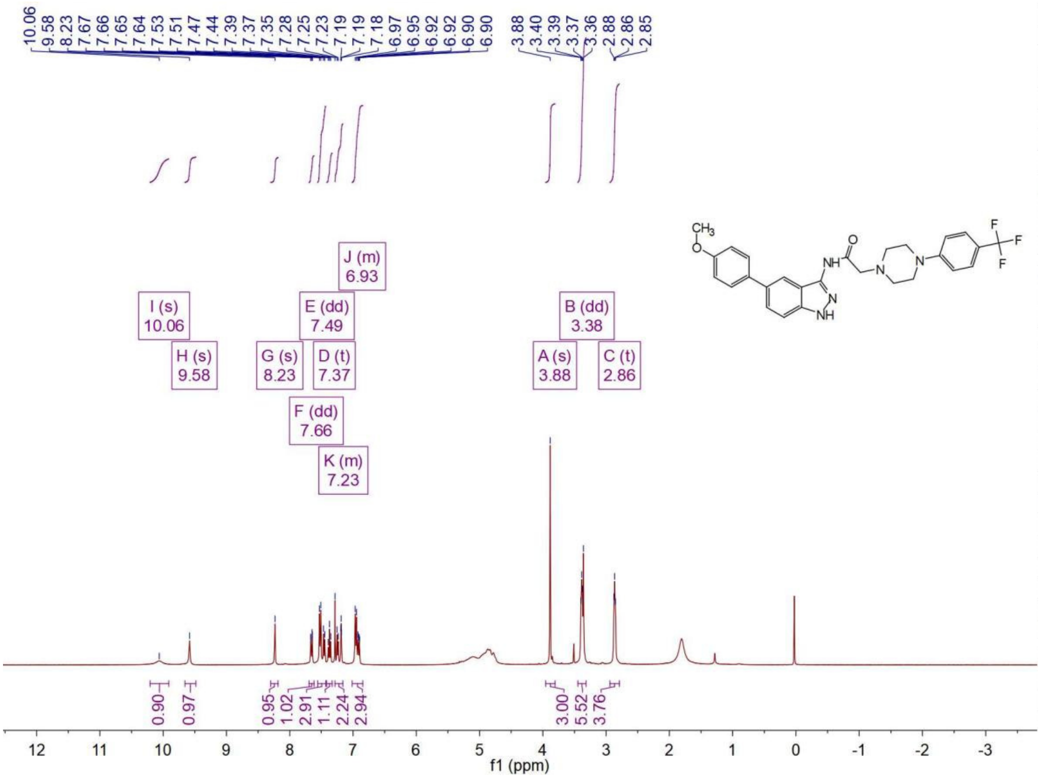

Figure S61. The ^{1}H NMR spectrum of **6d**.

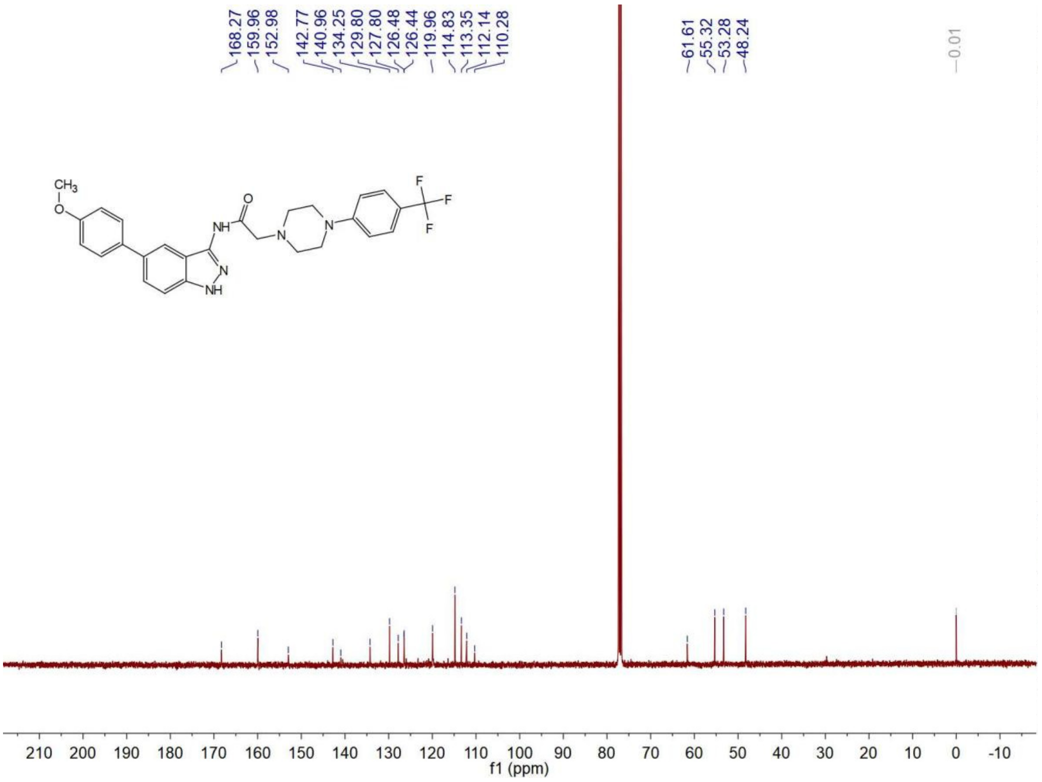

Figure S62. The ^{13}C NMR spectrum of **6d**.

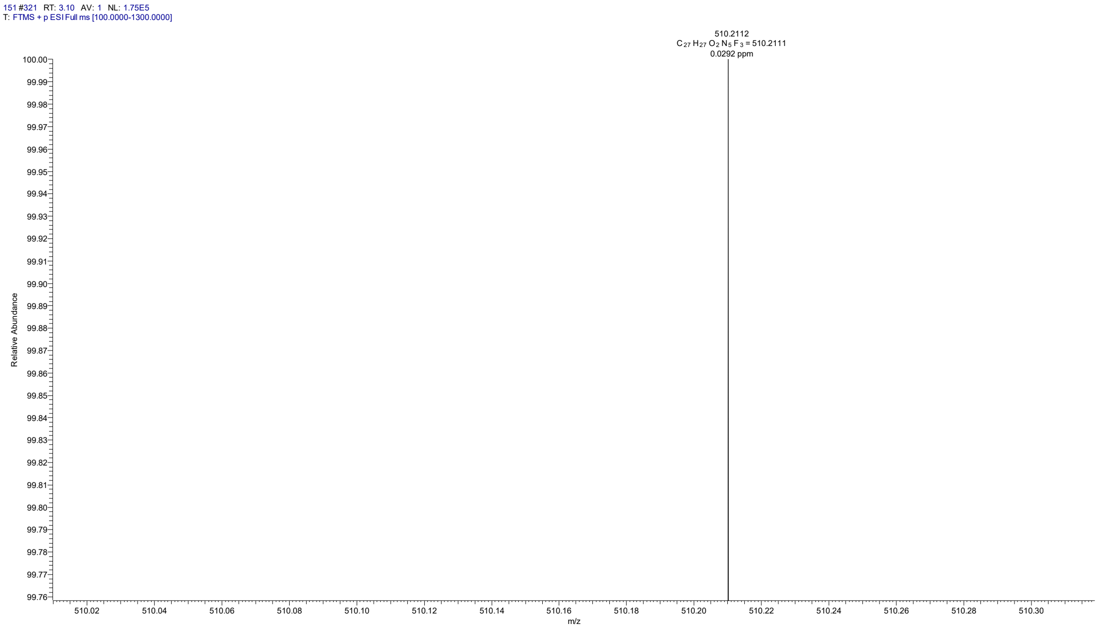

Figure S63. The HRMS spectrum of **6d**.

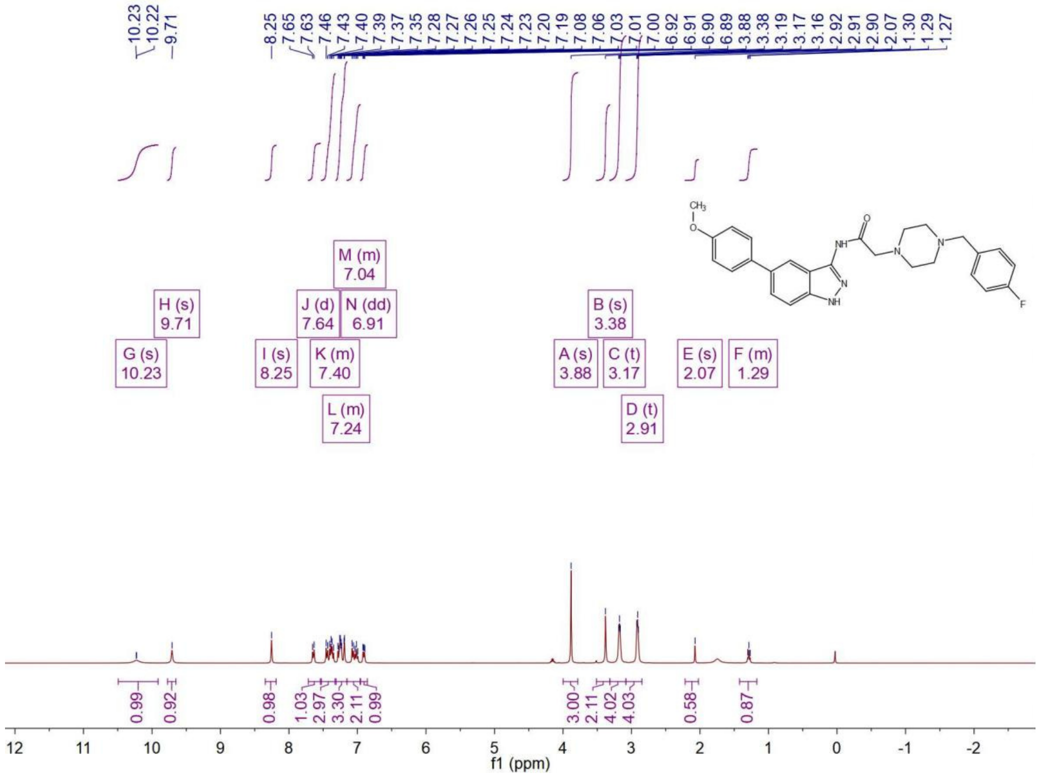

Figure S64. The ^{1}H NMR spectrum of **6e**.

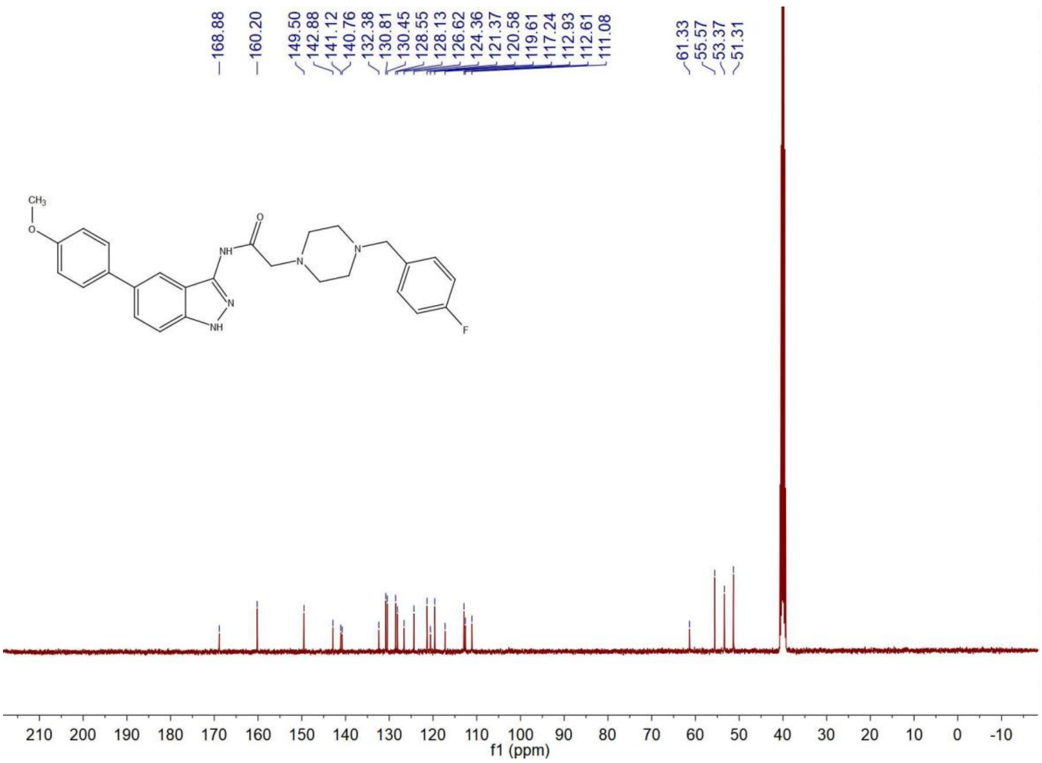

Figure S65. The ^{13}C NMR spectrum of **6e**.

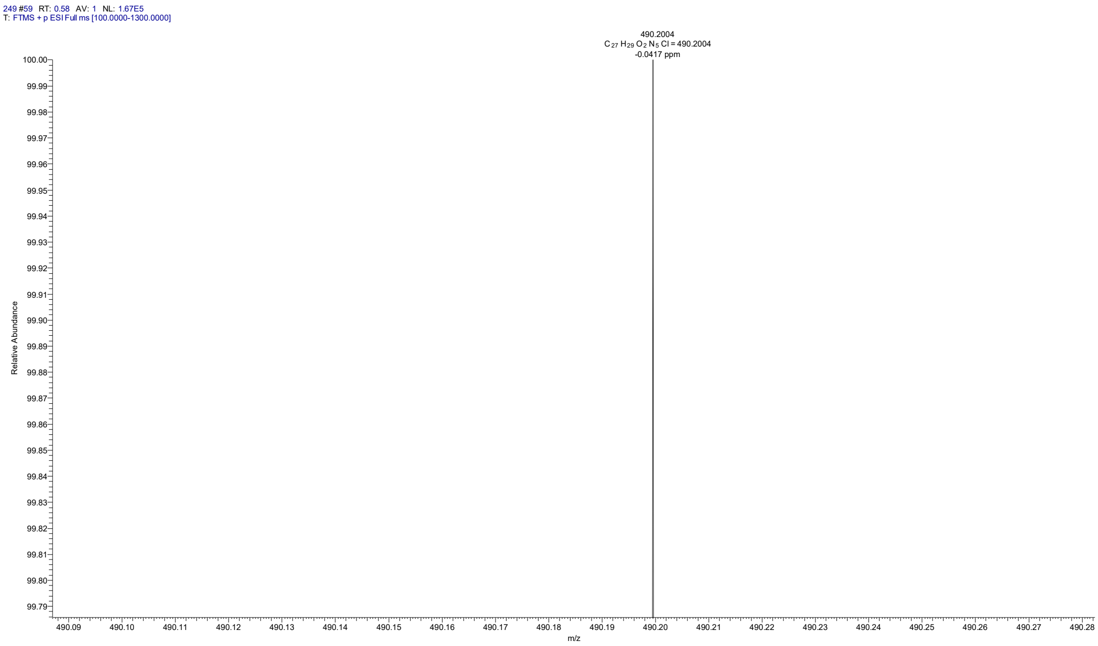

Figure S66. The HRMS spectrum of **6e**.

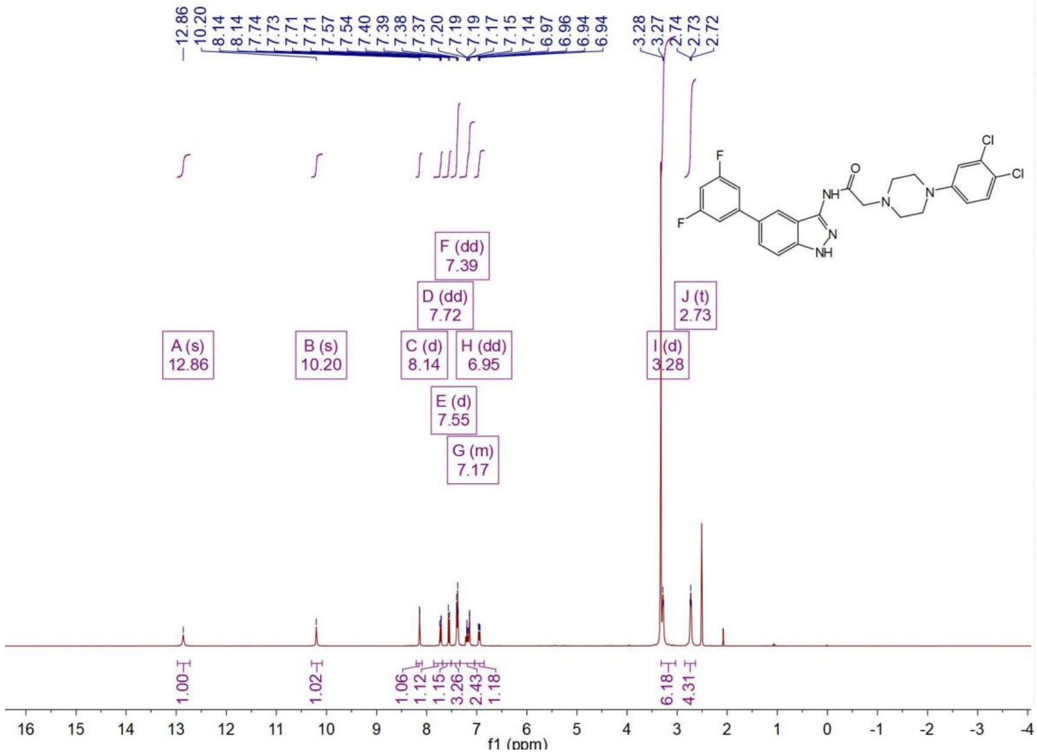

Figure S67. The ^{1}H NMR spectrum of **6f**.

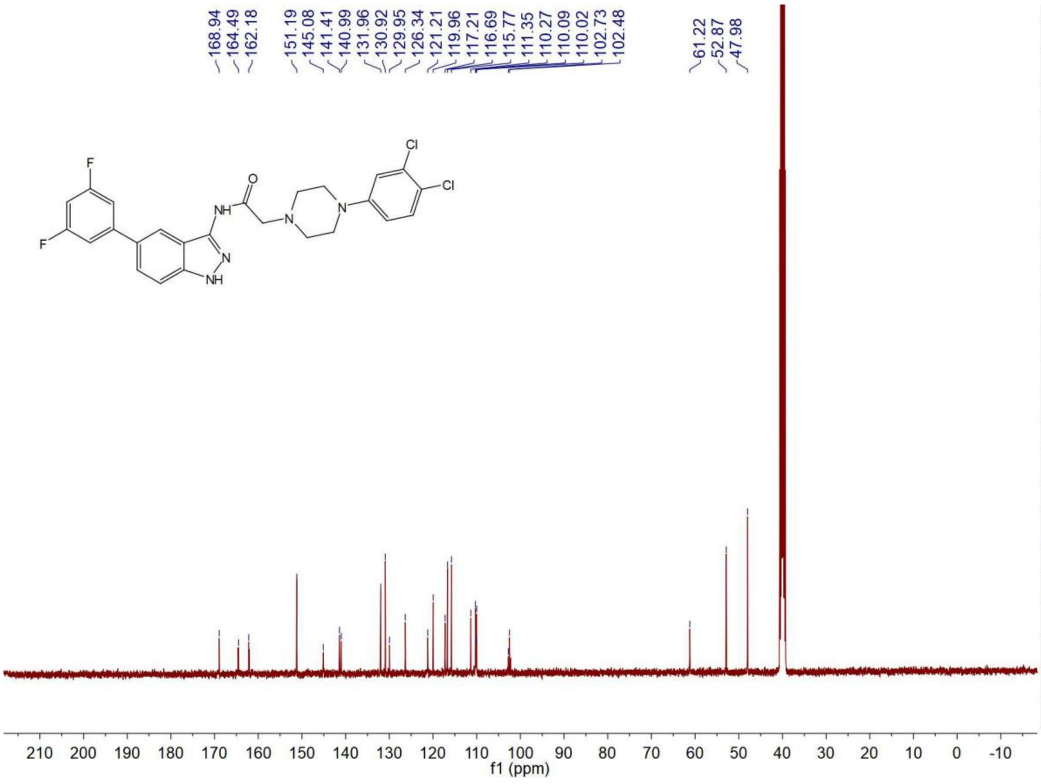

Figure S68. The ^{13}C NMR spectrum of **6f**.

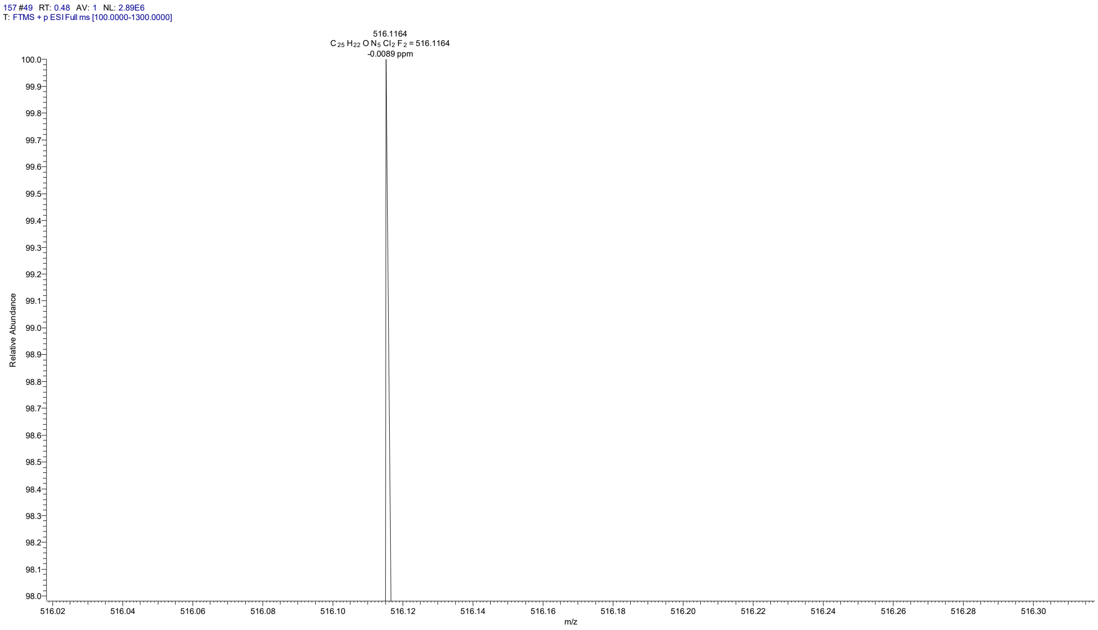

Figure S69. The HRMS spectrum of **6f**.

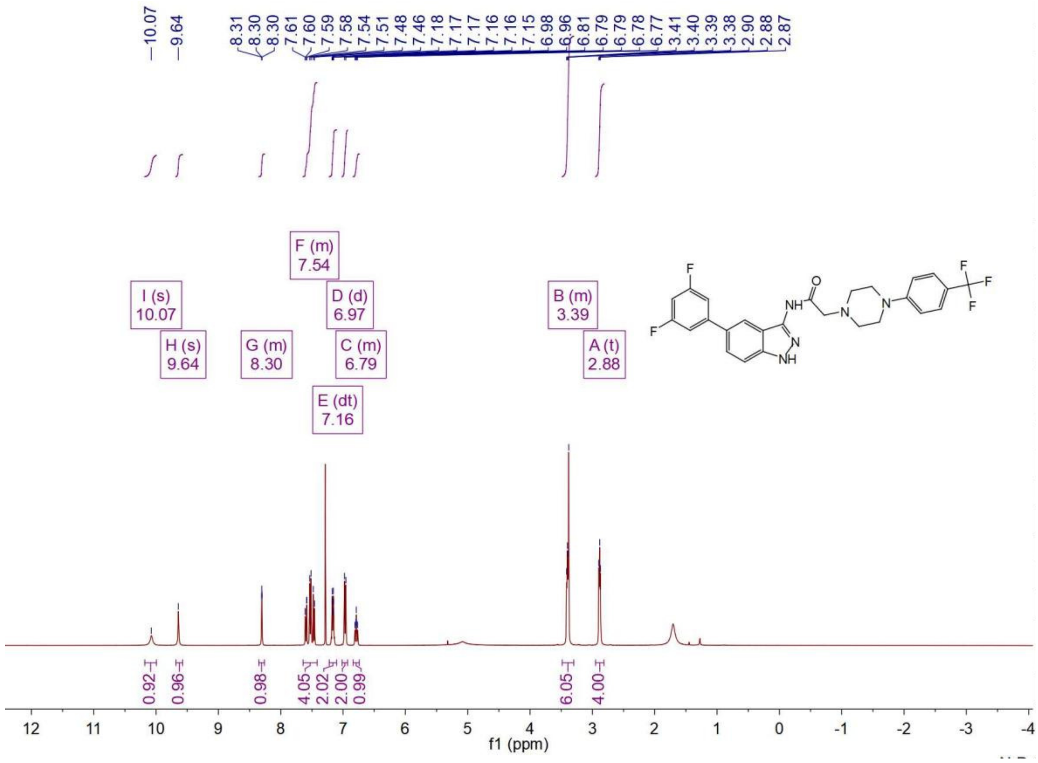

Figure S70. The ^{1}H NMR spectrum of **6g**.

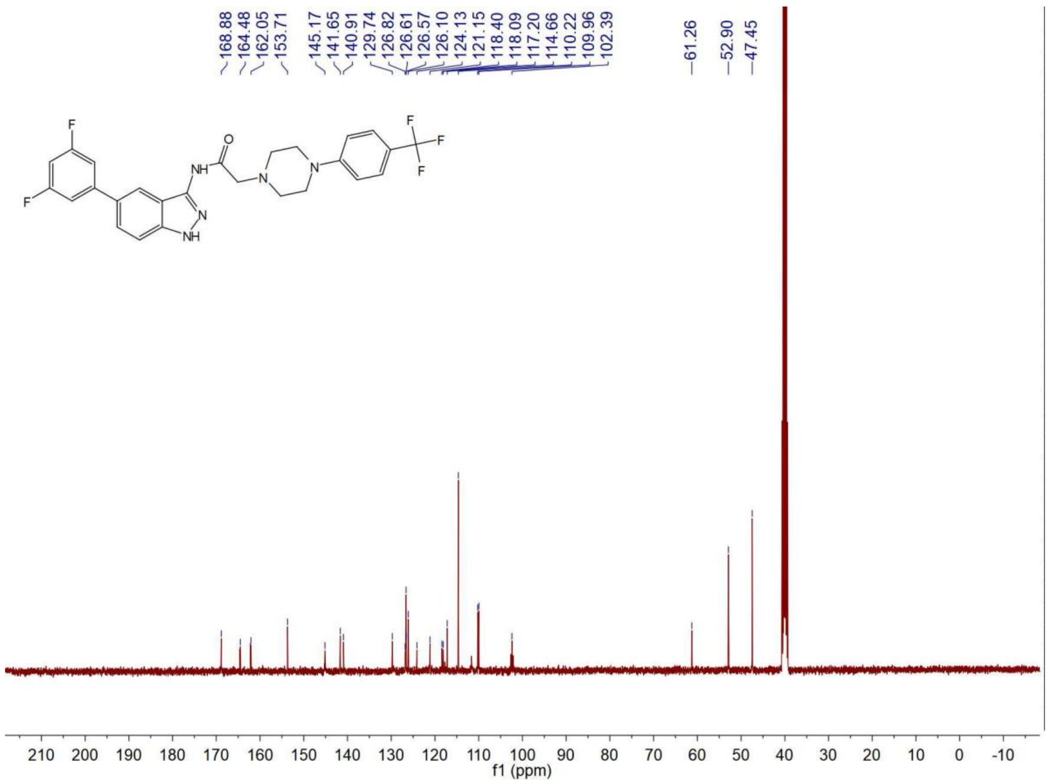

Figure S71. The ^{13}C NMR spectrum of **6g**.

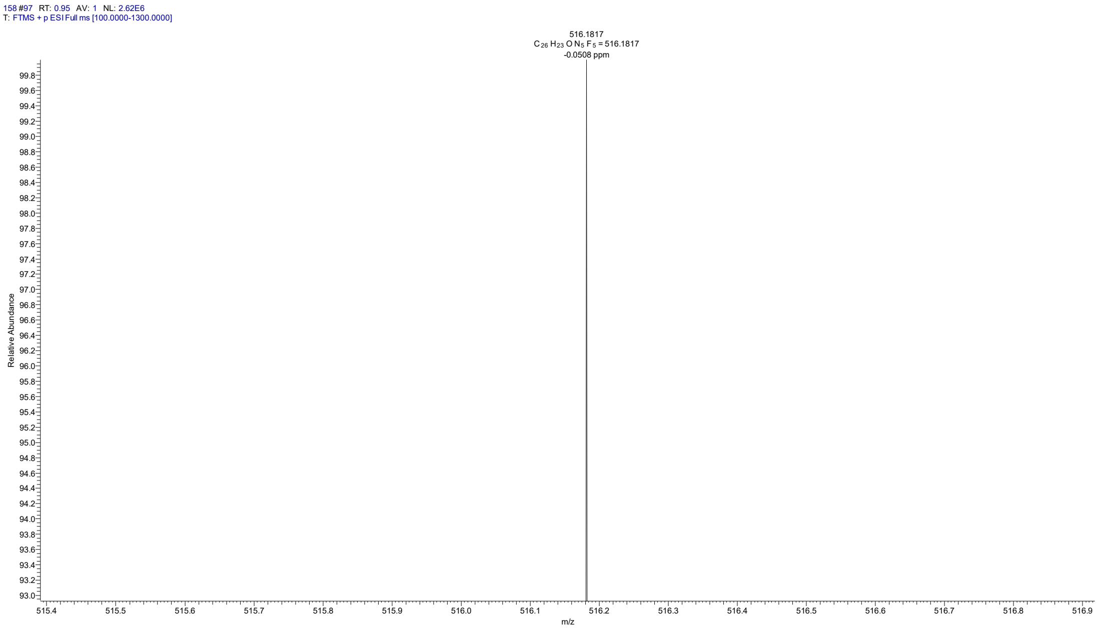

Figure S72. The HRMS spectrum of **6g**.

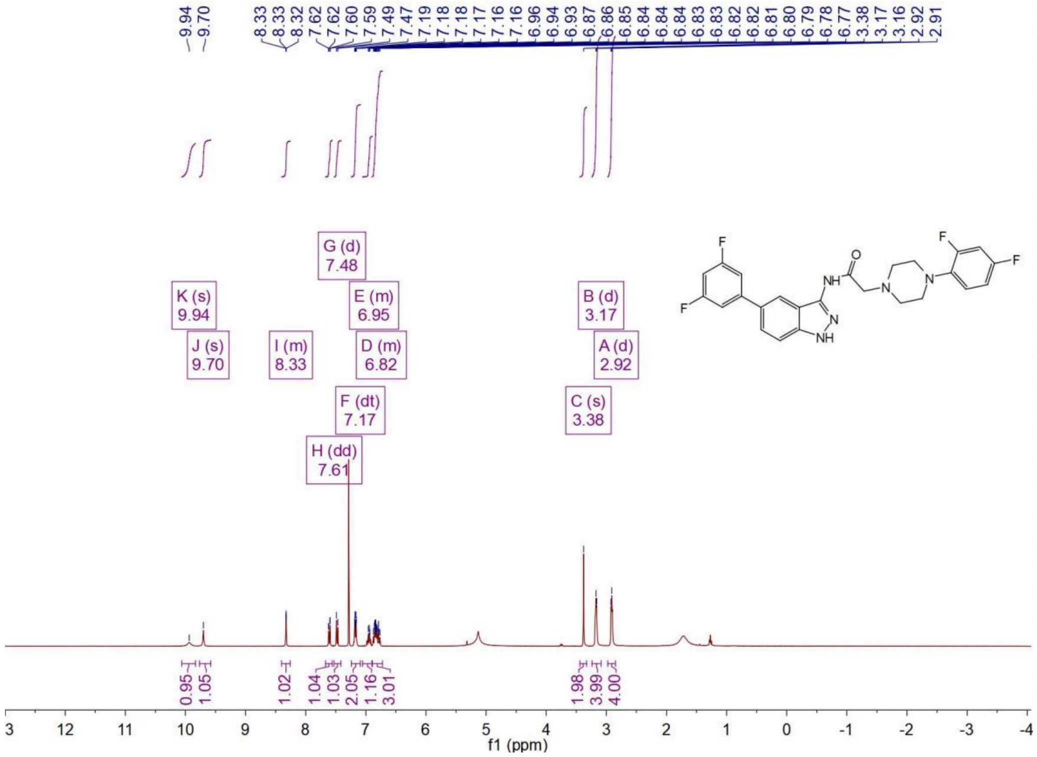

Figure S73. The ^{1}H NMR spectrum of **6h**.

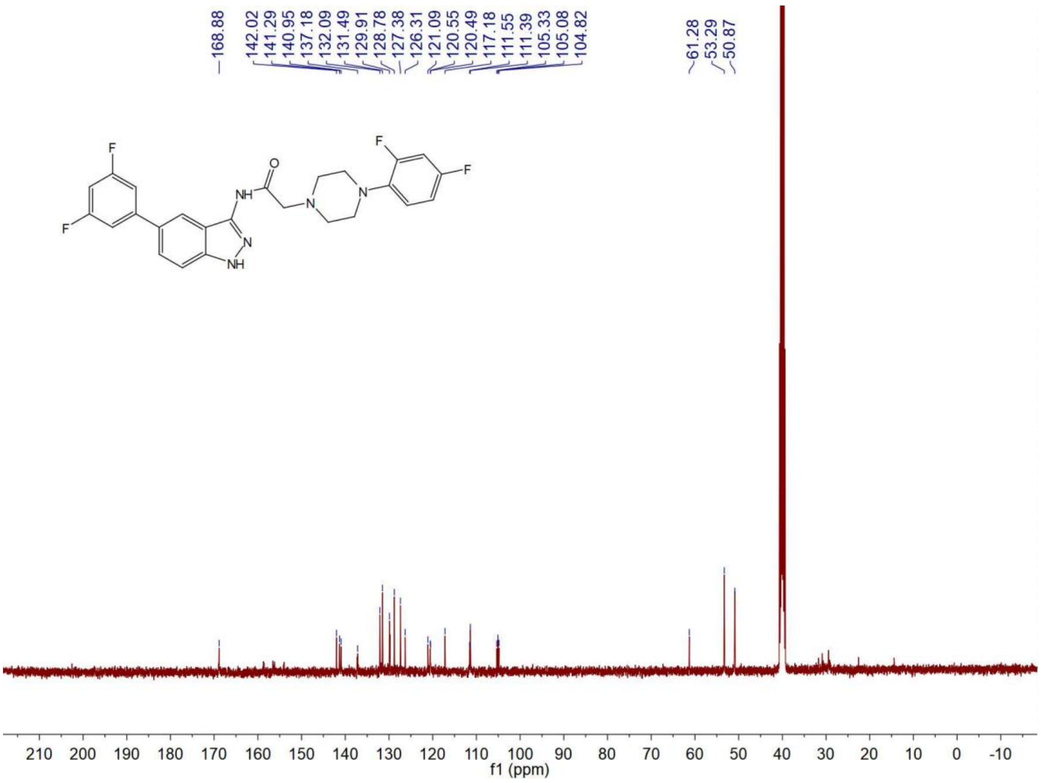

Figure S74. The ^{13}C NMR spectrum of **6h**.

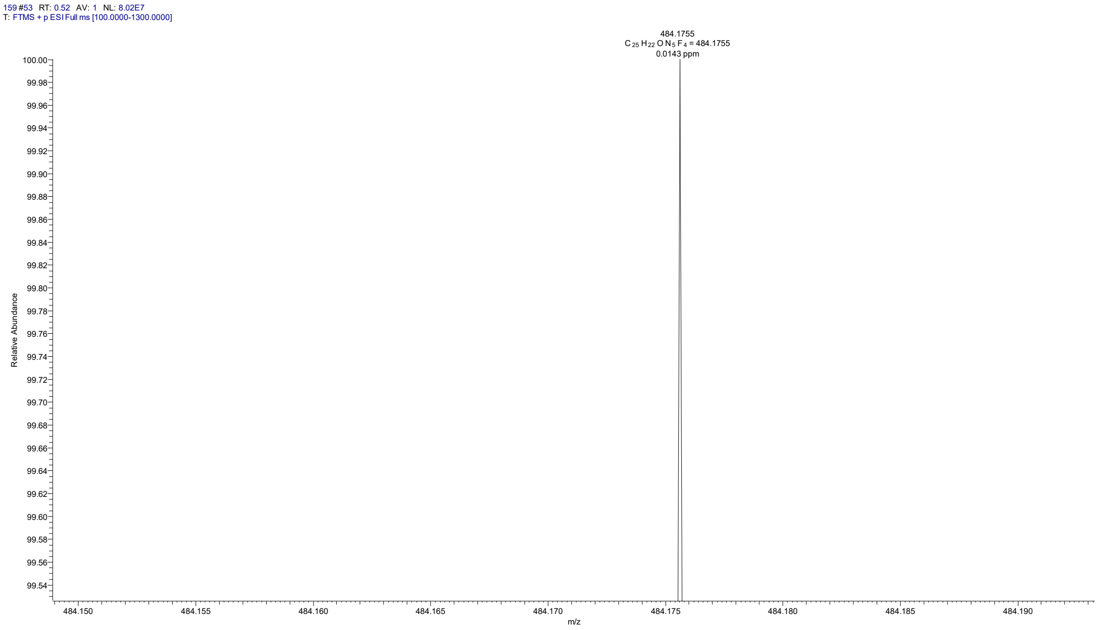

Figure S75. The HRMS spectrum of **6h**.

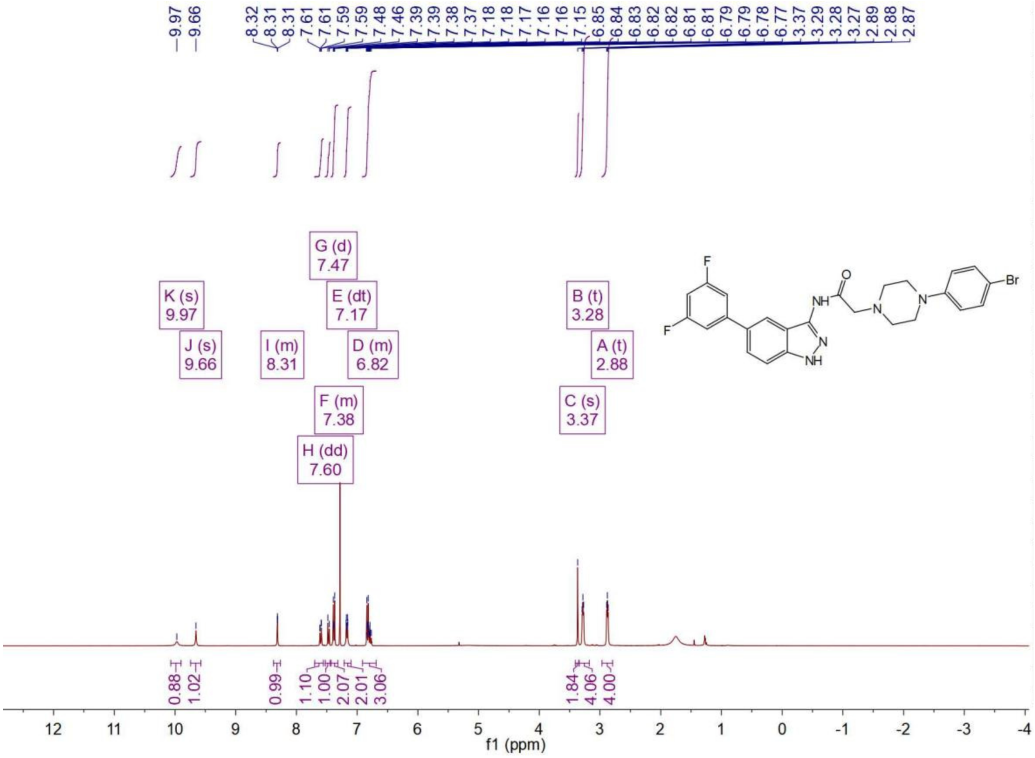

Figure S76. The ^{1}H NMR spectrum of **6i**.

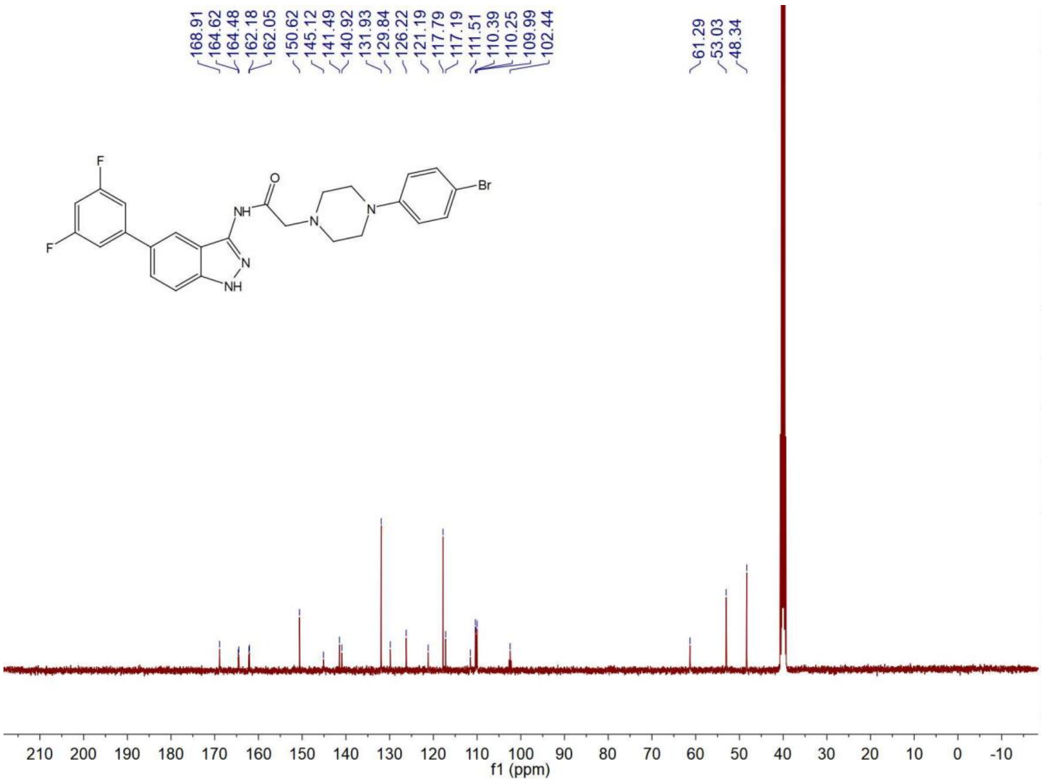

Figure S77. The ^{13}C NMR spectrum of **6i**.

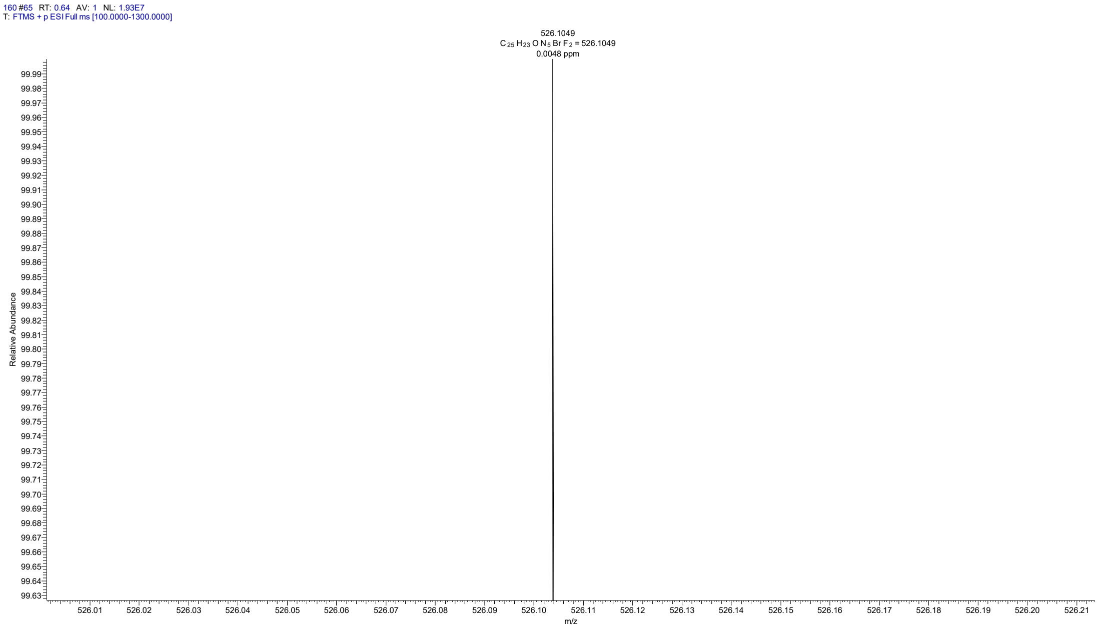

Figure S78. The HRMS spectrum of **6i**.

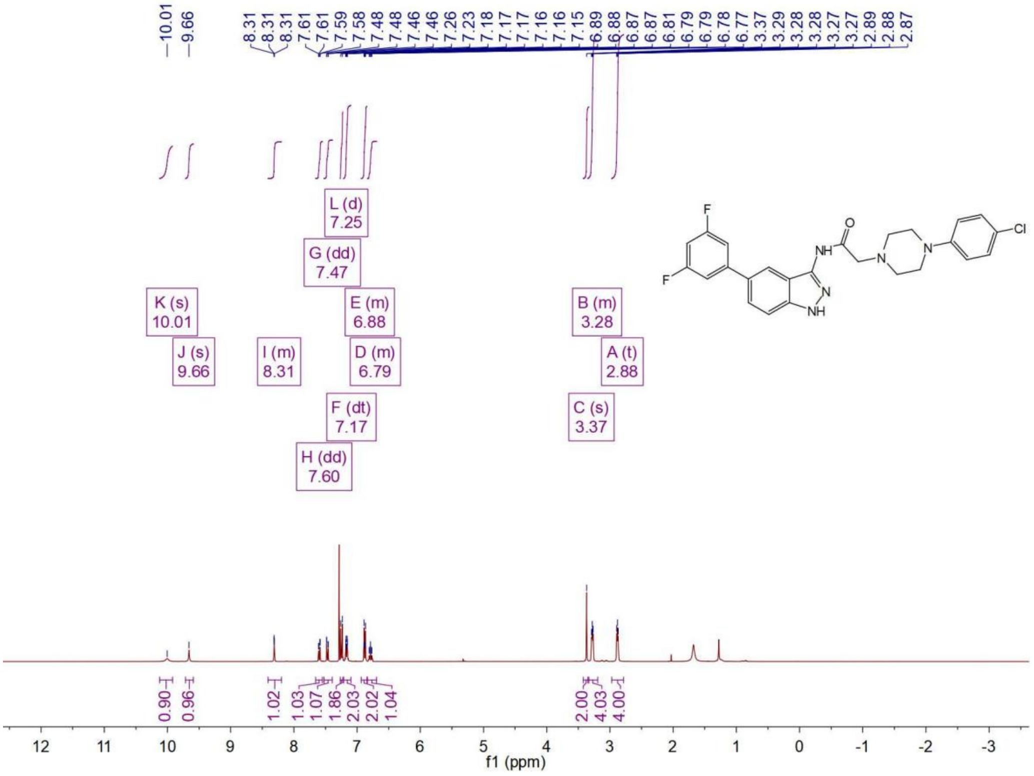

Figure S79. The ^{1}H NMR spectrum of **6j**.

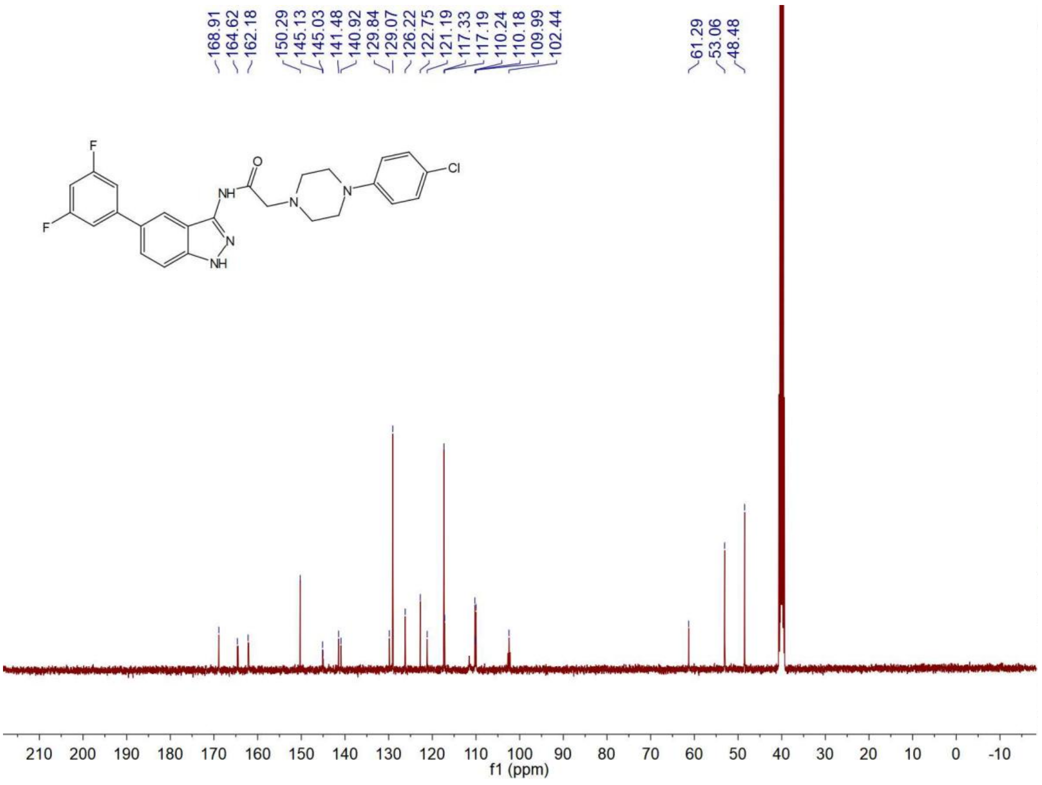

Figure S80. The ^{13}C NMR spectrum of **6j**.

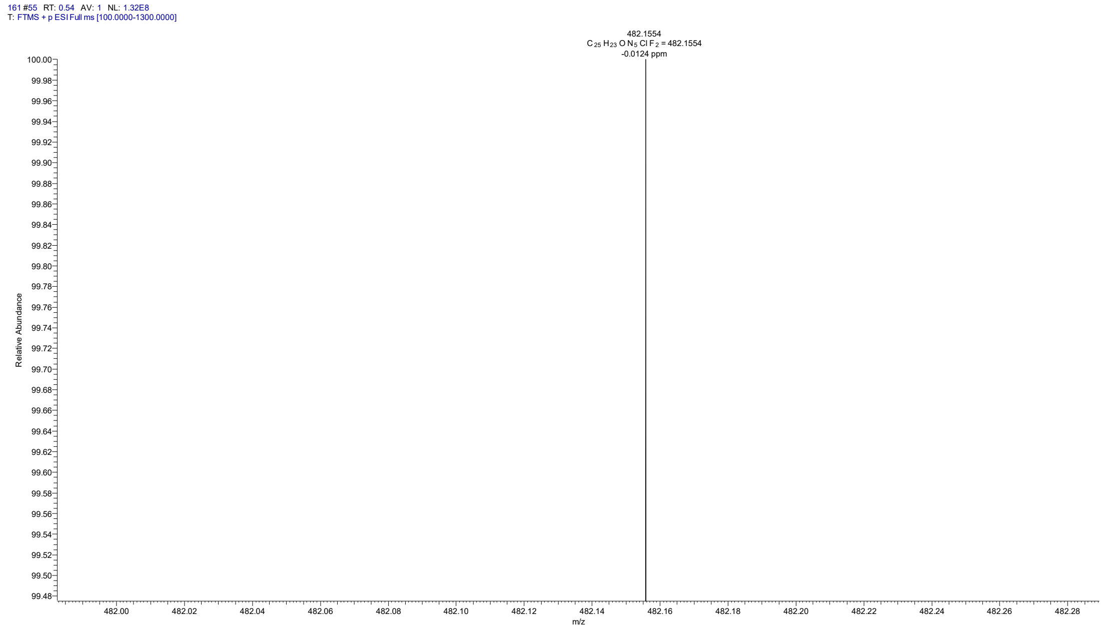

Figure S81. The HRMS spectrum of **6j**.

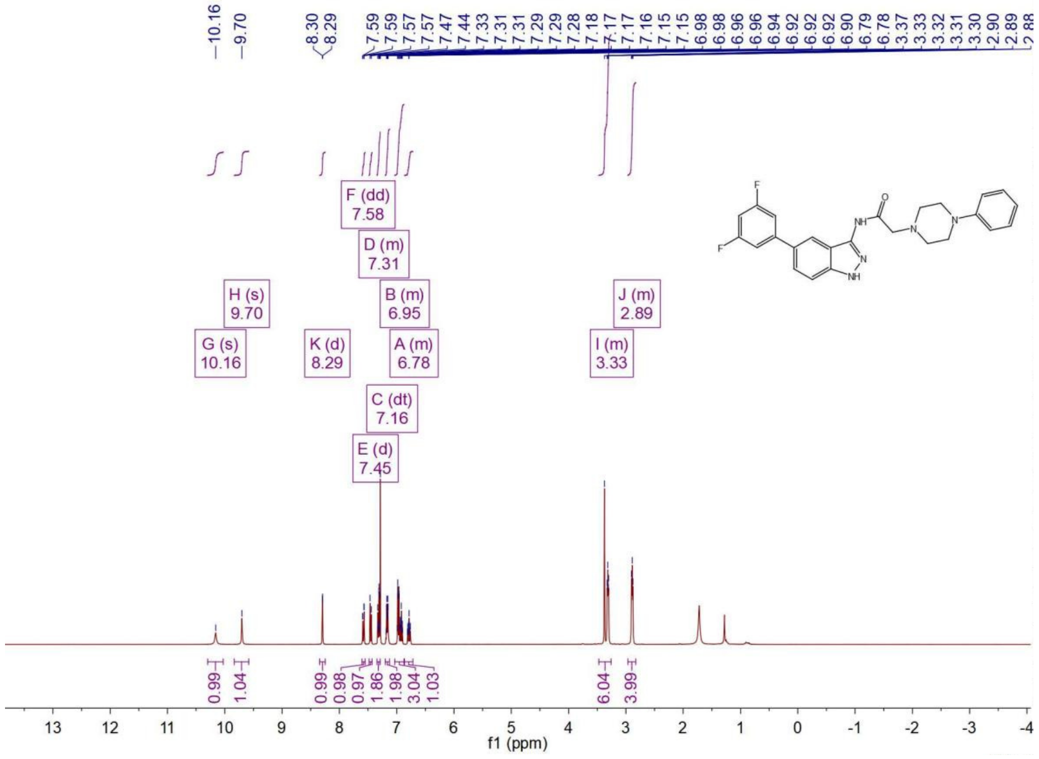

Figure S82. The ^{1}H NMR spectrum of **6k**.

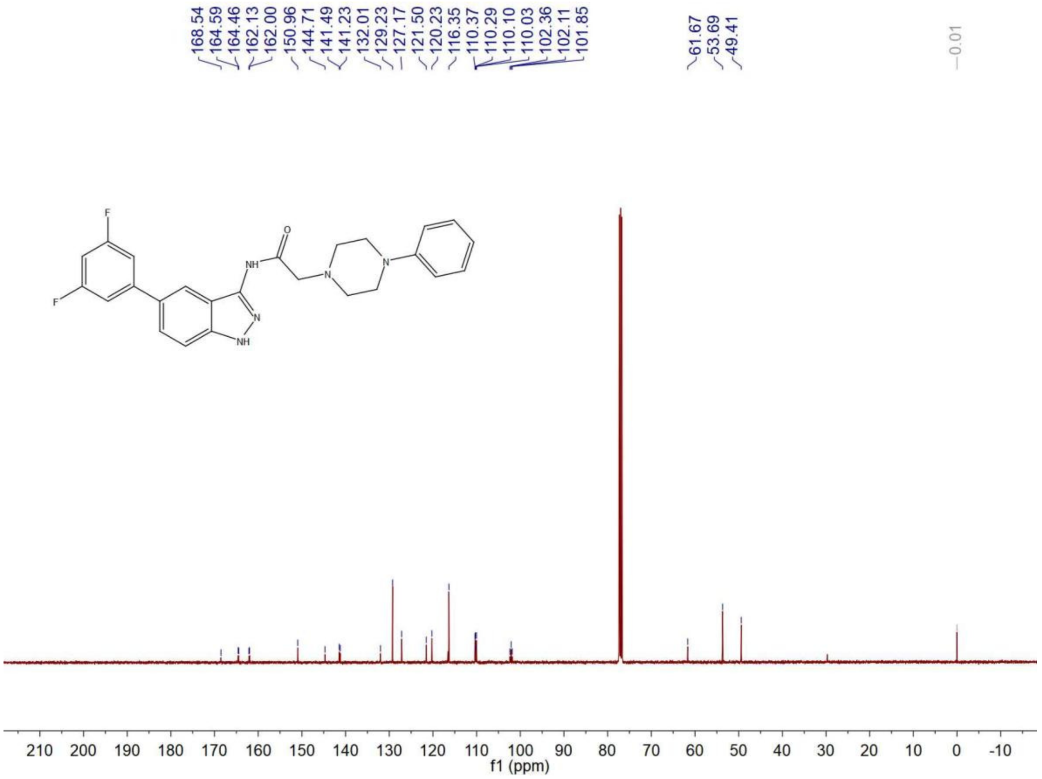

Figure S83. The ^{13}C NMR spectrum of **6k**.

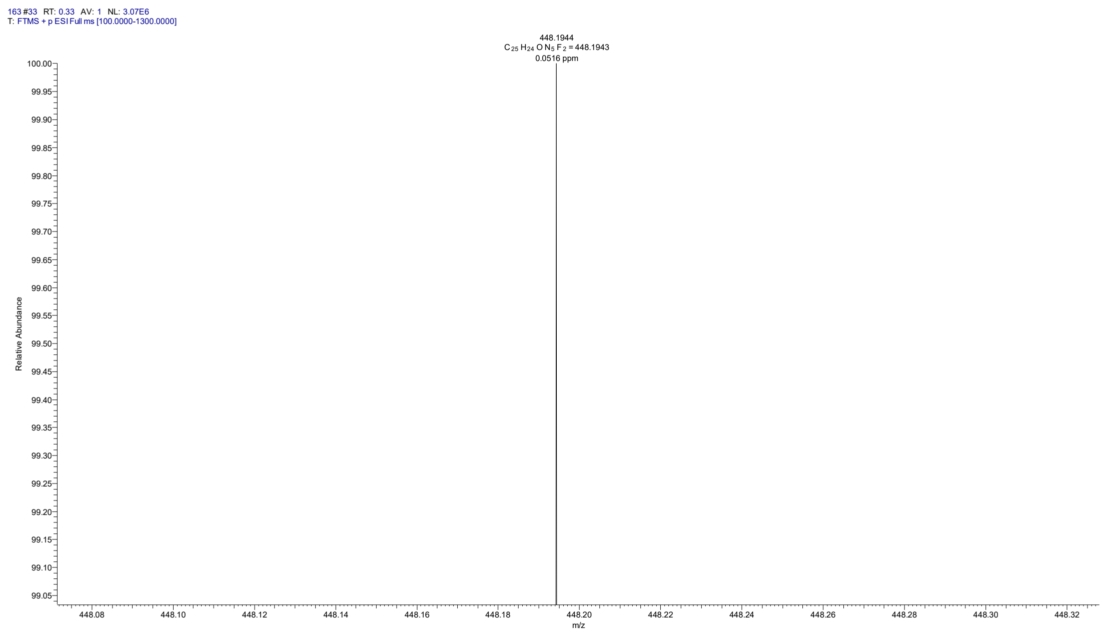

Figure S84. The HRMS spectrum of **6k**.

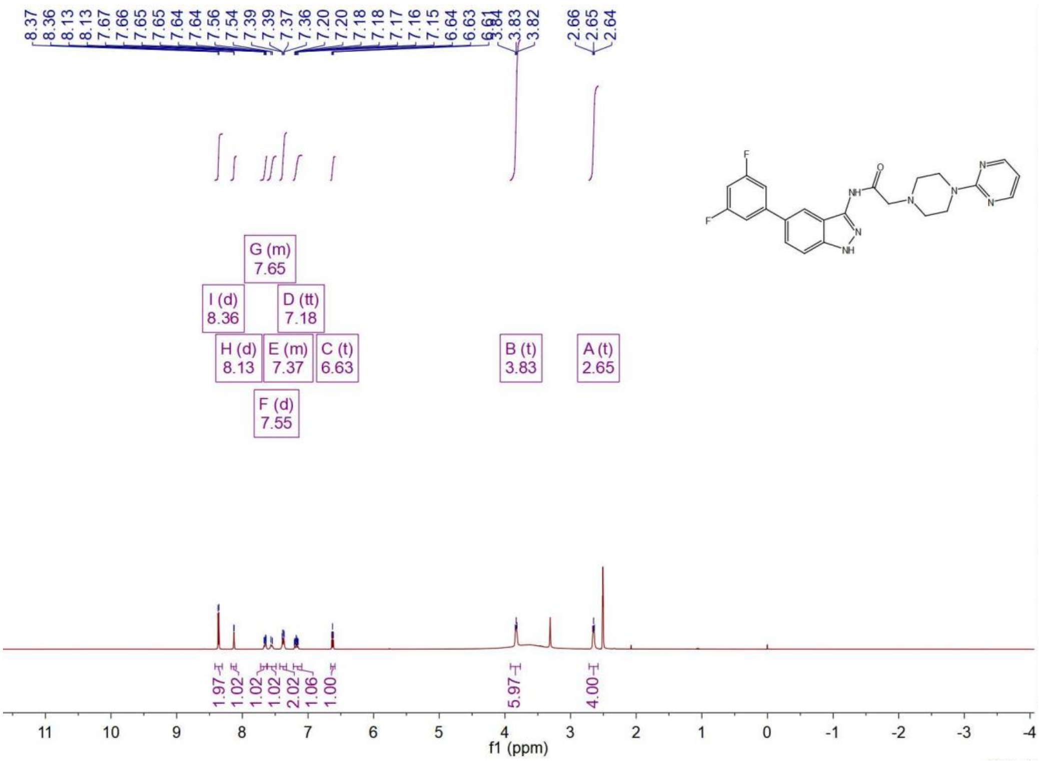

Figure S85. The ^{1}H NMR spectrum of **6l**.

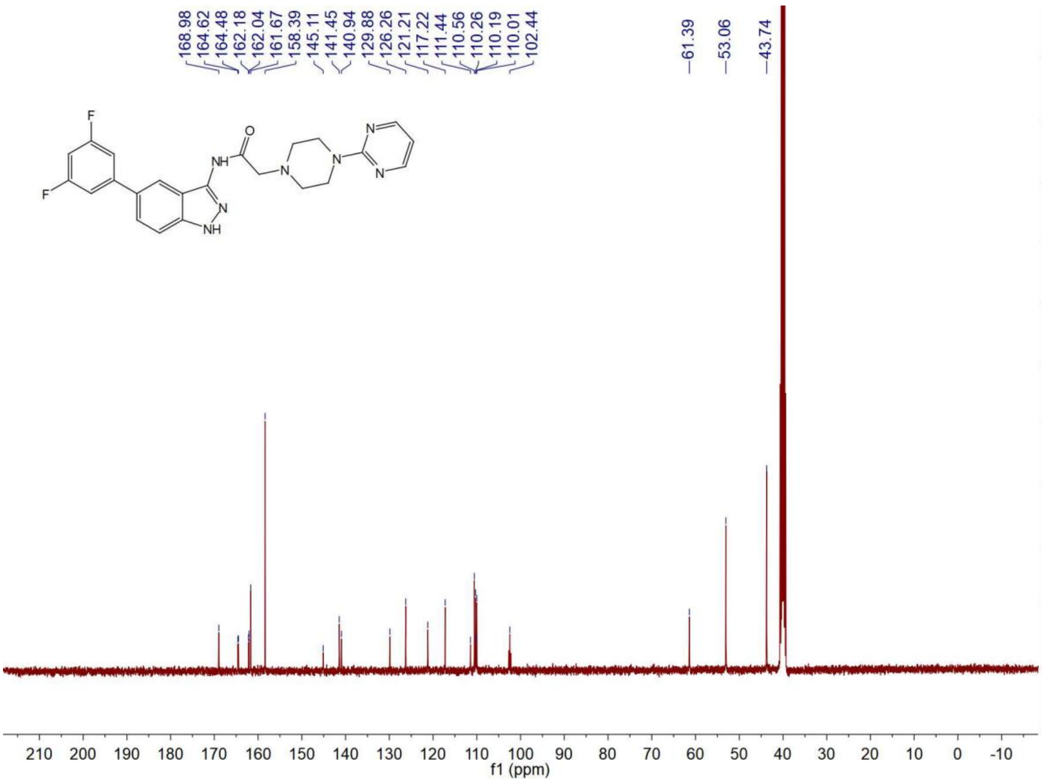

Figure S86. The ^{13}C NMR spectrum of **6l**.

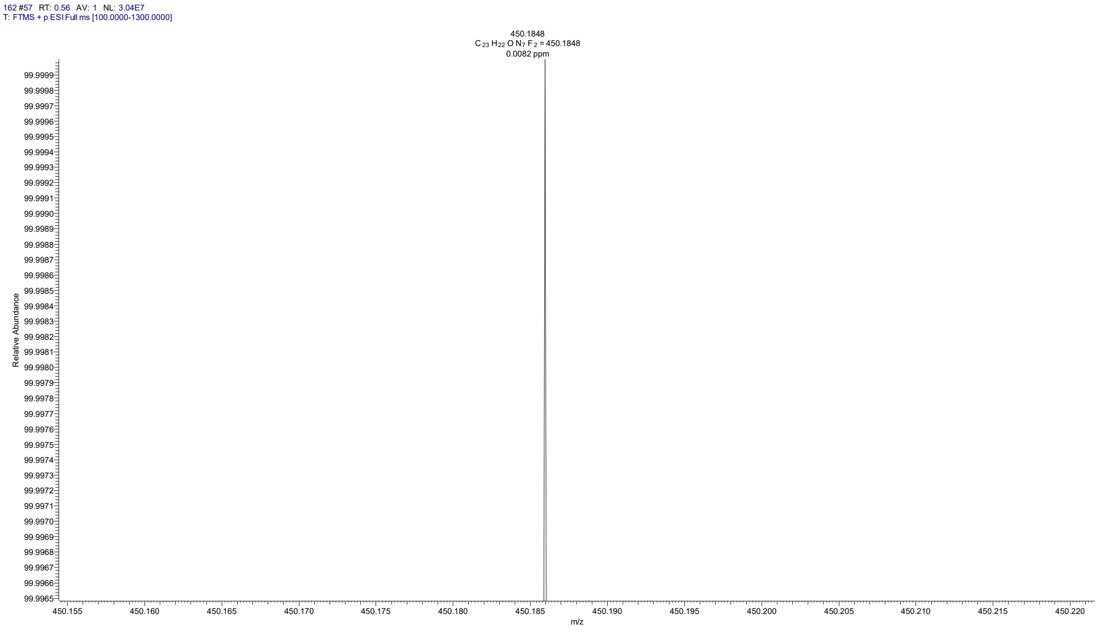

Figure S87. The HRMS spectrum of **6l**.

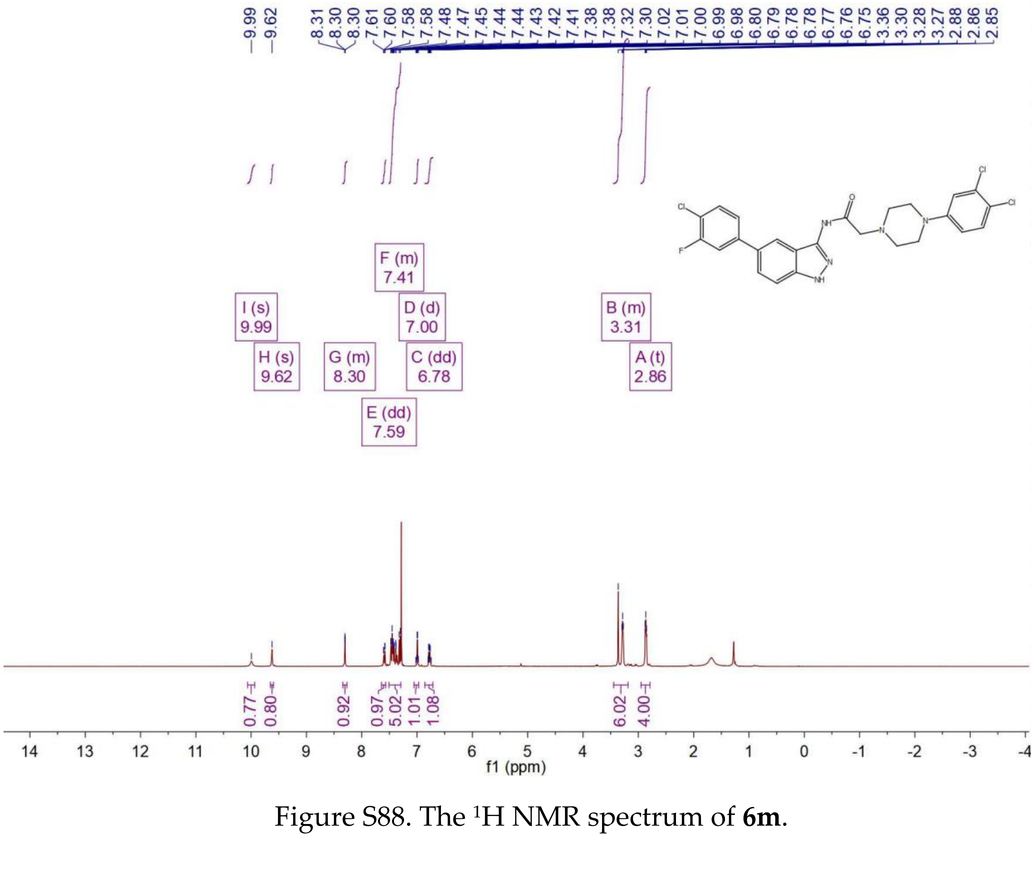

Figure S88. The ^{1}H NMR spectrum of **6m**.

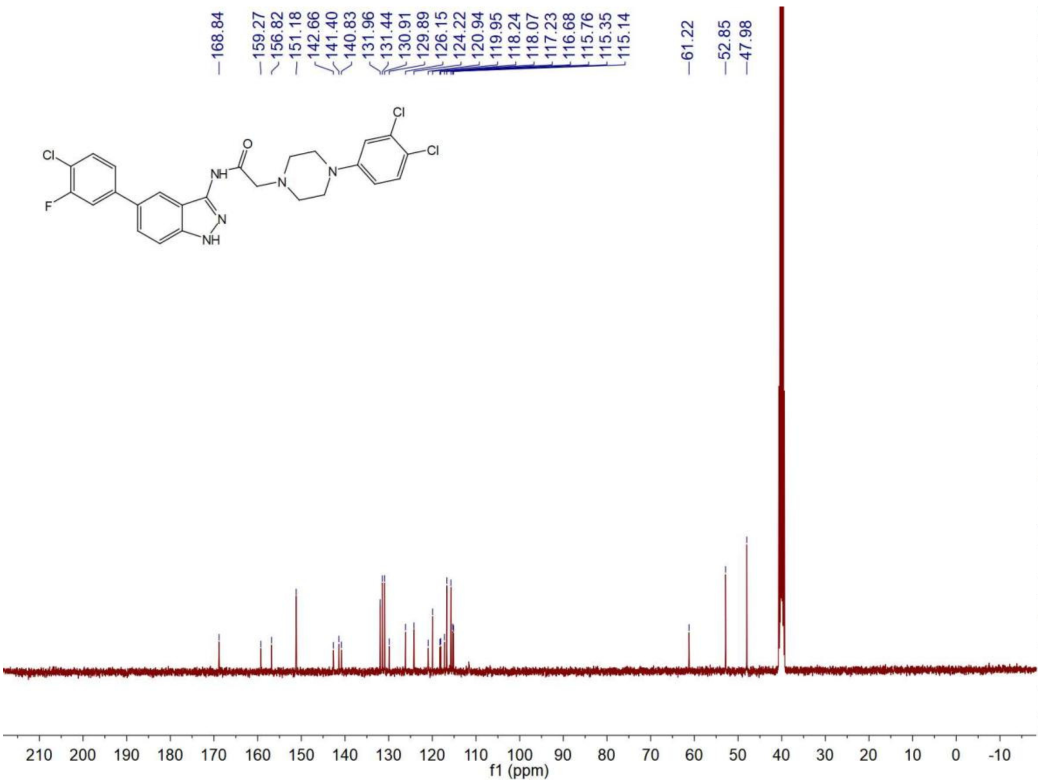

Figure S89. The ^{13}C NMR spectrum of **6m**.

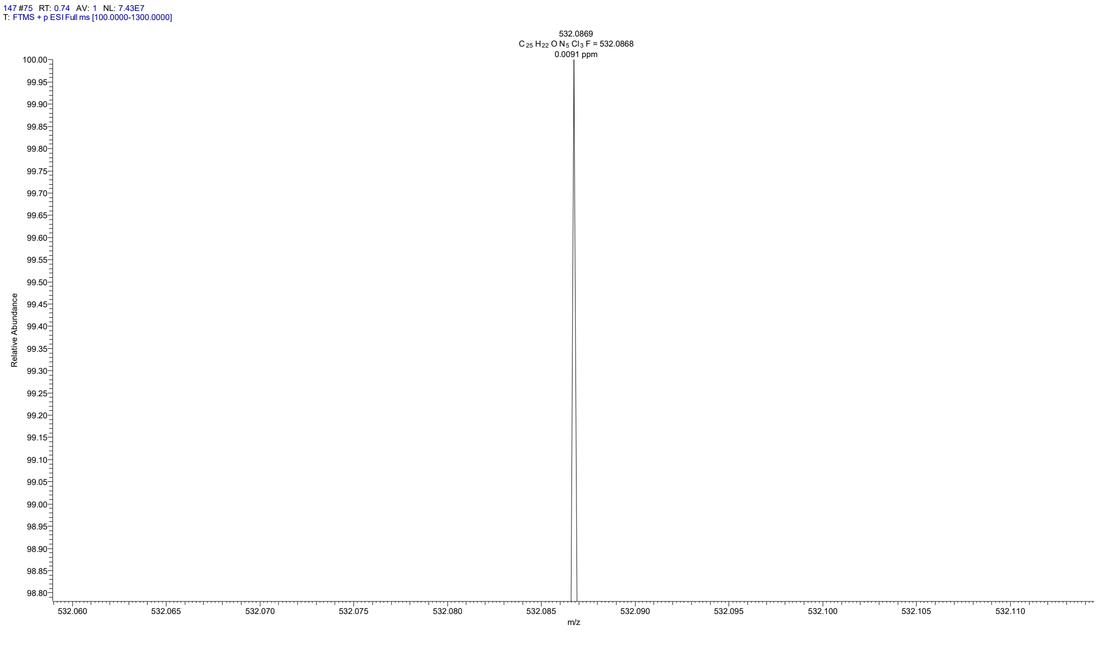

Figure S90. The HRMS spectrum of **6m**.

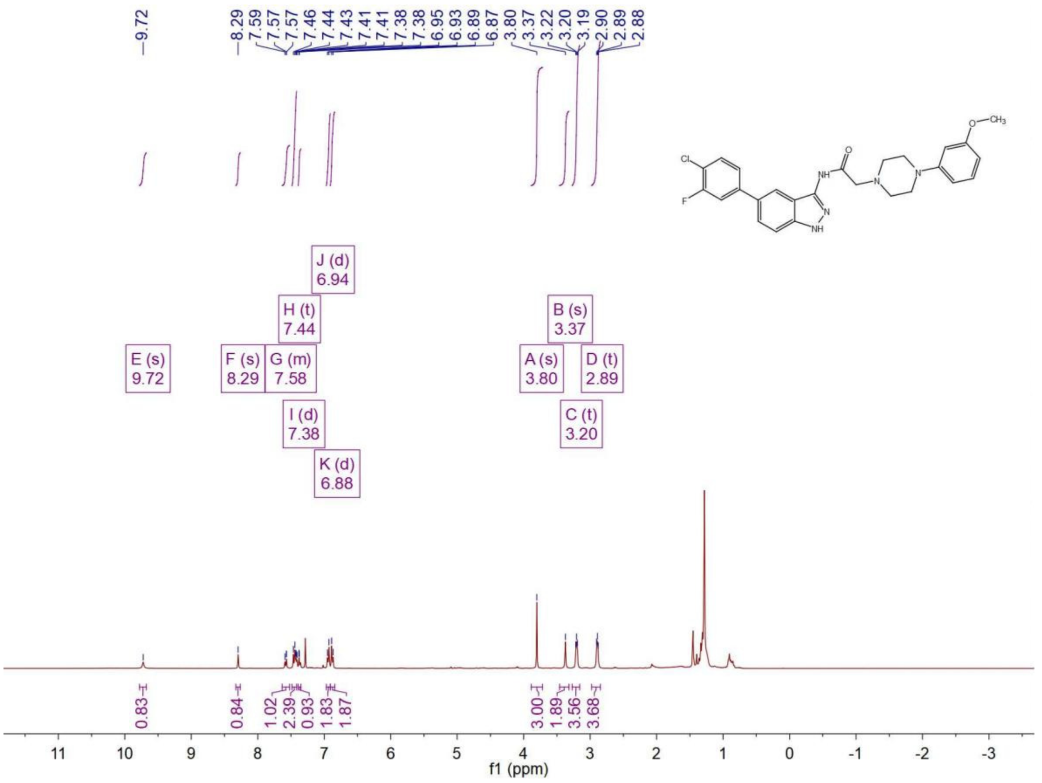

Figure S91. The ^{1}H NMR spectrum of **6n**.

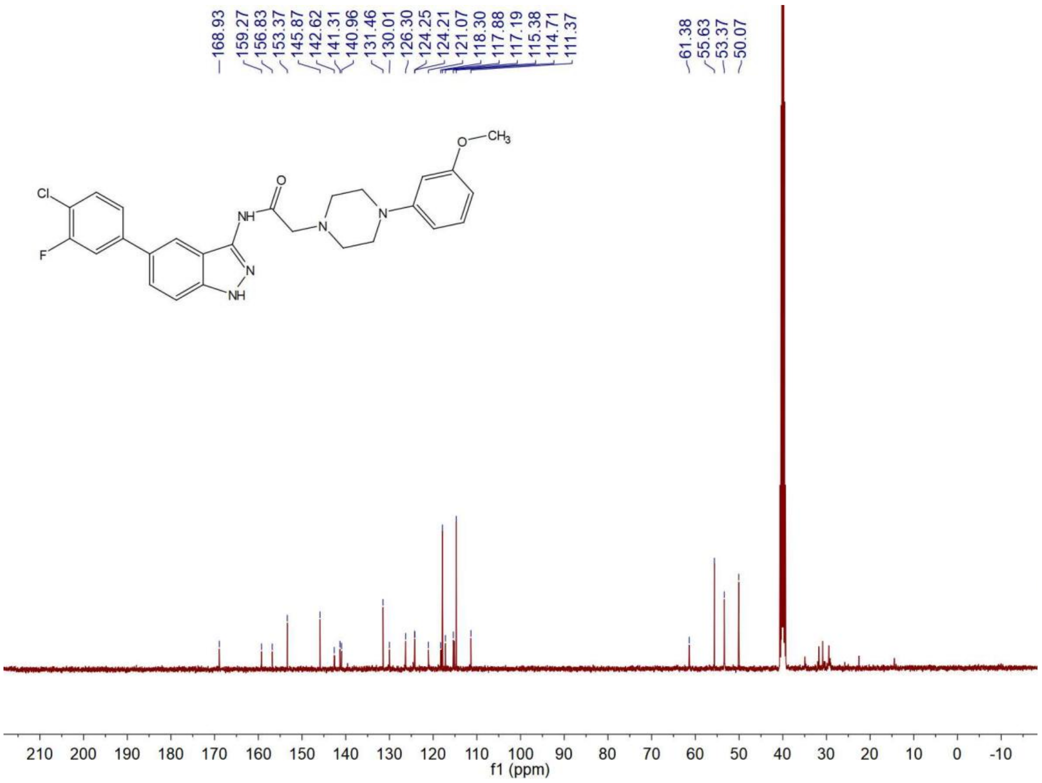

Figure S92. The ^{13}C NMR spectrum of **6n**.

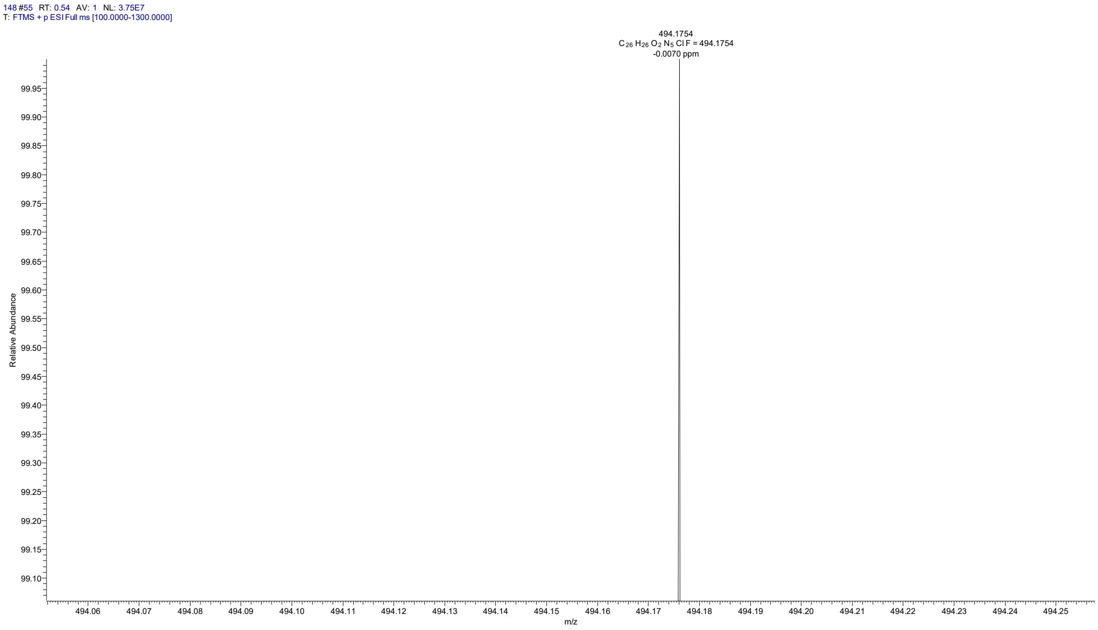

Figure S93. The HRMS spectrum of **6n**.

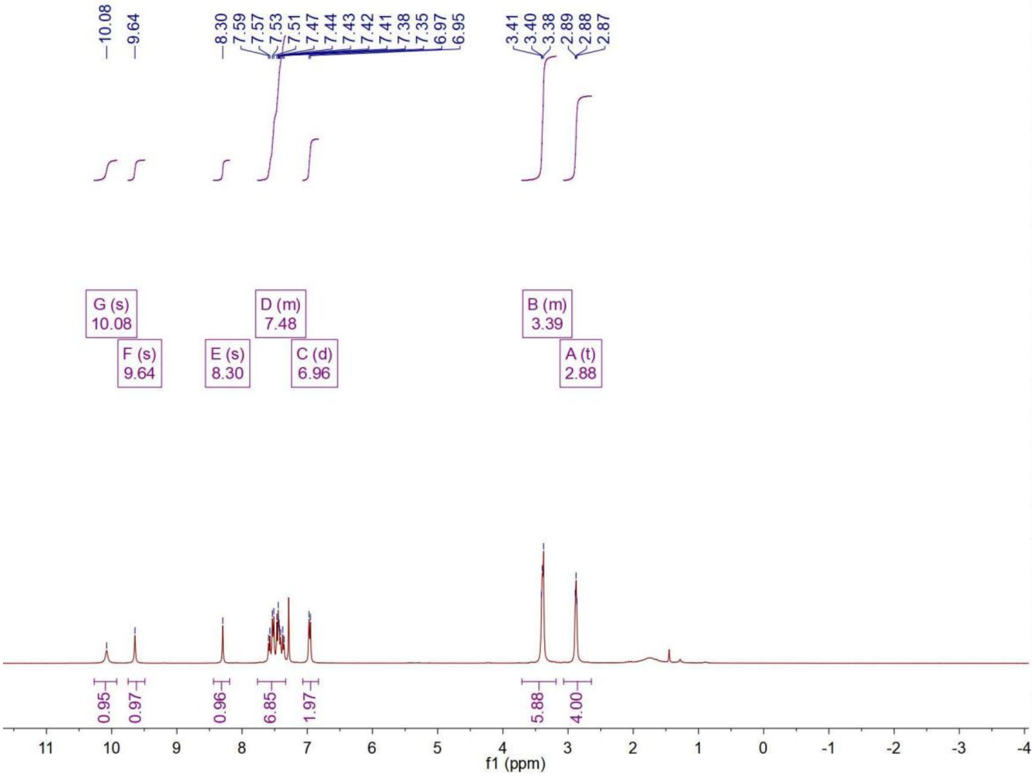

Figure S94. The ^{1}H NMR spectrum of **6o**.

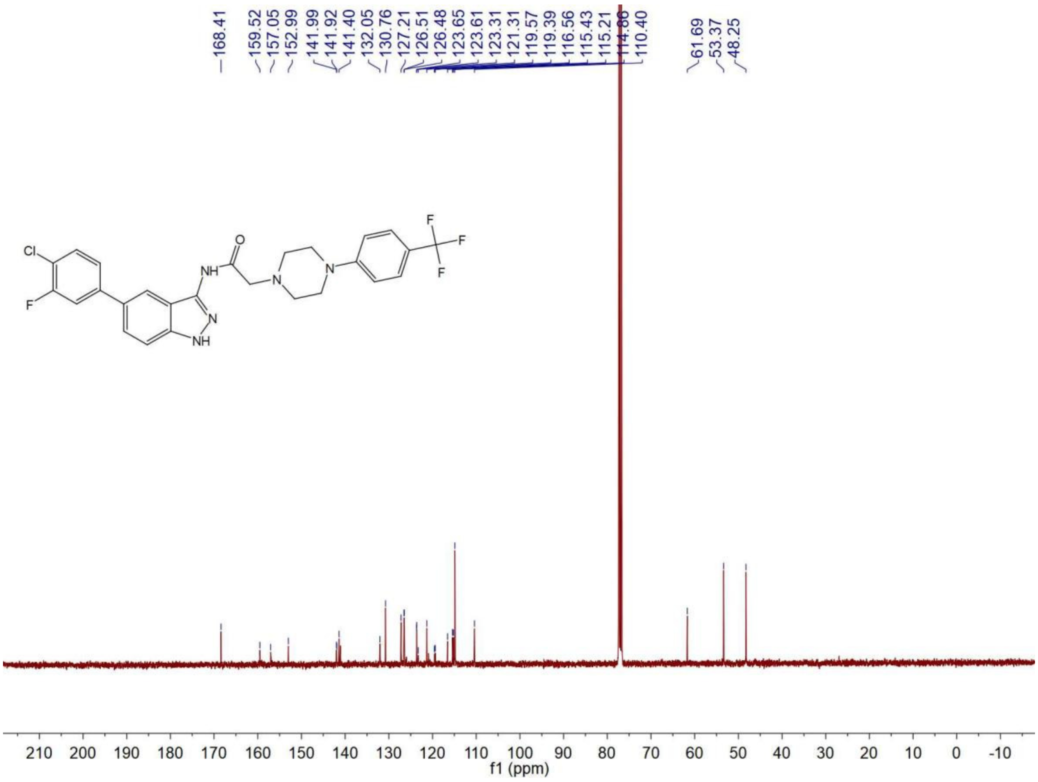

Figure S95. The ^{13}C NMR spectrum of **6o**.

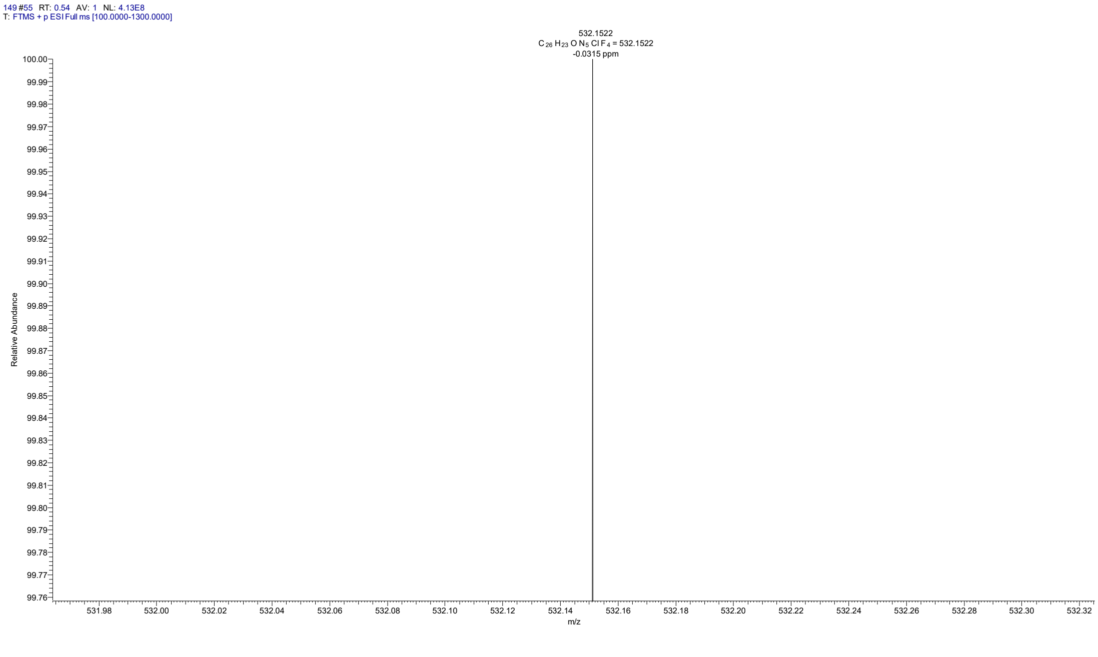

Figure S96. The HRMS spectrum of **6o**.

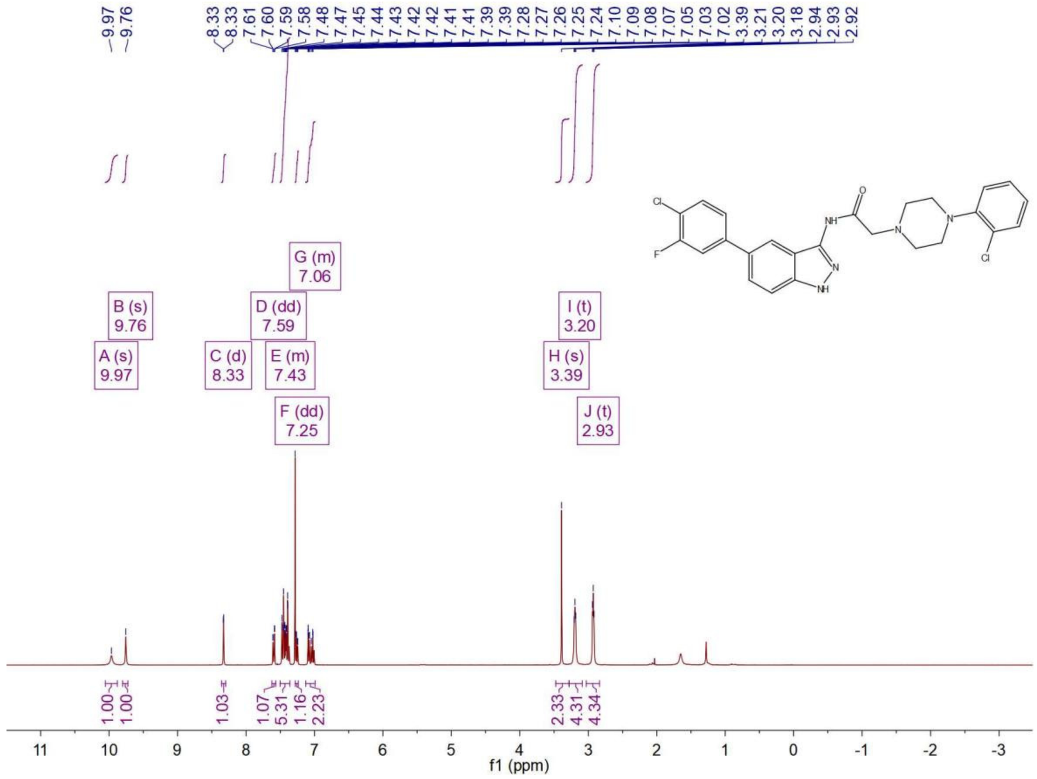

Figure S97. The ^{1}H NMR spectrum of **6p**.

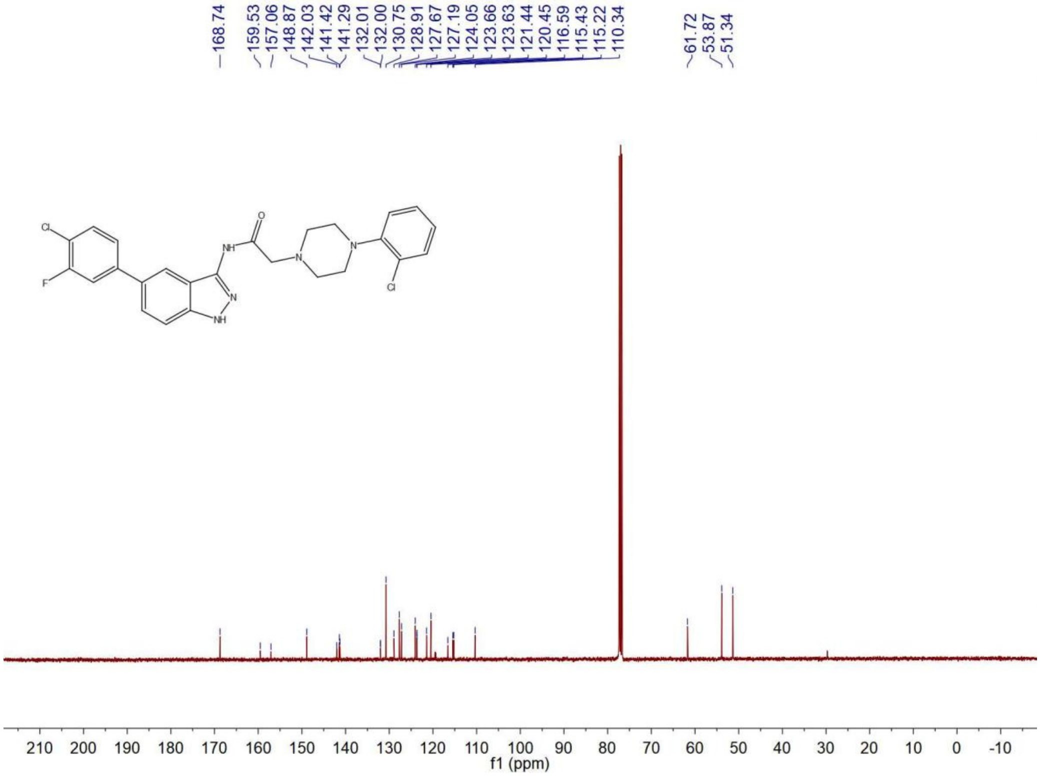

Figure S98. The ^{13}C NMR spectrum of **6p**.

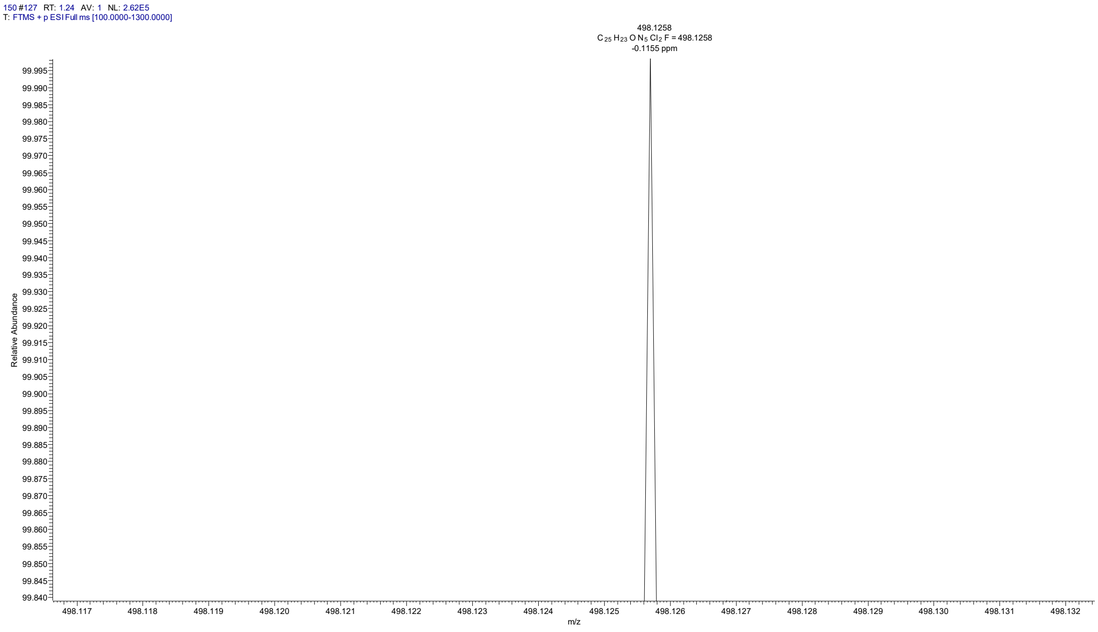

Figure S99. The HRMS spectrum of **6p**.

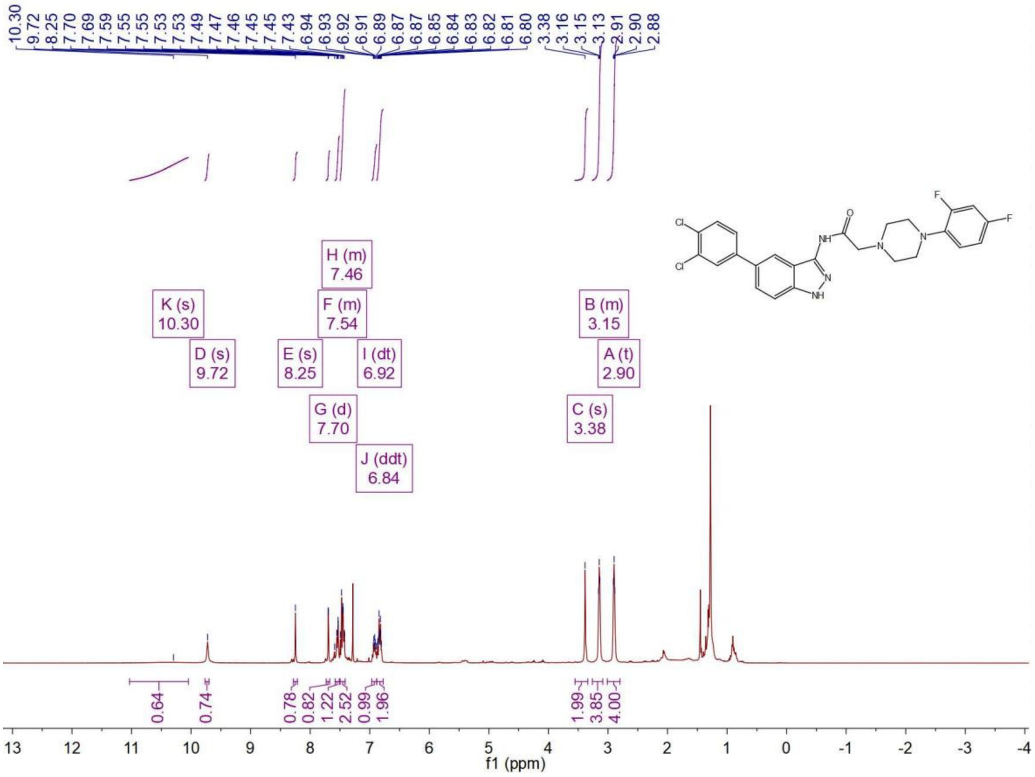

Figure S100. The ^{1}H NMR spectrum of **6q**.

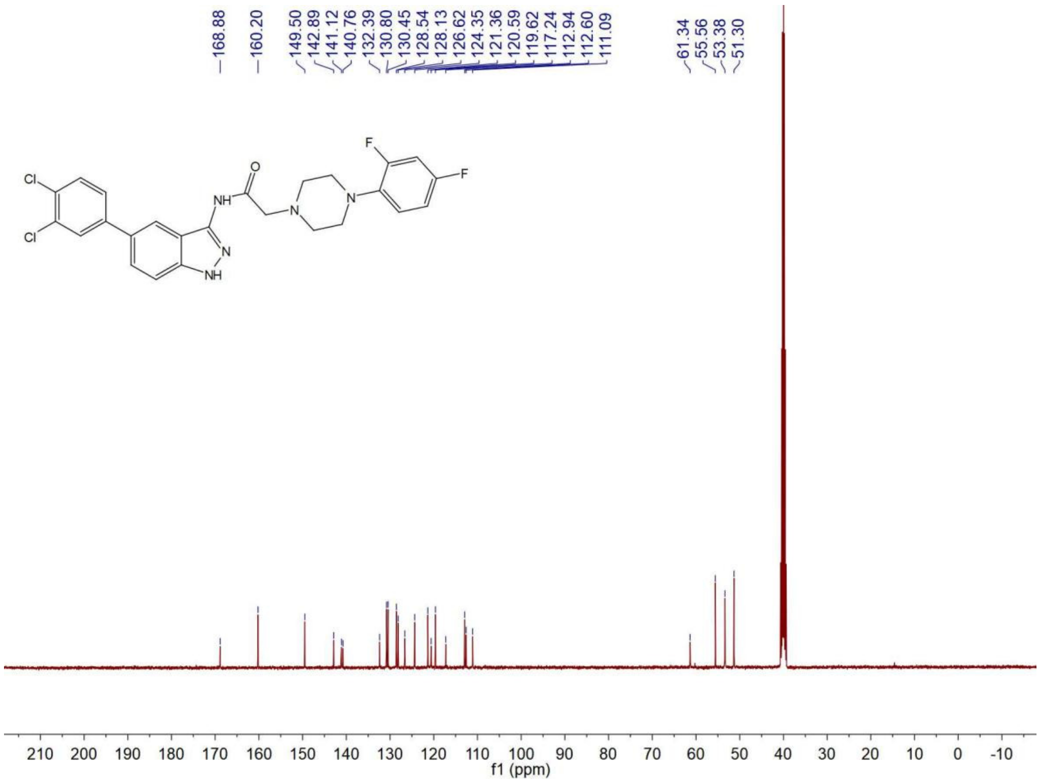

Figure S101. The ^{13}C NMR spectrum of **6q**.

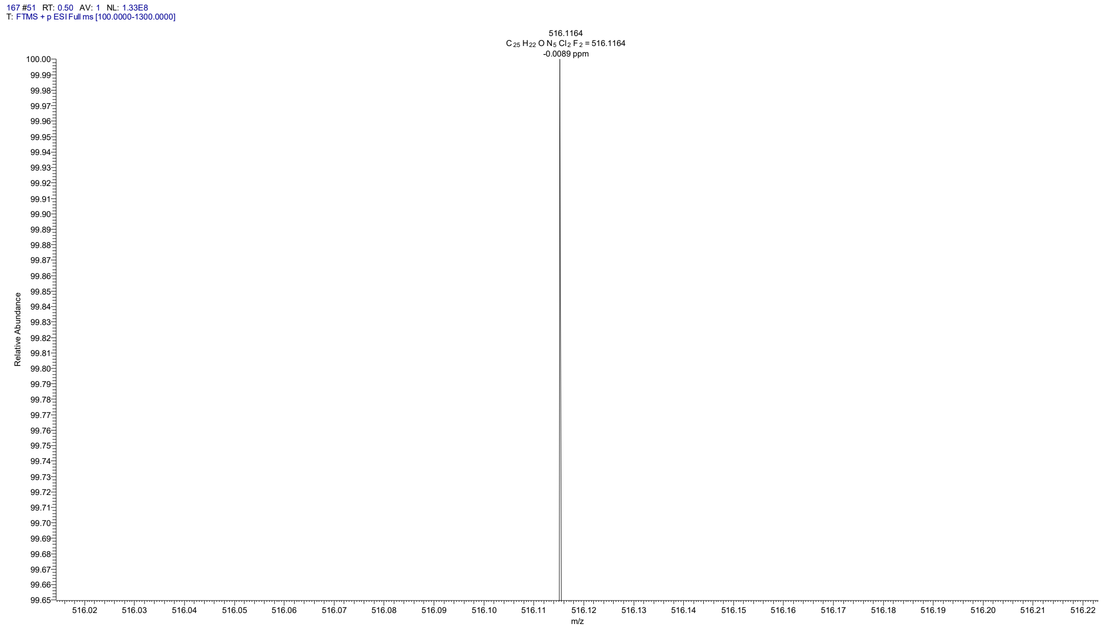

Figure S102. The HRMS spectrum of **6q**.

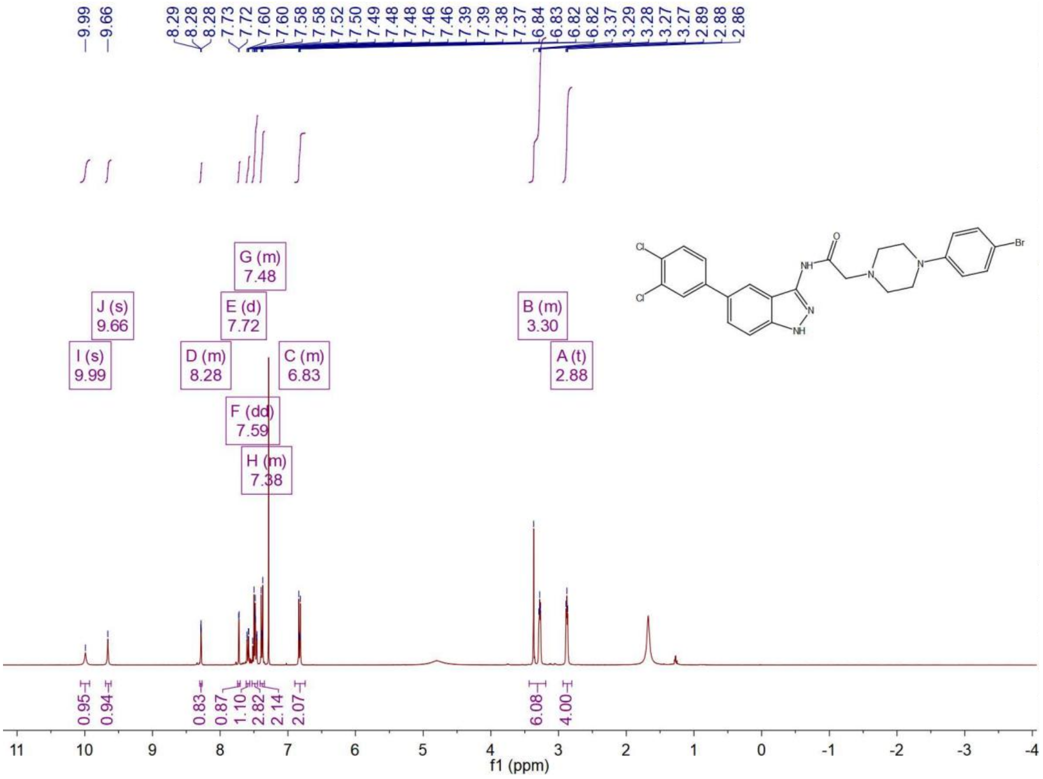

Figure S103. The ^{1}H NMR spectrum of **6r**.

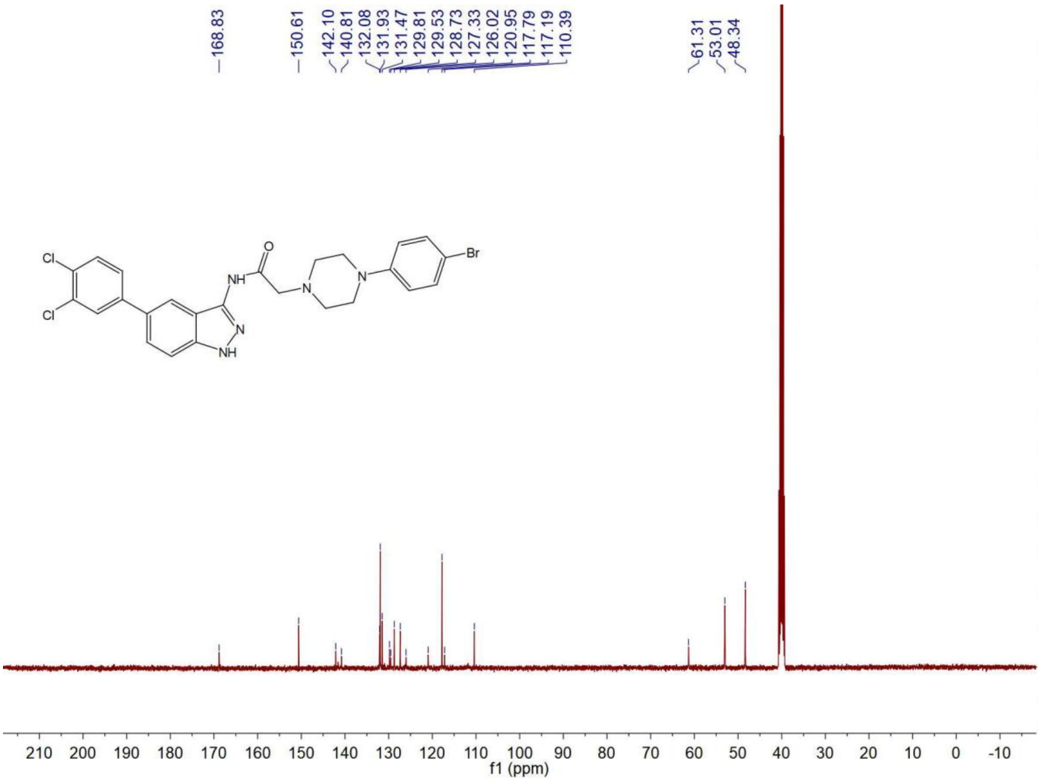

Figure S104. The ^{13}C NMR spectrum of **6r**.

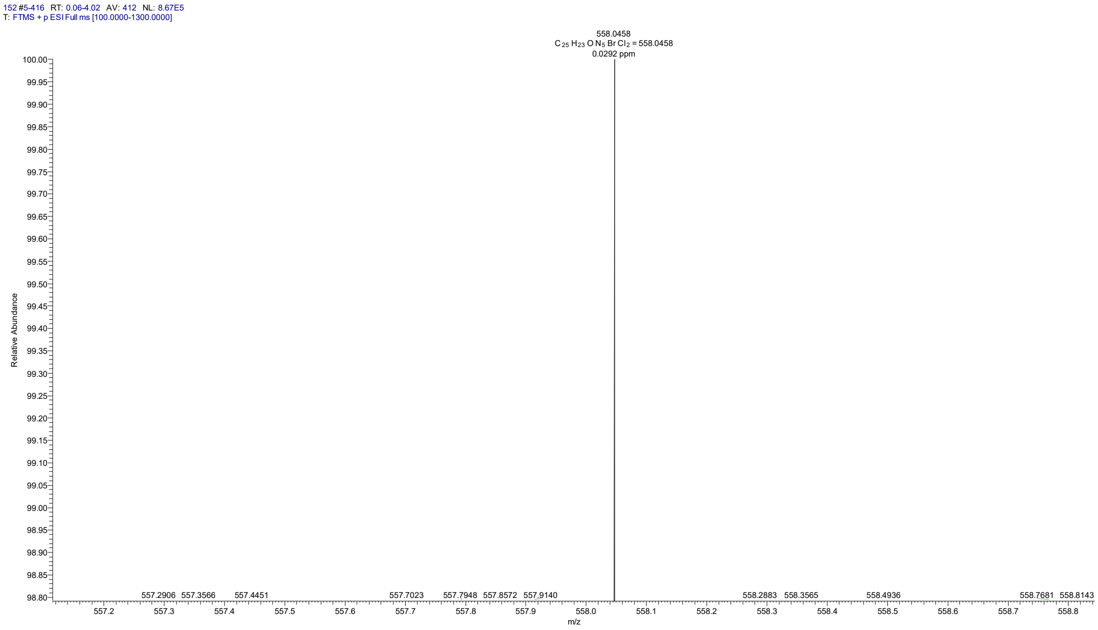

Figure S105. The HRMS spectrum of **6r**.

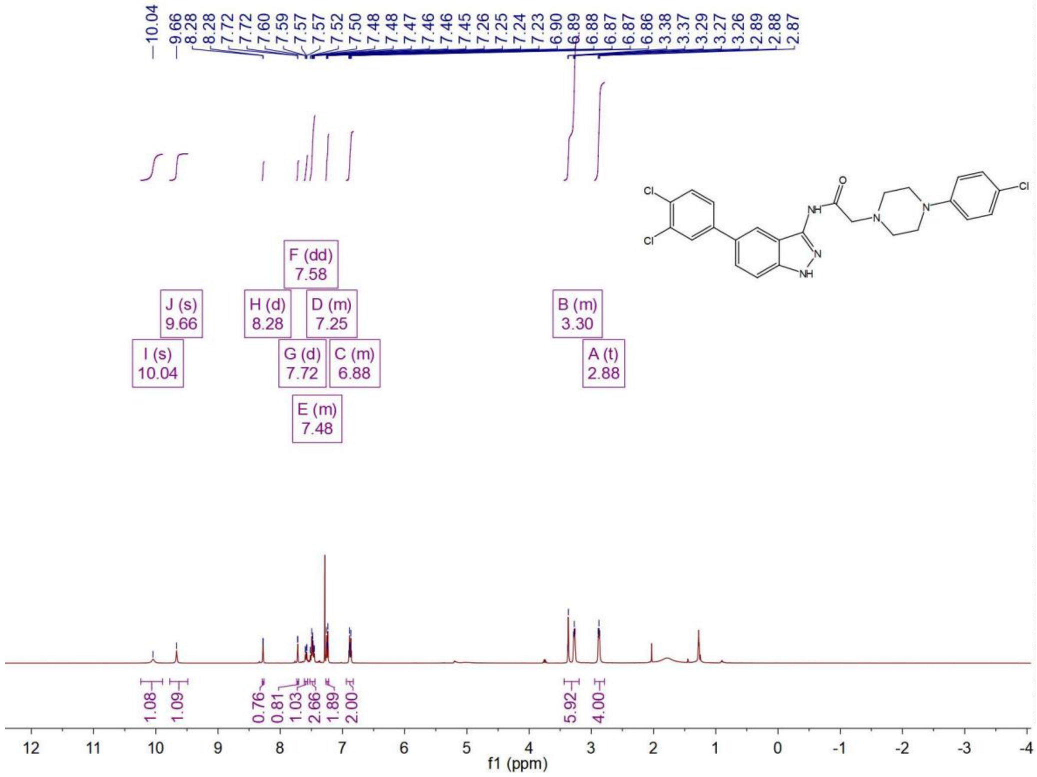

Figure S106. The ^{1}H NMR spectrum of **6s**.

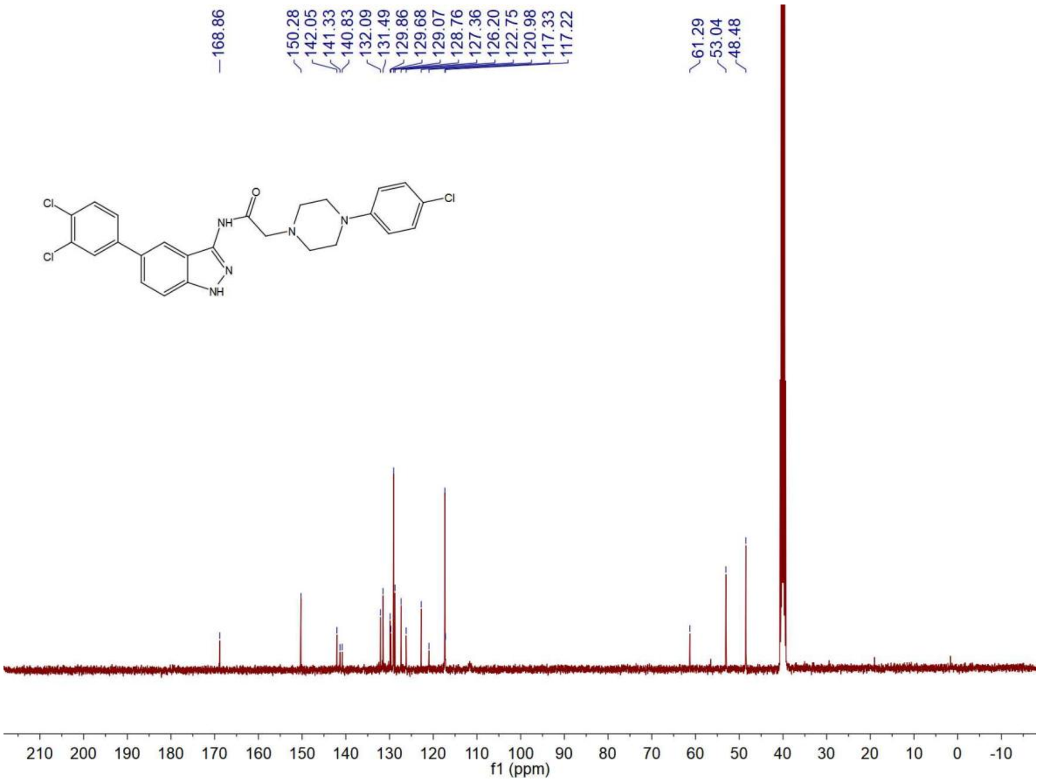

Figure S107. The ^{13}C NMR spectrum of **6s**.

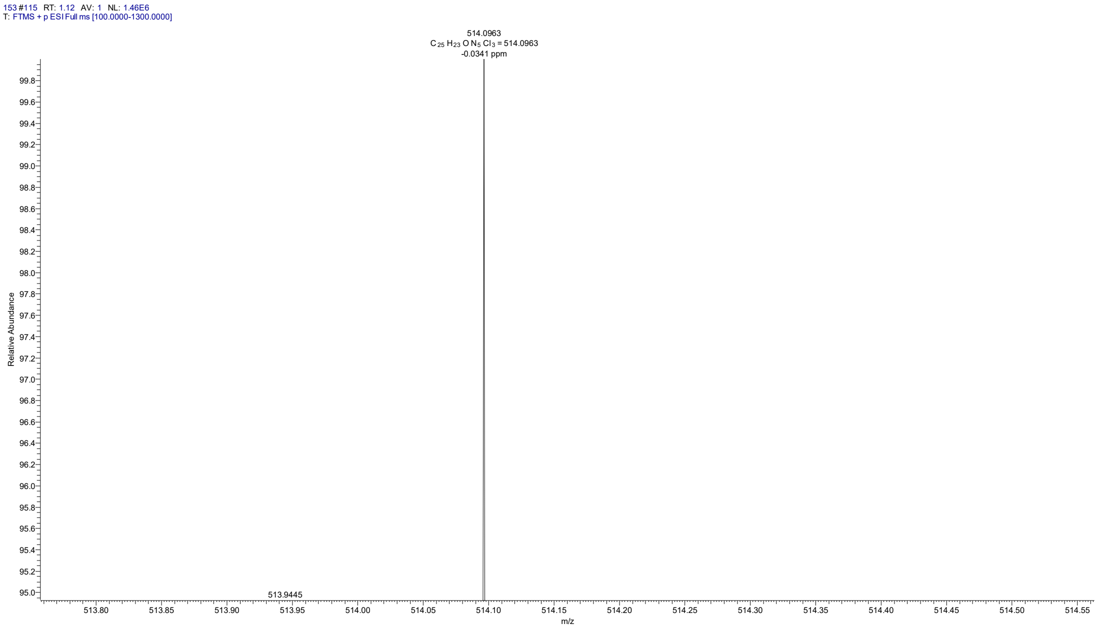

Figure S108. The HRMS spectrum of **6s**.

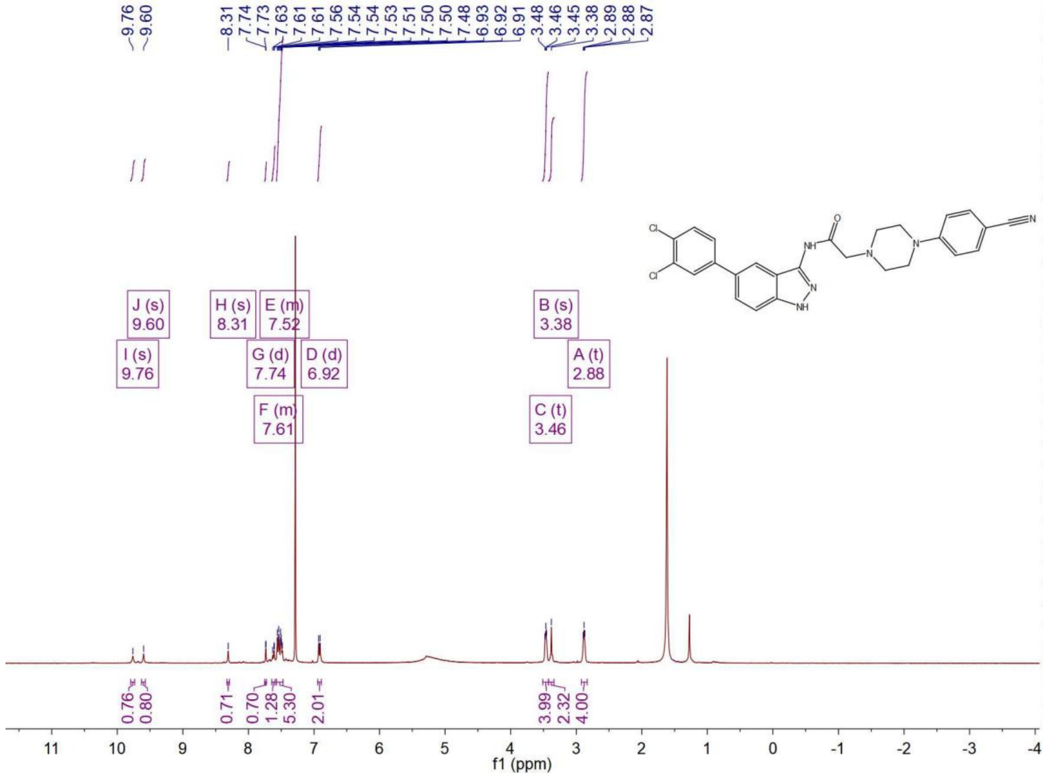

Figure S109. The ^{1}H NMR spectrum of **6t**.

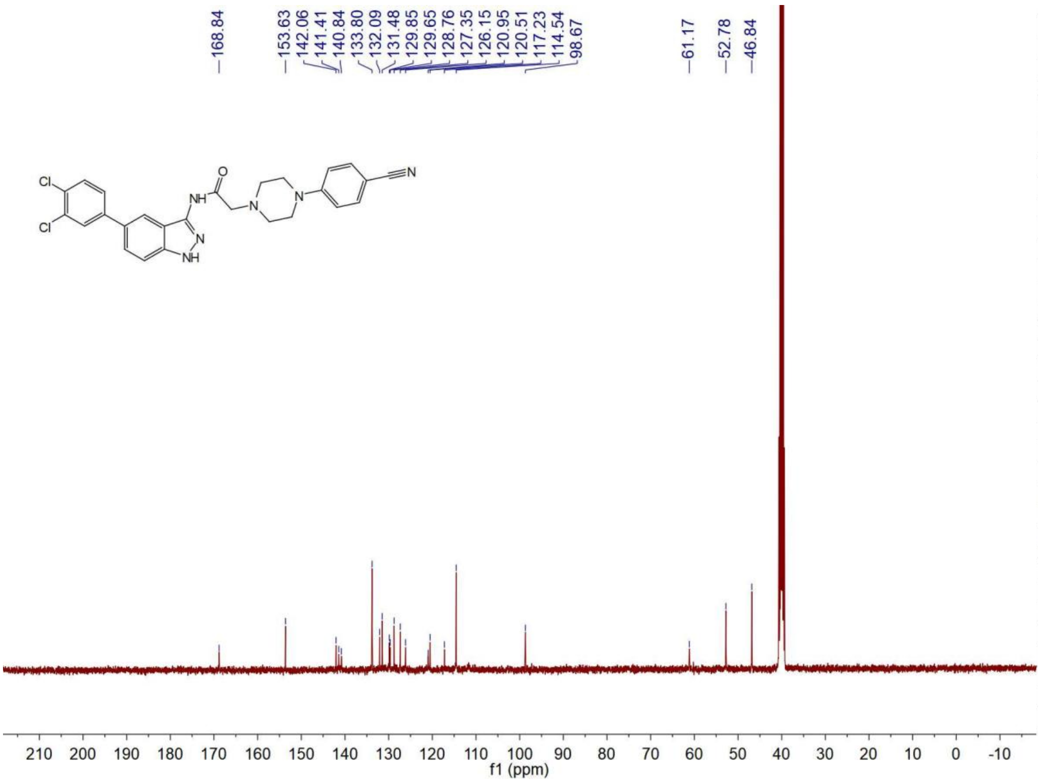

Figure S110. The ^{13}C NMR spectrum of **6t**.

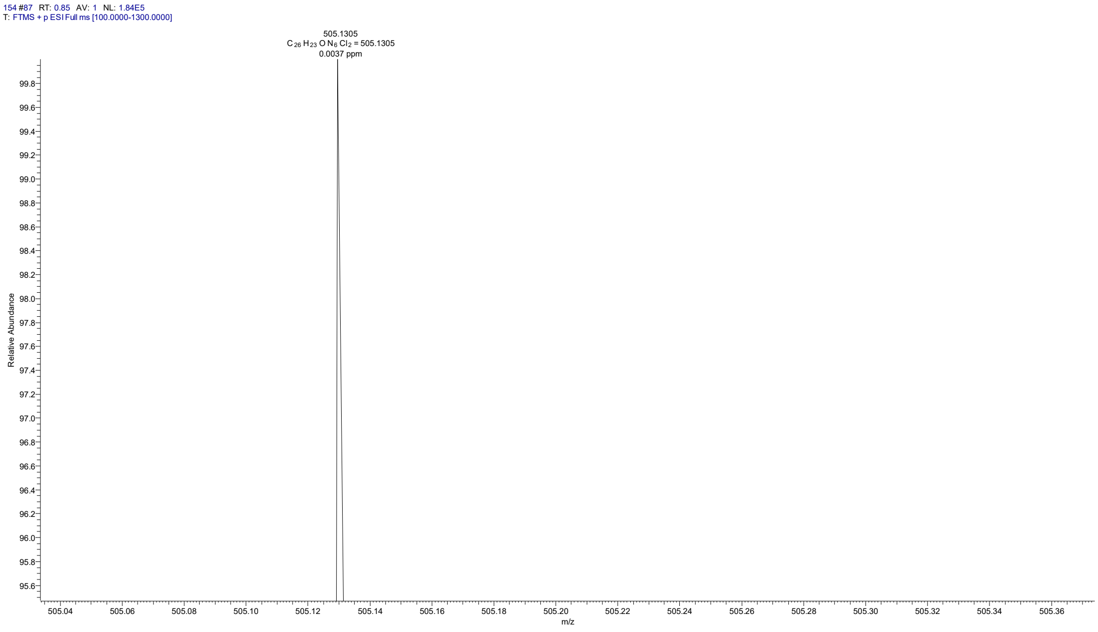

Figure S111. The HRMS spectrum of **6t**.

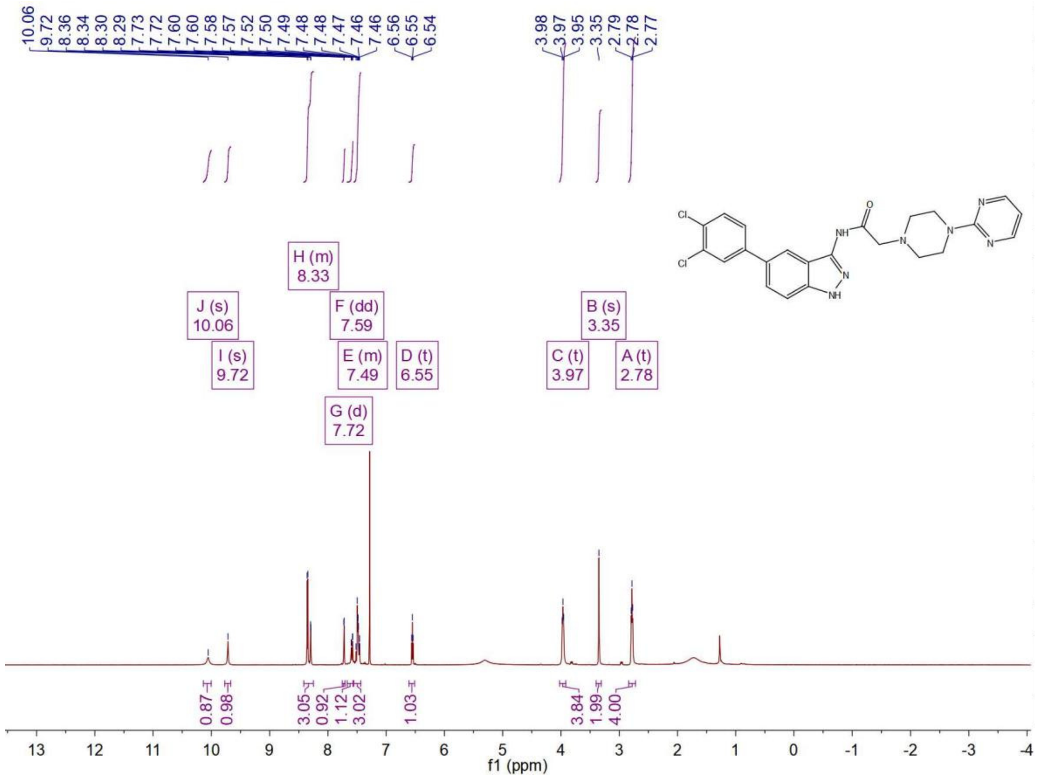

Figure S112. The ^{1}H NMR spectrum of **6u**.

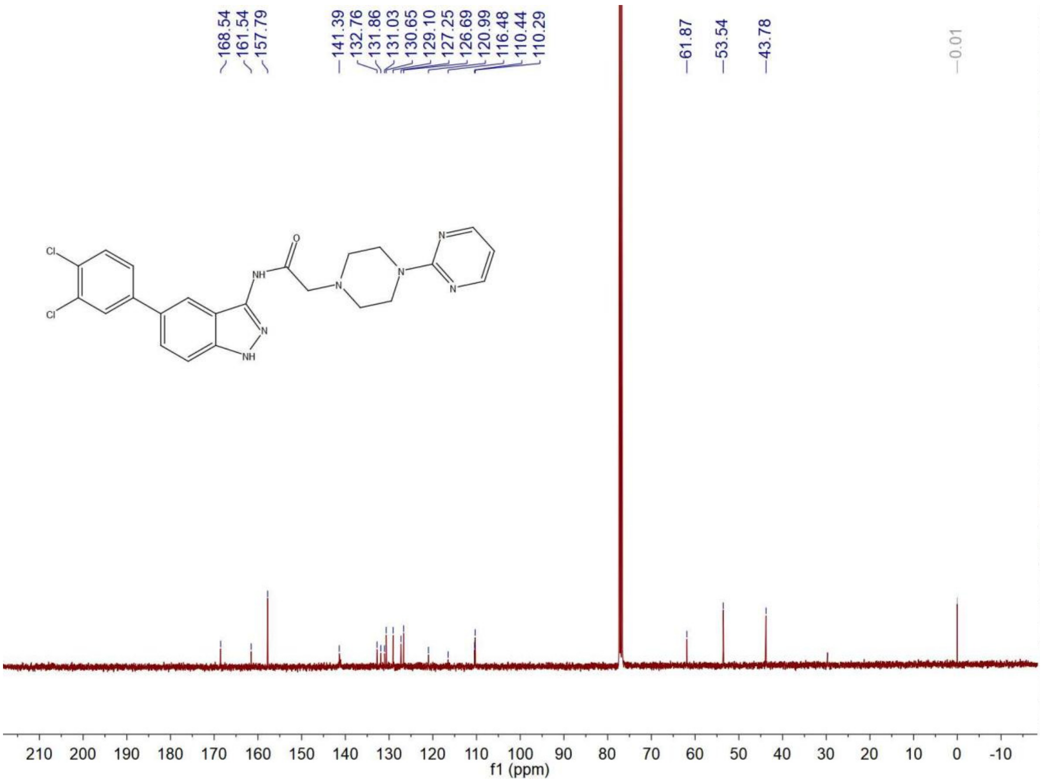

Figure S113. The ^{13}C NMR spectrum of **6u**.

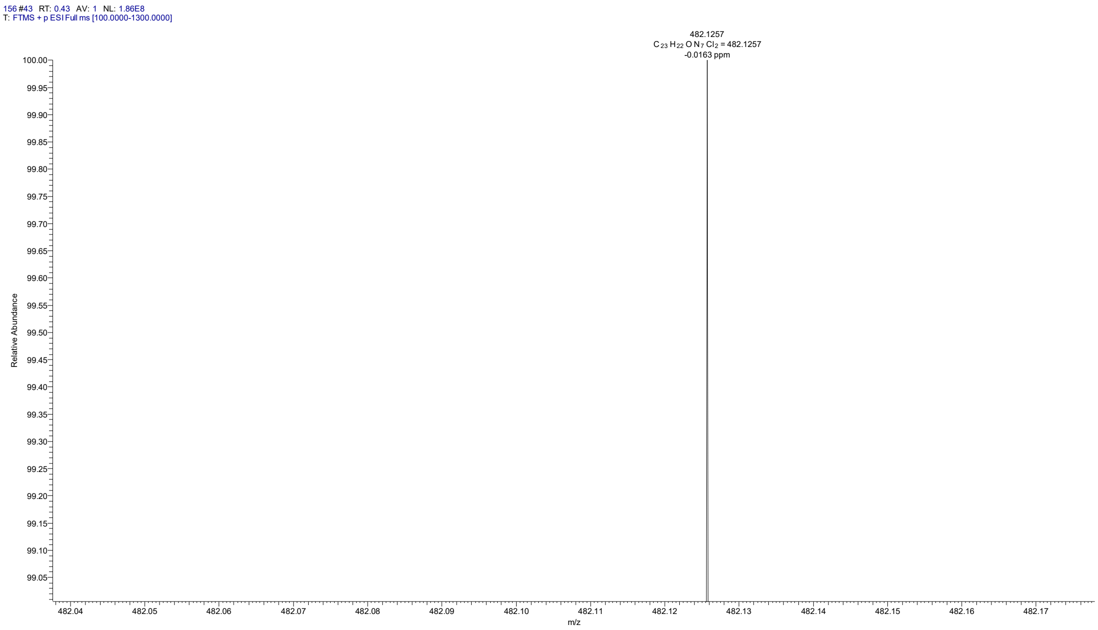

Figure S114. The HRMS spectrum of **6u**.

8. The graphs for IC_{50} of compound 6o in K562.

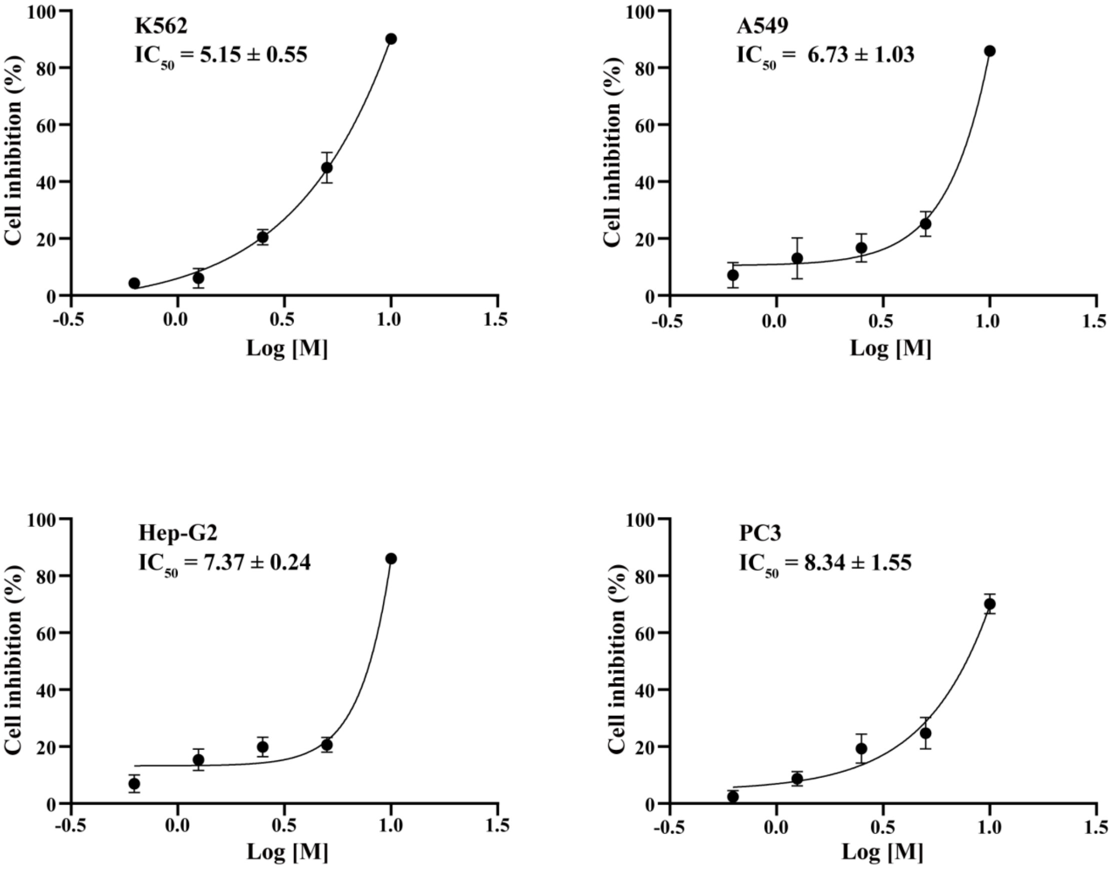

Figure S115. The graphs for IC_{50} in K562, A549, Hep-G2 and PC-3 cell lines for the compound **6o**.
